# Systematic review of accelerometer-based methods for 24-h physical behavior assessment in young children (0–5 years old)

**DOI:** 10.1186/s12966-022-01296-y

**Published:** 2022-09-08

**Authors:** Annelinde Lettink, Teatske M. Altenburg, Jelle Arts, Vincent T. van Hees, Mai J. M. Chinapaw

**Affiliations:** 1 Amsterdam UMC Location Vrije Universiteit Amsterdam, Public and Occupational Health, De Boelelaan 1117, Amsterdam, The Netherlands; 2 Amsterdam Public Health, Methodology, Amsterdam, The Netherlands; 3 Amsterdam Public Health, Health Behaviors & Chronic Diseases, Amsterdam, The Netherlands; 4 Accelting, Almere, The Netherlands

**Keywords:** 24-h physical behavior, Physical activity, Sedentary behavior, Sleep, Infants, Toddlers, Preschoolers, Accelerometer, Cut-points, Machine learning, Measurement properties, Validity, Reliability

## Abstract

**Background:**

Accurate accelerometer-based methods are required for assessment of 24-h physical behavior in young children. We aimed to summarize evidence on measurement properties of accelerometer-based methods for assessing 24-h physical behavior in young children.

**Methods:**

We searched PubMed (MEDLINE) up to June 2021 for studies evaluating reliability or validity of accelerometer-based methods for assessing physical activity (PA), sedentary behavior (SB), or sleep in 0–5-year-olds. Studies using a subjective comparison measure or an accelerometer-based device that did not directly output time series data were excluded. We developed a Checklist for Assessing the Methodological Quality of studies using Accelerometer-based Methods (CAMQAM) inspired by COnsensus-based Standards for the selection of health Measurement INstruments (COSMIN).

**Results:**

Sixty-two studies were included, examining conventional cut-point-based methods or multi-parameter methods. For infants (0—12 months), several multi-parameter methods proved valid for classifying SB and PA. From three months of age, methods were valid for identifying sleep. In toddlers (1—3 years), cut-points appeared valid for distinguishing SB and light PA (LPA) from moderate-to-vigorous PA (MVPA). One multi-parameter method distinguished toddler specific SB. For sleep, no studies were found in toddlers. In preschoolers (3—5 years), valid hip and wrist cut-points for assessing SB, LPA, MVPA, and wrist cut-points for sleep were identified. Several multi-parameter methods proved valid for identifying SB, LPA, and MVPA, and sleep.

Despite promising results of multi-parameter methods, few models were open-source. While most studies used a single device or axis to measure physical behavior, more promising results were found when combining data derived from different sensor placements or multiple axes.

**Conclusions:**

Up to age three, valid cut-points to assess 24-h physical behavior were lacking, while multi-parameter methods proved valid for distinguishing some waking behaviors. For preschoolers, valid cut-points and algorithms were identified for all physical behaviors. Overall, we recommend more high-quality studies evaluating 24-h accelerometer data from multiple sensor placements and axes for physical behavior assessment. Standardized protocols focusing on including well-defined physical behaviors in different settings representative for children’s developmental stage are required. Using our CAMQAM checklist may further improve methodological study quality.

**PROSPERO Registration number:**

CRD42020184751.

**Supplementary Information:**

The online version contains supplementary material available at 10.1186/s12966-022-01296-y.

## Introduction

Accurate assessment of 24-h physical behavior in young children is crucial as it provides the basis for examining the health benefits of these behaviors and thereby evidence for establishing 24-h movement guidelines. Recent studies indicated the importance of an integrated approach to all 24-h physical behaviors for health, encompassing sleep, sedentary behavior (SB), and physical activity (PA) [1–5]. These behaviors are distributed along an intensity continuum ranging from low energy expenditure, such as sleep, to vigorous PA requiring high energy expenditure [6, 7].

Currently, a wide variety of direct measurement instruments are used to assess physical behaviors of children, such as doubly labelled water, (in)direct calorimetry, polysomnography, direct (video) observation, and accelerometry [8]. Polysomnography is considered a “gold standard” for sleep, however, this can only be applied in a laboratory setting. Doubly labelled water is considered a “gold standard” for total energy expenditure, however, it cannot distinguish frequency, type, and intensity of specific physical behaviors [9, 10]. Direct calorimetry accurately measures metabolic rate in confinement, and indirect calorimetry allows for this assessment in free-living situations, however, both methods come with relatively high costs and are also not distinctive for frequency, type, and intensity of specific physical behaviors [11]. While direct (video) observation is considered a suitable comparator measure for assessing different types of physical behaviors, it is less suitable for assessing activity intensity because this can only be derived by assigning a metabolic equivalent to represent energy cost, which is unknown for the youngest age groups (i.e., infants and toddlers) [6, 7]. In addition, direct observation is very time consuming and requires trained observers scoring a specified protocol [9]. Given these limitations, these methods might not be feasible for measuring young children’s 24-h physical behaviors in free-living situations. Accelerometers can capture data on body movement, or lack thereof, continuously over extended periods of time, and are therefore widely considered the most promising method for physical behavior assessment.

Current reviews on reliability and validity of accelerometer-based methods for measuring physical behaviors in young children were limited to evaluation of only one measurement property [12] or one physical behavior [13]. Lynch and colleagues (2019) reviewed studies that evaluated criterion validity of accelerometers against indirect calorimetry, concluding that accelerometers can accurately assess SB and PA in children 3 to 18 years old [12]. De Vries and colleagues (2006) reviewed criterion-, convergent validity, test–retest- and inter-device reliability of accelerometers. They found that accelerometers provide reasonable estimates for assessing PA, however, no evidence on reliability was found in 2- to 4-years-old children [13]. Moreover, evidence on these measurement properties for infants (0—12 months) and toddlers (1—3 years) is lacking [14, 15]. Bruijns and colleagues (2020) reviewed estimates of PA and SB derived from accelerometer data in infants and toddlers and found that PA estimates were inconclusive and largely heterogeneous [14]. Additionally, no studies under three years old were found in a review on the evidence for methodological accelerometer decisions (e.g., epoch length, wear location, data analysis approach) for assessing PA in children aged 0—5 years [15].

While accelerometer-based methods provide reasonable estimates of time spent in SB, PA, and sleep in school-aged children [12, 13, 16–19], this cannot be generalized to young children due to major differences in types and intensity of their physical behaviors [20]. Physical activity types are different for children, depending on their developmental stage, e.g., daytime naps, crawling, and being carried in the youngest age groups [15, 21]. Moreover, the intensity of activities might differ between children depending on the efficiency of motor skills. For instance, toddlers start walking around one year of age, increase locomotor (e.g., running, jumping, hopping), stability (e.g., balancing, climbing), and develop object-control skills (e.g., kicking, catching, rolling) [22]. Preschoolers (3–5 years) further develop these skills and often participate in modified sports [23]. These differences in physical behaviors and motor development require age group specific studies on the validity and reliability of measurement instruments and analysis techniques, adapted to the child’s developmental stage.

For assessment of 24-h physical behavior in young children a complete overview of measurement properties of accelerometer-based methods is unavailable and there is no consensus about the optimal measurement protocol (e.g., epoch length, wear location) and accelerometer processing (e.g., cut-points, algorithms, machine learning methods) decisions for the use of accelerometer-based methods in young children [15]. To be able to select the most appropriate method for the child’s developmental stage, an overview of current accelerometer processing and study designs, and measurement properties of the available accelerometer-based methods is warranted. Therefore, we aimed to comprehensively review all studies examining the measurement properties test–retest, inter-device reliability, criterion- and convergent validity of accelerometer-based methods assessing 24-h physical behavior in young children aged 0–5 years, including an evaluation of the quality of evidence.

## Methods

We registered this review on PROSPERO (international prospective register of ongoing systematic reviews; registration number: CRD42020184751) and followed the Preferred Reporting Items for Systematic reviews and Meta-Analyses (PRISMA) guidelines [24].

### Search strategy

We systematically searched the electronic database PubMed (MEDLINE) up until 26^th^ June 2021. The search strategy focused on terms related to young children (e.g., infant, toddler, preschooler), accelerometer-based methods (e.g., accelerometry/methods, actigraphy), and measurement properties (e.g., validity, reliability). These terms were used in AND-combination with terms related to physical behavior: SB (e.g., inactive behavior, stationary behavior, sitting), PA (e.g., motor activity, tummy time, cycling), OR sleep (e.g., nap, bedtime, night rest). Articles related to animals, a variety of disorders (e.g., autism, attention deficit disorder), and diseases were excluded using the NOT-combination. Medical Subject Heading (MeSH), title and abstract (TIAB), and free-text search terms were used, and a variety of publication types were excluded (e.g., book sections, thesis). The full search strategy can be found in Additional File 1.

### Eligibility criteria

Studies were eligible for inclusion when the study: 1) used an accelerometer-based method to monitor at least one physical behavior: SB, PA, or sleep; 2) evaluated at least one measurement property of an accelerometer-based method: test–retest or inter-device reliability, criterion- or convergent validity; 3) included a (sub)sample of apparently healthy children, born term (> 37 weeks), with a mean age < 5 years or a wider range with the results for 0–5-year-olds reported separately; 4) was published in English in a peer-reviewed journal; and 5) full-text was available.

Studies were excluded when the study: 1) used a diary, parent- or proxy-report, or relied on parents for direct observation as comparison measure; 2) evaluated measurement properties in a clinical population, e.g., focused on only children with overweight or obesity; or 3) relied on accelerometer-based devices that do not directly output data on acceleration time series data or magnitude of acceleration, e.g., Fitbit.

### Selection procedures

We imported articles into reference manager software (EndNote X 9.1), and subsequently removed duplicate articles. Two researchers (AL and TA) independently screened titles and abstracts for eligibility using Rayyan and subsequently screened full-text articles. For four publications, the mean age of the study population was missing. To resolve this missing information, we contacted the authors. In addition, the reference lists of all relevant full-text articles were screened for possible inclusion of additional studies. A third researcher (MC) was consulted to resolve discrepancies.

### Data extraction

For all eligible studies, two researchers (AL and JA) extracted data using a structured form. Disagreements were resolved through discussion. Extracted data included the evaluated measurement properties, study and target population, accelerometer specifications (i.e., device and model, placement location and site, epoch length, axis), accelerometer data analysis approach used, outcome(s) and setting, comparison method (in case of validity), time interval (in case of test–retest reliability), and results of the evaluated measurement properties. The variety of accelerometer-based methods was described using code combinations of the following four elements: accelerometer brand, analysis approach, axis, and epoch length (see Additional File 3).

### Methodological quality assessment

Two researchers (AL and either TA or MC) rated the methodological quality of the included studies independently using a newly developed checklist to assess risk of bias. Risk of bias refers to whether the results for evaluating a measurement property are trustworthy based on the methodological study quality. In case of disagreement, all three researchers discussed the rating until consensus was reached.

#### Checklist development

We newly developed a Checklist for Assessing the Methodological Quality of studies using Accelerometer-based Methods (CAMQAM), as there was no standardized checklist available. The CAMQAM was inspired by quality assessment of patient reported outcomes, the standardized COnsensus-based Standards for the selection of health Measurement INstruments (COSMIN) risk of bias checklist [25–27], and by a previous review by Terwee and colleagues [28]. To fit accelerometer-based methods, we used the following relevant parts and, made minimal adjustments (e.g., wordings): ‘Box 6 Reliability’ for test–retest- and inter-device reliability,’Box 8 Criterion Validity’ for criterion validity, and ‘Box 9 Hypothesis testing for construct validity’ part ‘9a Comparison with other outcome measurement instruments’ for convergent validity. Moreover, we developed two boxes with additional items to rate methodological quality of studies assessing the criterion- or convergent validity of a specific accelerometer data analysis approach to categorize physical behavior: 1) conventional cut-points based method using a single value, or 2) multi-parameter method using more than one parameter, e.g., sleep algorithm, machine learning method. Two of the authors (TA and MC) independently rated the most diverse included studies for each measurement property. Thereafter, we added examples or explanations to clarify the items and ensure studies were scored using the correct box. The CAMQAM was used as a modular tool; only those boxes were completed for the measurement properties evaluated in the study.

For each examined measurement property, the study design requirements were rated as either very good, adequate, doubtful, or inadequate quality [25]. To rate the final methodological study quality, a worst score method was adopted, i.e., using the lowest rating of any item in a box. Additional File 2 presents the complete checklist and scoring manual.

In the appraisal of methodological study quality, the following study aspects were considered: study design (e.g., sample, epoch length, measurement duration, comparison instrument and their measurement properties in the study population) and the performed statistical analysis to evaluate the measurement property of the accelerometer-based method (see Table 1 in Additional File 2 for a summary of the definitions).

### Rating study results

The result of each study on a measurement property was rated against the criteria for good measurement properties proposed in the COSMIN guideline, i.e., sufficient (+), insufficient (-), inconsistent (±) or intermediate (?) [26]. Below is indicated for each measurement property which outcomes were considered sufficient (+). Outcomes were considered insufficient (-) when these criteria were not met, and intermediate (?) when not all necessary information was reported. Due to the great variety of accelerometer-based methods adopted in the studies, quantitative pooling or quantitatively summarizing of the results was not feasible.

#### Reliability

Reliability results were considered acceptable under the following conditions: 1) Intraclass Correlation Coefficients (*ICC*) or Kappa values (*κ*) were ≥ 0.70 [28]; or Pearson (*r*_*p*_), Spearman rank (*r*_*sp*_) or unknown (*r*) correlation coefficients were ≥ 0.80 [25]. Some studies reported multiple correlations per accelerometer-based method for reliability, e.g., separate correlations for different physical intensities. Therefore, we applied a rating per physical behavior (i.e., incorporating correlations separately for PA, SB, and/or sleep), and an overall rating (i.e., incorporating all correlations) to obtain final reliability rating for each study. When ≥ 75% of reliability outcomes were acceptable, a sufficient rating was received, when ≥ 50% and < 75% of reliability outcomes were acceptable an inconsistent rating was received, and an insufficient evidence rating was received when < 50% of reliability outcomes were acceptable.

#### Validity

Criterion validity was considered acceptable when: 1) correlations or *κ* with the ‘gold standard’ were ≥ 0.70 (Table 1, e.g., comparison measure was polysomnography for accelerometer-based methods aiming to assess sleep, or indirect calorimetry to score energy expenditure); or diagnostic test results (i.e., area under the receiver operating curve, accuracy, sensitivity, or specificity) were ≥ 0.80.

**Table 1:** Physical behavior assessed by accelerometer-based methods evaluating validity, subdivided by level of evidence^a^, and criteria for acceptable outcome values

**Physical behavior**	**Level of Evidence**		
	***Level 1***	***Level 2***	***Level 3***
Physical activity	Indirect calorimetry to score energy expenditure, e.g., DLW, AEE	Direct observation to score activity type	Pedometer (daily) step counts
	Direct video observation to score activity type	Accelerometer-based magnitude of acceleration (different brand/type)	
Sedentary behavior (primarily defined as an activity type, and secondarily as an intensity)	Direct video observation to score activity type	Direct observation to score activity type	Accelerometer-based magnitude of acceleration (different brand/type)
	Indirect calorimetry to score energy expenditure	Accelerometer-based orientation classification (thigh data), e.g., activPAL	
Sleep	Polysomnography	Videosomnography or direct video observation to classify sleep–wake states	Direct observation to classify sleep–wake states
		Indirect calorimetry, e.g., SMR	Accelerometer-based magnitude of acceleration (different brand/type)
**Outcome value**	**Acceptable results**		
	***Level 1***	***Level 2***	***Level 3***
*r*_*p*_*, r*_*sp*_*, r, R*^*2*^	≥ .70	≥ .60	≥ .60
*AUC-ROC, accuracy, Se, Sp*	≥ .80	≥ .80	≥ .80
*ICC, CCC κ, κ*_*w,*_* κ*_*qw*_	≥ .70	≥ .70	≥ .70

*Abbreviations*: AEE activity energy expenditure, *AUC-ROC* area under the receiver operating curve, *CCC* concordance correlation coefficient, *DLW* doubly labelled water, *ICC* intraclass correlation coefficient, *κ* Kappa, *κ*_*w*_ weighted Kappa, *κ*_*qw*_ quadratic weighted Kappa, *r*_*p*_ Pearson correlation coefficient, *r*_*sp*_ Spearman’s rank-order correlation coefficient, *r* unknown correlation coefficient, *R*^*2*^ R-squared value, *Se* sensitivity, *SMR* sleeping metabolic rate, *Sp* specificity

^a^ Level of evidence: level 1 indicating strong evidence, level 2 indicating moderate evidence, and level 3 indicating weak evidence

To rate the results of studies that evaluated convergent validity, we formulated criteria for acceptable results regarding the confidence in the comparison instrument to accurately measure the relevant physical behavior (i.e., level of evidence) (Table 1). We first assessed the level of evidence using these criteria, where level 1 indicated strong evidence, level 2 indicated moderate evidence, and level 3 indicated weak evidence. Thereafter, subdivided by the level of evidence, we rated study results as acceptable when: 1a) correlations (i.e., *r*_*p*_, *r*_*sp*_, *r*) with the comparison measure were ≥ 0.70 (level 1); or 1b) correlations with the comparison measure were ≥ 0.60 (level 2 or level 3) [13]; 1c) *ICC*, Concordance Correlation Coefficients (*CCC*), or *κ* with the comparison measure were ≥ 0.70; 2) or diagnostic test results were ≥ 0.80. As most studies reported multiple results, we applied a rating per physical behavior (i.e., incorporating results separately for SB, PA, and/or sleep), and an overall rating for each study. When ≥ 75% of the validity outcomes were rated as acceptable, a sufficient rating was received, when ≥ 50% and < 75% of validity outcomes were rated as acceptable an inconsistent rating was received, and an insufficient evidence rating was received when < 50% of validity outcomes were acceptable.

### Quality of evidence grading

Quality of evidence was graded using the Grading of Recommendations Assessment, Development and Evaluation (GRADE) approach as proposed in the COSMIN guideline, i.e., high, moderate, low, or very low [26], to indicate trustworthiness of the measurement property results. To derive the grading, the methodological study quality (i.e., risk of bias) was weighted with relevant risk factors: 1) inconsistency, i.e., unexplained inconsistency of results across studies, 2) imprecision, i.e., total sample size of the available studies, and 3) indirectness, i.e., evidence from different populations than the population of interest in this review [26]. The evidence grading was subsequently downgraded with one, two, or three levels for each risk factor, to moderate, low, or very low quality of evidence. The quality of evidence grading was performed for each measurement property and each accelerometer-based method separately.

## Results

**Fig. 1:**
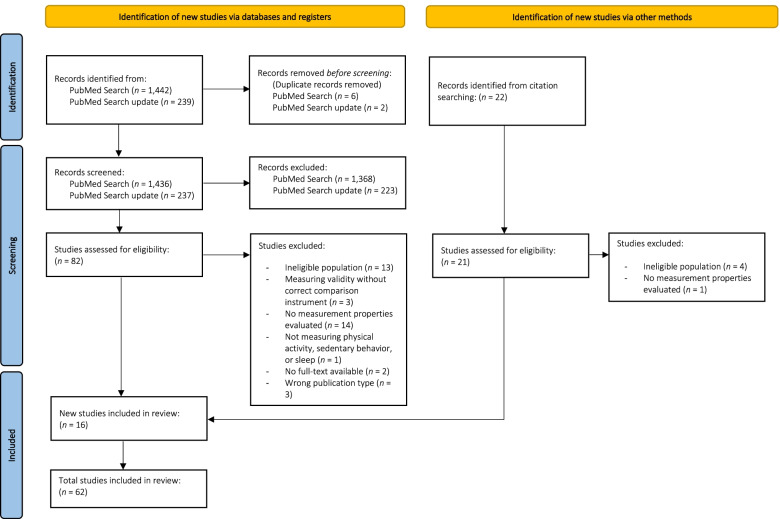
Preferred Reporting Items for Systematic reviews and Meta-Analyses (PRISMA) flow diagram of study inclusion

The systematic literature search yielded a total of 1,673 unique articles. After title and abstract screening, 82 full-texts were screened. Additionally, 16 articles were found through cross-reference searches. Therefore, a total of 98 full-text articles were assessed for eligibility, of which 62 were included (see Fig. 1 for the full selection process). Thirteen of the included studies evaluated the measurement properties of accelerometer-based methods for assessing SB, PA, and/or sleep in infants [29–41], nine in toddlers [42–50], and forty in preschoolers [51–90].

The included studies evaluated measurement properties for time series data or magnitude of acceleration directly [42, 45, 51–60, 62, 63, 70] or applied one of the following data analysis approaches to categorize physical behavior: a conventional cut-points based method using a single value [29–34, 42–47, 51–53, 56, 61, 64–81, 89], or a multi-parameter method (e.g., algorithm, machine learning) [30, 35–41, 48–50, 55, 66, 75, 82–90].

### Reliability

Table 2 summarizes the results for reliability, of which two studies examined test–retest reliability [29, 65], and one study examined inter-device reliability [42]. Test–retest reliability of an accelerometer-based method using a cut-points based method was evaluated by measuring SB and PA in preschool aged children [65]. Total PA, SB, light PA (LPA) and moderate-to-vigorous PA (MVPA) were considered reliable across all wear time criteria, except for absolute values of SB. For absolute values of SB, results were sufficient if data was collected for ≥ 5 days/week of ≥ 10 h. Despite adequate methodological study quality, these results received low quality of evidence as they were retrieved in a sample of 91 preschoolers. Inter-device reliability for epoch level activity counts (60 s) was rated as sufficient for activity counts in toddlers wearing two Actical devices side-by-side on the non-dominant ankle [42]. Despite adequate methodological study quality, these results received low quality of evidence as they were retrieved in a limited sample of 24 toddlers.

**Table 2:** Reliability of accelerometer-based methods, sorted by methodological study quality, study result rating and quality of evidence

Study	Study population^a^	Outcome(s) & Setting	Time interval	Methodological study quality^b^	Placement	Device-based method^c^	Results	Study result rating^d^	Quality of evidence^e^
Hager et al. (2016) [42]	Toddlers *n* = 24 * 2 Ac *Age* = 24.5 ± 5.2 (14.7 to 35.5) months *Sex* = 41.7% girls	Activity counts Laboratory (structured activities)	n.a	A	Non-dominant ankle; side-by-side	**Ac-cts-omni-60**	*ICC*_*?*_ = .98	+	Low
Aadland & Johannessen (2015) [65]	Preschoolers *n* = 91 *Age* = 4.07 ± 0.5 (3 to 5) years *Sex* = 51% girls	Total PA, SB, LPA and MVPA in min/day and % of valid wear time Free-living	14 days	A	Not reported	**AG-Ev-VA-10**	*ICC*^*−*^_*(1,1)*_ ≥ .75 for all outcomes across all wear criteria (≥ 6, 8 and 10 h/day and ≥ 3 and 5 days/week) Except for SB (min/day): ICC^−^_(1,1)_ .61 to .81 Higher *ICC*^*−*^_*(1,1)*_ for percentages than absolute values *ICC*^*−*^_*(1,1)*_ ≥ .80 (≥ 6 and ≥ 8 h/day) > 7 days required *ICC*^*−*^_*(1,1)*_ ≥ .80 (≥ 10 h/day) within 7 days: 3.9 to 7.1 days	+ (SB + ; PA +)	Low
Greenspan, Cunha, & Lobo (2021) [29]	Infants *n* = 16 *Age* = 3.1 ± 1.1 (1.8 to 5) months *Sex* = 51% girls	Body position: supine, reclined, upright, inclined, and prone Free-living (structured play)	0 days	I	Trunk	**GG-Gr-HA/VA/DA-1**	*κ*_*w*_ = .89, 95% *CI* (.87 to .91) Supine: agreement = 89.2% Reclined: agreement = 72.6% Upright: agreement = 90.9% Inclined: agreement = 84.6% Prone: agreement = 91.1%	+	Very low

*Abbreviations: Ac* Actical, *AG* ActiGraph, *cpm* counts per minute, *cts* counts, *DA* diagonal axis (z-axis), *Ev* Evenson’s cut-points (2008) [91], *GG* get around garment with ADXL335, *Gr* Greenspan’s cut-points (2021) [29], *HA* horizontal axis (x-axis), *ICC*_*?*_ intraclass correlation coefficient analysis decision was unclear, *ICC*^*−*^_*(1,1)*_ intraclass correlation coefficient inappropriate analysis decision one-way random effects model,* κ*_*w*_weighted Kappa, *LPA* light physical activity, *MVPA* moderate-to-vigorous physical activity, *n.a.* not applicable, *omni* omnidirectional, *PA* physical activity, *r* unknown correlation coefficient, *SB* sedentary behavior, *VA* vertical axis (y-axis), *1* 1 s epoch, *10* 10 s epoch, *60* 60 s epoch

^a^Age presented as mean ± SD (range)

^b^Methodological study quality based on newly developed checklist: VG very good, A adequate, D doubtful, I inadequate

^**c**^Device-based method described using code combinations of four elements resulting in the following format: brand-axis-approach-epoch length

^**d**^Study result rating based on COSMIN guideline: + sufficient, ± inconsistent, - insufficient, ? intermediate

^**e**^Quality of evidence based on GRADE approach

### Validity

The following subsections present the results for validity by age group. Notably, most studies used the vertical axis (VA). In studies among infants the accelerometer was predominantly worn on the ankle, while for studies among toddlers or preschoolers the devices were mainly placed on the hip. Unless otherwise specified, we report study results based on this majority placement and axis.

#### Infants

Table 3 summarizes the results for validity, of which four studies examined criterion validity [30–32, 35], and nine studies examined convergent validity [29, 33, 34, 36–41] in infants.

**Table 3:** Criterion and convergent validity of accelerometer-based methods for infants, sorted by methodological study quality, quality of evidence (level of evidence), and study result rating

	**Study**	**Study population**^**a**^	**Outcome(s) & Setting**	**Comparison measure(s)**	**Methodological study quality**^**b**^	**Placement**	**Device-based method**^**c**^	**Results**	**Study result rating**^**d**^	**Quality of evidence**^**e**^** (level of evidence)**
Criterion validity	Galland et al. (2012) [30]	*n *= 31 *Age *= 13.0 ± 3.1 (10.0 to 22.3) weeks *Sex* = 27.3% girls	Sleep quality: sleep latency, TST, sleep efficiency, and WASO (duration and number) Laboratory (daytime nap)	Polysomnography to score sleep stages as sleep (active, quiet, intermediate) and wake	VG	Shin	**Ac-CS-omni-15**	*accuracy* = 86.3%, *Se* = 85.7%, *Sp* = 84.3%, *κ* = .66, *PABAK* = .72; Sleep latency *r*_*p*_ = .79*** (underestimated by 3 min*), TST *r*_*p*_ = .83***, Sleep efficiency *r*_*p*_ = .87***, WASO (duration) *r*_*p*_ = .48** (overestimated by 6.5 min***), WASO (number) *r*_*p*_ = .35 (overestimated by 3***)	+	Low
							**Ac-CS-omni-30**	*accuracy* = 86.1%, *Se* = 84.7%, *Sp* = 87.4%, *κ* = .66, *PABAK* = .72	+	
							Ac-CS-omni-60	*accuracy* = 84.5%, *Se* = 79.8%, *Sp* = 90.2%, *κ* = .63, *PABAK* = .69	-	
							Ac-S0-omni-15	*accuracy* = 82.3%, *Se* = 94.8%, *Sp* = 57.8%, *κ *= .55, *PABAK* = .65; Sleep latency *r*_*p*_ = .80*** (underestimated by 5 min***), TST *r*_*p*_ = .76*** (overestimated by 11 min*), Sleep efficiency *r*_*p*_ = .76*** (overestimated by 16.5%***), WASO (duration) *r*_*p*_ = .48** (overestimated by 7 min***), WASO (number) *r*_*p*_ = .38* (overestimated by 3***)	±	
							Ac-S0-omni-30	*accuracy* = 86.3%, *Se* = 91.0%, *Sp* = 77.7%, *κ *= .65, *PABAK* = .72	±	
							Ac-S0-omni-60	*accuracy* = 84.5%, *Se *= 80.0%, *Sp* = 89.9%, *κ* = .63, *PABAK* = .69	±	
							Ac-PS-omni-15	*accuracy* = 84.3%, *Se* = 95.1%, *Sp* = 63.1%, *κ* = .59, *PABAK* = .69; Sleep latency *r*_*p*_ = .93***, TST *r*_*p*_ = .80*** (overestimated by 11 min*), Sleep efficiency *r*_*p*_ = .81*** (overestimated by 13.6%*), WASO (duration) *r*_*p*_ = .50** (overestimated by 3.5 min***), WASO (number) *r*_*p*_ = .13 (overestimated by 3***)	±	
							Ac-PS-omni-30	*accuracy* = 86.8%, *Se* = 90.8%, *Sp* = 79.0%,* κ* = .66, *PABA*K = .74	±	
							Ac-PS-omni-60	*accuracy* = 84.6%, *Se* = 79.9%, *Sp* = 90.3%, *κ* = .63, *PABAK* = .69	±	
							Ac-WSC-omni-15	*accuracy* = 83.2%, *Se* = 94.6%, *Sp* = 60.2%, *κ* = .57, *PABAK* = .66; Sleep latency *r*_*p*_ = .78*** (underestimated by 4 min*), TST *r*_*p*_ = .78*** (overestimated by 10 min*), Sleep efficiency *r*_*p*_ = .79*** (overestimated by 15.2%*), WASO (duration) *r*_*p*_ = .41* (overestimated by 7 min***), WASO (number) *r*_*p*_ = .29 (overestimated by 3***)	±	
							Ac-WSC-omni-30	*accuracy* = 86.5%, *Se* = 90.9%, *Sp* = 78.2%, *κ* = .66, *PABAK* = .73	±	
							Ac-WSC-omni-60	*accuracy* = 84.5%, *Se* = 80.0%, *Sp* = 89.9%, *κ *= .63, *PABAK* = .69	±	
	Insana, Gozal, & Montgomery-Downs (2010) [31]	*n* = 22 *Age* = 14.1 ± 0.6 (13.0 to 15.0) months *Sex* = 45.5% girls	Sleep quality: TST and WASO (duration) Laboratory (in the hospital)	Polysomnography to score sleep stages as sleep and wake	VG	Ankle	Aw-ACT40-uni-15	*accuracy *= 89.6%, *Se* (89.0 to 96.3%), *Sp* = 58.9% TST: *r*_*p*_ = .83***, *ICC* = .80, *MD* = -72.3 (*SD* = 61.5) min***, *d* = .70 WASO: *r*_*p*_ = .52*, *ICC* = .65, *MD* = 13.9 (*SD* = 30.9) min*, *d* = .44	±	Low
							Aw-ACT80-uni-15	TST: *r*_*p*_ = .84***, *ICC* = .86, *MD* = 52.1 (*SD* = 60.4) min**, *d* = .50 WASO: *r*_*p*_ = .52*, *ICC* = .65, *MD* = 6.4 (*SD* = 26.8) min	±	
	Rioualen et al. (2015) [32]	*n* = 24 *Age* = 2.5 ± 0.7 days *Sex* = 50.0% girls	Sleep stage: wake, active sleep, and quiet sleep Laboratory (in the hospital)	Polysomnography to score sleep stages as sleep (active, quiet) and wake	VG	Wrist	Aw-ASA40-uni-60	*Se* = 93%, 95% *CI* (89 to 96) *Sp* = 20%, 95% *CI* (14 to 27)	±	Low
						Ankle	Aw-ASA40-uni-60	*accuracy* = 58.5%, *Se* = 87%, 95% *CI *(81 to 93), *Sp* = 31%, 95% *CI* (24 to 39)	-	
	Lewicke, Sazonov, & Schuckers (2004) [35]	*n* = 25 *Age* = n.r *Sex* = n.r	Sleep stage: wake, active sleep, and quiet sleep Laboratory	Polysomnography to score sleep stages as sleep (active, quiet, indeterminate) and wake	I	Left hip	mSCA-LVQ-uni-30	Training: *accuracy* = 80.7%, *Se* = 94.6%, *Sp* = 48.2% Validation: *accuracy* = 75.3%, *Se* = 92.3%, *Sp* = 42.4%	±	Very low
Convergent validity	Camerota et al. (2018) [33]	*n* = 82 *Age *= 3.6 ± 0.6 (2.7 to 5.2) months *Sex* = 43% girls	Sleep quality: sleep onset time, rise time, sleep period, WASO (number and duration), TST, and longest sleep period Free-living (at home)	Videosomnography to score sleep stages as sleep and wake	VG	Left ankle	Aw-ACTdef-uni-15	*κ *= .47, *Se* = 52%, *Sp* = 95% Sleep onset time: *r* = .79*, underestimated by 29 min***, *d* = .43 Rise time: *r* = .76* Sleep period: *r* = .78*, overestimated by 23.6 min*, *d* = .28 WASO: (number) *r* = .47*, underestimated by 1.1***, *d* = .54; (duration): *r* = .59*, overestimated by 58.4 min***, *d* = 72 TST: *r* = .54* Longest sleep period: *r* = .38*, overestimated by 35.5 min*, *d* = .28	-	Moderate (level 2)
	Greenspan et al. (2021) [29]	*n* = 16 *Age* = 3.1 ± 1.1 (1.8 to 5) months *Sex* = 51% girls	Body position: supine (parallel to floor stomach facing up), reclined (trunk tilted about 45° posteriorly from upright), upright (90°), inclined (trunk tilted about 45° anteriorly from upright), and prone (parallel to floor stomach facing down) Free-living (structured and free play)	DVO to score body position by two independent raters using outlined definitions	VG	Trunk	GG-Gr-HA/VA/DA-1	*κ*_*w*_ = .84, 95% *CI* (.83 to .84) Supine: *accuracy* = 90.7%, *r*_*sp*_ = .97*** Reclined: *accuracy* = 73.5%, *r*_*sp*_ = .89*** Upright: *accuracy* = 83.1%, *r*_*sp*_ = .97*** Inclined: *accuracy* = 41.4%, *r*_*sp*_ = .67** Prone: *accuracy* = 78.4%, *r*_*sp*_ = .96***	±	Low (level 1)
	Jun & Choi (2020) [40]	*n* = 9 *Age* = 144 ± 284.5 (2 to 720) days *Sex* = 59.1% girls	Activity type: sleeping (without substantial movement), strong movement (struggling with crying or in agony), weak movement (moving in comfy state), and movement by external force (from nurse or caregiver) Laboratory (at the hospital)	DVO to score activity type by two independent raters using a 3-stage classification scheme	A	Chest	**ICM-DNN-HA/VA/DA-4**	*accuracy* = .96, *F-score* = .95, *precision* = .98, *recall* = .93, *Sp* = .98 Sleep: *accuracy* = .97, *F-score* = .99, *precision* = .99, *recall* = 1.0, *Sp* = .94 Strong movement:* accuracy* = .97, *F-score* = .95, *precision* = .96, *recall* = .94, *Sp* = .99 Weak movement:* accuracy *= .95, *F-score* = .93, *precision* = .97, *recall* = .90, *Sp* = .99 External force: *accuracy* = .95, *F-score* = .95, *precision* = 1.0, *recall* = .89, *Sp* = 1.0	+	Very low (level 1)
	Airaksinen et al. (2020) [39]	*n* = 22 *Age* = 6.7 ± 0.8 (4.5 to 7.7) months *Sex* = 59.1% girls	Body position: prone (navel on floor), supine (lower back on floor), side left/right, crawl posture (supported by hands, and knees or feet); Movement: macro still (no movement), turn left/right (change of posture along prone-side-supine axis), pivot left/right (change of facing whole body direction, without movement), crawl proto (practice crawl without moving during prone/side posture and move multiple limbs during supine posture), crawl commando (crawl with forward movement) Laboratory (semi-structured)	DVO to score body position and movement by three independent raters using a developed annotation scheme	A	Arms + legs; proximally	**SM-CNN-VM-2**	Movement track: *F-score* = 80% Full agreement: posture: *accuracy* = 99.1%, movement: *accuracy* = 90.7%; All frames: posture: *accuracy* = 98.2%, movement: *accuracy* = 81.7% Most frequent confusions in prone-side-supine axis as well as between crawl posture and prone Individual sensors lower performance (left arm posture: *accuracy* = 71%, movement: *accuracy* = 70%, left leg posture: *accuracy* = 90%, movement: *accuracy* = 68%) compared to four-sensor setup (posture: *accuracy* = 95%, movement: *accuracy* = 80%), and two-sensor setup (right leg + arm posture: *accuracy* = 94%, movement: *accuracy* = 78%; left leg + arm posture: *accuracy* = 95%, movement: *accuracy* = 79%; left + right arm posture: *accuracy* = 85%, movement: *accuracy* = 72%; left + right leg posture: *accuracy* = 93%, movement: *accuracy* = 72%)	+ Arm: - Arms: ± Leg: ± Legs: ± Combined 2-sensor: ± ; 4-sensor: +	Very low (level 1)
							SM-SVM-VM-2	Movement track: *F-score* consistently 5–10% lower performance in prone positions (crawl proto*, turn right***; pivot left*) Posture classification comparable between CNN and SVM	±	
	Smith et al. (2015) [41]	*n* = 12 *Age* = 6.8 ± 2.9 (1 to 12) months *Sex* = 66.7% girls	Activity type: leg movement (change of limb position) Laboratory	DVO to score activity type by one rater	D	Legs	**Op-AAV-VM-n.r**	*accuracy* = 92.7%, *Se* = 92.0%	+	Very low (level 1)
	Hewitt et al. (2019) [34]	*n* = 32 *Age* = 15.2 ± 6.4 (4.7 to 24.9) weeks *Sex* = 40.6% girls	Body position: prone on floor (tummy with both hips touching the floor), prone supported (held in prone, on parent’s chest), and non-prone (supine, left/right side lying, reclined in car seat/pram, upright, supported sitting, cradle hold) Laboratory (structured positions)	DVO to score body position by one rater (one randomly chosen video was scored by four independent raters) using outlined definitions	D	Right hip	AG-He-VA/HA-1	Prone: *accuracy* = 90%, *MD* = -18.3 s, LoA (-97.0 to 60.3) Non-prone: *accuracy* = 99.9%, *MD* = -0.2 s, *LoA* (-1.2 to 0.9) Prone supported: *accuracy* = 63.6%, *MD* = -127.3 s, *LoA* (-324.7 to 70.2)	±	Very low (level 1)
							GA-Ai-VA/HA-1	Prone: *accuracy* = 95.4%, *MD* = -8.4 s, *LoA* (-78.2—61.3) Non-prone: *accuracy* = 98%, *MD* = 31.2 s, *LoA* (-154.9 to 92.4) Prone supported: *accuracy* = 52.2%, *MD* = -166.9 s, *LoA* (-390.7 to 56.8)	±	
						Ankle	AG-He-VA/DA-1	Prone: *accuracy* = 87.9%, *MD* = -22.1 s, *LoA* (-124.0 to 79.7) Non-prone: *accuracy* = 96.3%, *MD* = -56.6 s, *LoA* (-209.5 to 96.3) Prone supported: *accuracy* = 53.3%, *MD *= -163.1 s,* LoA* (-431.7 to 105.6)	±	
						Chest	MB-He-VA/HA/DA-1	Prone: *accuracy* = 79.2%, *MD* = -38 s, *LoA* (-194.5 to 118.5) Non-prone: *accuracy* = 99.9%, *MD* = 2.0 s, *LoA* (-14.0 to 10.8) Prone supported: *accuracy* = 66.1%, *MD* = -113 s, *LoA* (-355.6 to 129.6)	-	
	Horger et al. (2021) [37]	*n* = 9 *Age*_*start*_ = 7.96 ± 2.51 (0.89 to 8.19) months *Age*_*end*_ = 10.89 ± 1.14 (9.5 to 13.08) months *Sex* = 44.0% girls	Sleep quality: sleep onset, morning wake time, WASO, sleep efficiency, and sleep duration Free-living (at home)	Videosomnography to score sleep quality	D	Left ankle	MM-PSinf-uni-60	Sleep onset: *r*_*p*_= .94**, *MD* = 0.76 min, *SD* = 34.5 min Morning wake: *r*_*p*_= .74, *MD* = -14.1 min,* SD* = 40.4 min WASO: *r*_*p*_= .20, *MD* = 1.2, *SD* = 3.9 Sleep efficiency: *r*_*p*_= .30 Sleep duration: *r*_*p*_= .36	-	Very low (level 2)
							MM-PS-uni-60	Sleep onset: *r*_*p*_= .86** Morning wake: *r*_*p*_= .55 WASO: *r*_*p*_= .27 Sleep efficiency: *r*_*p*_= .51 Sleep duration: *r*_*p*_= .12	-	
	Sadeh et al. (1995) [38]	*n* = 41 (10 newborns; 11 3-month-olds; 10 6-month-olds; 10 12-month-olds) *Age* = 0 to 12 months *Sex* = 46.4% girls	Sleep stage: sleep (active and quiet) and wake Free-living (newborns at hospital; others at home)	DO using Thoman’s observation scheme (1975, 1995) to score sleep stage as sleep (active and quiet), wake, sleep–wake transition and uncertain	D	Left ankle	**AMA-PS-uni-60**	All infants: *accuracy* = 83.4% (sleep–wake = 95.6%; wake = 93.5%; active sleep = 74.9% and quiet sleep = 78.0%) (excl. 12-month-olds) Newborns: *accuracy* = 74.9% (wake = 82.8%; active sleep = 74.4.1%, quiet sleep = 54.9%) 3-month-olds: *accuracy* = 87.3% (wake = 92.5%, active sleep = 78.3%, quiet sleep = 87.2%; sleep–wake = 93.8%); 6-month-olds: *accuracy* = 83.2% (wake = 97.8%, active sleep = 66.4%, quiet sleep = 76.7%; sleep–wake = 97.9%); 12-month-olds: wake = 99.3%; sleep–wake = 97.2% Wake time: *r* (.85 to .99), % active sleep time: *r* (.78 to .98), % quiet sleep time: *r* (.36 to .85) (lowest correlations in newborns) Wake: *MD* (0.2 to 3.5%), sleep: *MD* (0.4 to 11.3%)	± < 3 months: - > 3 months: +	Very low (level 3)
	Gnidovec, Neubauer, & Zidar (2002) [36]	*n* = 10 *Age* = 1 to 6 months *Sex* = 40% girls	Sleep stage: Sleep (active and quiet) and wake (active and quiet) Free-living (at home)	DO using Thoman’s observation scheme (1990) to score sleep stage as sleep (active and quiet), wake (active and quiet), sleep–wake transition and uncertain	I	Left ankle	**Gw-ASW-uni-10**	1-month: *accuracy* = 72.0% (sleep = 88.7%, wake = 57.5%); 3-months: *accuracy* = 90.1% (sleep = 97.0%, wake = 84.5%); 6-months: *accuracy* = 95.0% (sleep = 88.4%, wake = 98.0%) After 3^rd^ month 88.9% and 6^th^ month 94.4% *accuracy* (calibration sample n = 5) Invalid discrimination between active- and quiet sleep all age groups < 75%, best for 3-month-olds (*accuracy* = 74.3%; active sleep: *accuracy* = 49.3%, quiet sleep: *accuracy* = 88.3%), as compared to 1-month (*accuracy* = 58.5%; active sleep: *accuracy *= 35.9%, quiet sleep: *accuracy* = 90.5%) and 6-months (*accuracy* = 66.5%; active sleep: *accuracy* = 39.9%, quiet sleep: *accuracy* = 87.1%)	± 1 month: - > 3 months: +	Very low (level 3)

*Abbreviations:* *AAV* acceleration and angular velocity algorithm [41], *Ac* Actical, *ACTdef* Actiware software with default wake threshold value of .888 * mean acceleration, *ACT40* Actiware software with wake threshold value of 40, *ACT80* Actiware software with wake threshold value of 80, *ASA40* Actiwatch activity and sleep analysis software with wake threshold value = 40, *AG* ActiGraph, *Ai* Activinsights software, *AMA* AMA-32, *ASW* automatic sleep wake scoring algorithm, *Aw* Actiwatch, *CI* confidence interval, *CNN* convolutional neural network, *CS* count-scaled algorithm [30], *d* Cohen’s d, *DA* diagonal axis (z-axis), *DNN* deep neural network, *DO* direct observation, *DVO* direct video observation, *GA* GENEActiv, *GG* get around garment with ADXL335, *Gr* Greenspan’s cut-points (2021) [29], *Gw* Gaewiler, *HA* horizontal axis (x-axis), *He* Hewitt’s cut-points (2019) [34], *ICC* intraclass correlation coefficient, *ICM* ICM20600 chip, *κ* Kappa, *κ*_*w*_ weighted Kappa, *LoA* limits of agreement, *LVQ* neural network learning vector quantization, *MB* MonBaby, *MD* mean difference,*MM* MicroMini sleep watch, *mSCA* miniature semiconductor chip accelerometer, *n.r.* not reported, *omni* omnidirectional, *Op* Opal APDM, *PABAK* prevalence- and bias-adjusted Kappa, *PS* probability scaled algorithm [38], *PSinf* probability scaled algorithm for infants [92], *r* correlation coefficient (unknown), *r*_*p*_ correlation coefficient (Pearson), *r*_*sp*_ correlation coefficient (Spearman rank), *SD* standard deviation, *Se* sensitivity, *SM* Suunto Movesense sensor, *Sp* specificity, *SVM* support vector machine, *S0* zero-threshold computation [93], *TST* total sleep time, *uni* uniaxial (axis was not specified)*,* *VA* vertical axis (y-axis), *VM* vector magnitude, *WASO* wake after sleep onset, *WSC* weighted sum activity algorithm [30], *1* 1 s epoch, *2* 2 s epoch, *4* 4 s epoch, *10* 10 s epoch, *15* 15 s epoch, *30* 30 s epoch,  *60* 60 s epoch

^**a**^Age presented as mean ± SD (range)

^**b**^Methodological study quality based on newly developed checklist: VG very good, A adequate, D doubtful, I inadequate

^**c**^Device-based method described using code combinations of four elements resulting in the following format: brand-axis-approach-epoch length

^**d**^Study result rating based on COSMIN guideline: + sufficient, ± inconsistent, - insufficient, ? intermediate

^**e**^Quality of evidence based on GRADE approach

^*^p < *.05*

^**^p < *.01*

^***^p < *.001*

No studies assessed validity of cut-points for SB, LPA and MVPA in infants, while for sleep no cut-points (i.e., wake thresholds) were evaluated as valid [30–33]. Quality of evidence was low for studies evaluating criterion validity, as results were retrieved in limited samples of 22 to 31 infants, despite very good methodological quality [30–32]. The results of the study that evaluated convergent validity, were insufficient, despite moderate quality of evidence [33].

In contrast, multi-parameter methods were more suitable for assessing the physical behavior of infants than a conventional cut-points based method. Infant leg movements could sufficiently be distinguished from non-infant produced movement using an algorithm describing velocity and acceleration magnitude for this activity [41]. However, these results received very low quality of evidence as methodological study quality was doubtful and the results were retrieved in a sample of only 12 infants.

For posture and movement classification, using arm and leg data, validity of convolutional neural networks was rated as sufficient [39]. The performance of convolutional neural networks and supported vector machines were comparable for classification of infant specific postures, e.g., tummy time and crawl posture. However, for movement in prone positions (e.g., crawl, turn and pivot) the performance of the convolutional neural networks was consistently 5 to 10% higher than the performance of support vector machines, resulting in a sufficient study result rating for the former and an inconsistent rating for the latter. Despite adequate methodological study quality, these results received low quality of evidence as the results were retrieved in a sample of 22 infants. Another neural network using chest data was rated as sufficient for sleep and movement classification [40]. Despite adequate methodological study quality, the results received very low quality of evidence as the results were retrieved in a sample of only nine infants.

Sleep could be distinguished from wake from 3-months of age using different multi-parameter methods [30, 36, 38]. Convergent validity of the Sadeh algorithm that calculates the probability of sleep, was rated as sufficient in free-living situations for infants [38]. However, it was less suitable to distinguish active and quiet sleep. These results received very low quality of evidence as the results were retrieved in a limited sample of 41 infants and methodological study quality was doubtful. Similarly, convergent validity of the automatic sleep–wake scoring algorithm developed for raw data was rated as sufficient to distinguish sleep from wake, despite low accuracy for distinguishing active from quiet sleep [36]. However, these results received very low quality of evidence as they were retrieved in a sample of only 10 infants and methodological study quality was inadequate. Galland and colleagues (2012) determined the accuracy of three algorithms for distinguishing sleep from wake states using 15-, 30- and 60 s epochs in infants with a mean age around 3-months [30]. In line with previous results, criterion validity of the Sadeh and the Cole-Kripke (computing the weighted sum activity) algorithm was rated as insufficient. However, criterion validity of an algorithm similar to the Cole-Kripke algorithm that uses count-scaled data (leg placement) was rated as sufficient in infants of around 3-months of age using 15 s or 30 s epochs. The best performing algorithm used a sampling epoch of 15 s. Sleep agreement of the other algorithms was highest using the 15- or 30 s epoch, however, at the expense of wake agreement. Notably, correspondence with polysomnography was poorest for the number of wake time after sleep onset using 60 s epochs. Despite very good methodological study quality, these results received low quality of evidence due to the limited sample size of 31 infants.

#### Toddlers

Table 4 summarizes the results of nine studies in toddlers that examined convergent validity [42–50]. No studies evaluated methods to distinguish sleep from wake.

**Table 4:** Convergent validity of accelerometer-based methods for toddlers, sorted by methodological study quality, quality of evidence (level of evidence), and study result rating

	**Study**	**Study population**^**a**^	**Outcome(s) & Setting**	**Comparison measure(s)**	**Method** **ological study quality**^**b**^	**Placement**	**Device-based method**^**c**^	**Results**	**Study result rating**^**d**^	**Quality of evidence**^**e**^ **(level of evidence)**
Convergent validity	Kwon et al. (2019) [49]	*n* = 21 *Age* = 25 ± 2.5 (13 to 35) months *Sex* = 50% girls	Activity type: running (forward from one place to another), walking (forward from one place to another), climbing up/down (the stairs/foam climber), crawling (moving forward on two hands and two knees), riding a ride on toy (sitting on toy, moving forward using two feet), standing (without lifting a foot), sitting (on the ground), stroller (sit on stroller/wagon pushed by adult), and being carried (by adult while adult is walking) Free-living (free play in commercial indoor playroom)	DVO using a developed coding scheme to score activity type by two independent raters	VG	Hip	**AG-RF-VA/HA/DA/VM-5**	Best differentiating features: DA FFT SD (*d'* = 0.64), DA FFT max (*d' *= 0.61), HA FFT SD (*d'* = 0.47). Feature importance all 78 features < 0.1 (highest: SD of VM 0.039). Top 10 ranked features: basic quantiles (e.g., min, median, max) of single axis direct values and FFT values RF: *accuracy* = 89%, *precision* = 88%, *recall* = 89%, *F-score* = .88 58% of carried labels were correctly (SB) classified, whereas 89% of ambulation was correctly classified	+	Low (level 1)
	Costa et al. (2014) [46]	*n* = 20 *Age* = 2.99 ± 0.48 (2 to 3) years *Sex* = 60% girls	Activity intensity: Total time in SB, LPA, and MVPA Laboratory (semi-structured activity sessions)	DVO using CARS (modified) to score activity type and determine activity intensity (SB: stationary with no movement, and with movement of the limbs; LPA: slow/easy translocation; MVPA: translocation fast, and with moderate effort) by one rater (repeated after one month for random minute of each child)	VG	Right hip	AG-C-VM-5	Total PA: *CCC* = .80, *MD* = 48.0 s*, *LoA *(-217.9 to 121.9) SB: *CCC* = .74, *MD* = 48.0 s*, *LoA* (-121.9 to 217.9) LPA: *CCC* = .34, *MD* = -163.5 s***, *LoA* (-343.0 to 16.0) MVPA: *CCC* = .40, *MD* = 117.8 s***, *LoA* (-51.4 to 286.9)	± (SB + ; PA -)	Low (level 1)
							**AG-C-VA-5**	Total PA: *CCC* = .84, *MD* = 5.3 s,* LoA* (-171.1 to 181.6) SB: *CCC* = .77, *MD* = -5.3 s, *LoA* (-181.6 to 171.1) LPA: *CCC* = .45***, *MD* = -112.5 s, *LoA* (-320.5 to 95.5) MVPA: *CCC *= .42, *MD* = 115.5 s***, *LoA* (-49.3 to 280.3)	± (SB + ; PA -)	
							AG-Pa-VA-15	Total PA: *CCC *= .71, *MD* = 50.5 s,* LoA* (-183.0 to 284.0) SB: *CCC *= .75, *MD* = -22.0 s, *LoA* (-209.7 to 165.7) LPA: *CCC* = .38, *MD* = -115.3 s**, *LoA* (-397.27 to 166.8) MVPA: *CCC* = .30, *MD* = 165.8 s***, *LoA* (-71.2 to 402.73)	± (SB + ; PA -)	
							**AG-T12-VA-15**	Total PA: *CCC* = .85,* MD* = 15.3 s, *LoA* (-152.4 to 182.9) SB: *CCC* = .79, *MD *= -15.3 s, *LoA* (-182.9 to 152.4) LPA: *CCC* = .36, *MD* = -150.5 s***, *LoA *(-391.1 to 90.1) MVPA: *CCC* = .30, *MD* = 165.8 s***, *LoA *(-71.2 to 402.7)	± (SB + ; PA -)	
	van Cauwenberghe et al. (2011) [45]	*n* = 31 *Age* = 20 ± 4 (12 to 30) months *Sex* = 45.2% girls	Activity intensity: SB, LPA and MVPA Free-living (out- & indoor free play at childcare)	DVO using OSRAC-P to score activity type and derive activity intensity (SB: stationary and motionless, and stationary with limb/trunk movement; LPA: slow, easy movement; MVPA: moderate, and fast movement) by two independent raters	VG	Right hip	AG-cts-VA-15	mean *r*_*sp*_ = .66*** epoch-by-epoch intensity level *r*_*sp*_ = .52***	-	Low (level 1)
							AG-Pa-VA-15	*accuracy* = 58.3% SB: *AUC-ROC *= .71, *Se* = 67.0%, *Sp* = 75.4% LPA: *AUC-ROC* = .62, *Se* = 60.0%, *Sp* = 63.2% MVPA: *AUC-ROC* = .57, *Se* = 21.5%, *Sp* = 91.3%	- (SB -; PA -)	
							AG-Si3-VA-15	*accuracy* = 52.7% SB: *AUC-ROC* = .58, *Se* = 91.8%, *Sp* = 23.9% LPA: *AUC-ROC* = .52, *Se* = 14.6%, *Sp* = 89.0% MVPA: *AUC-ROC* = .53, *Se* = 8.9%, *Sp* = 97.1%	- (SB -; PA -)	
							AG-vC-VA-15	*accuracy* = 52.2% SB: *AUC-ROC* = .56, *Se* = 94.4%, *Sp *= 17.2% LPA: *AUC-ROC* = .51, *Se* = 9.0%, *Sp* = 93.7% MVPA: *AUC-ROC* = .53, *Se* = 10.0%, *Sp* = 96.9%	- (SB -; PA -)	
	Trost et al. (2012) [44]	*n* = 18 *Age* = 2.3 ± 0.4 years *Sex* = 55.6% girls	Activity intensity: SB, LPA and MVPA Free-living (regularly scheduled play)	DVO using CARS (modified) to score activity type and determine activity intensity (SB: lying down or sitting; LPA: standing; MVPA: walking and running) by two raters	VG	Right hip	AG-T12-VA-15	SB: *MD* = -7.6***, *LoA* (-17.6to 2.3) LPA: *MD* = 7.2***, *LoA* (-2.0 to 16.3) MVPA: *MD *= 0.5, LoA (-2.6 to 3.5)	?	Low (level 1)
				Pre-school cut-points: AG-Si/vC/Re/Pa/N-VA-15	VG			Preschool and toddler cut-points overestimated SB*** and underestimated LPA***; Si** and vC ** underestimated MVPA; Pa (≥ 420) same trend as the toddler cut-point T12: *MD* = 0.5, 95% *LoA* (-2.5 to 3.5)	?	Low (level 2)
	Albert et al. (2020) [48]	*n* = 22 *Age* = 1.5 ± 0.5 (1.1 to 2) years *Sex* = 54.6% girls	Activity type: Run/walk, crawl, climb, stand, sit, lie down, carried, and stroller as defined by Kwon et al. (2019)[49] Free-living (guided play)	DVO using a developed coding scheme to score activity type by three independent raters	A	Waist	AG-RF-HA/VA/DA-2	*accuracy* = 63.8% RF + HMM: *accuracy* = 64.8% Run/walk: *recall* = 80.0%, *precision* = 88.2% Crawl: *recall *= 81.2%, *precision* = 68.3% Climb: *recall* = 56.0%, *precision* = 29.0% Stand: *recall* = 45.4%, *precision* = 49.2% Sit: *recall* = 66.6%, *precision *= 66.9% Lie down: *recall* = 61.7%, *precision* = 76.0% Carried: *recall* = 58.8%, *precision* = 43.5% Stroller: *recall* = 28.2%, *precision* = 41.0% Sit, stand and stroller collapsed: *accuracy *= 79.3%	-	Very low (level 1)
							AG-SVM-HA/VA/DA-2	*accuracy* = 58.6% SVM + HMM: *accuracy* = 60.1%	-	
							AG-LR-HA/VA/DA-2	*accuracy* = 57.0% LR + HMM: *accuracy* = 59.1%	-	
							AG-J48-HA/VA/DA-2	*accuracy* = 57.3% J48 + HMM: *accuracy* = 57.5%	-	
							AG-kNN-HA/VA/DA-2	*accuracy* = 52.6% kNN + HMM: *accuracy* = 54.1%	-	
	Pulakka et al. (2013) [43]	Validation *n* = 40 *Age* = 16.9 ± 5.8 (16.0 to 18.3) months *Sex* = 60% girls Cross-validation *n* = 30 *Age* = 17.0 ± 0.6 (16 to 18.5) months *Sex* = 60% girls	Activity intensity: SB, LPA, MPA, and VPA Free-living (free play sessions	DVO using CPAF to score activity type and derive activity intensity (SB: stationary, no movement; LPA: stationary, limb movement, MPA: slow trunk movement; VPA: rapid trunk movement) by one rater (and 19.5% of the videos by a second rater)	D	Right hip	**AG-Pul-VM-15**	Validation SB vs. LPA: *AUC-ROC* = .73, *CI* (.67 to .80) VPA vs. MPA: *AUC-ROC* = .67, *CI* (.56 to .78) SB vs LPA and MVPA: *AUC-ROC* = .98, *CI* (.97 to .99) LPA vs. MPA: *AUC-ROC* = .94, *CI* (.91 to .97) Cross-validation SB and LPA vs. MVPA: *accuracy* = 92%, *Se* = 94.2%, *Sp* = 90.9%, *κ* = .83	+ (SB + ; PA ±)	Very low (level 1)
							AG-Pul-VA-15	Validation SB vs. LPA: *AUC-ROC* = .62, *CI* (.56 to .67) VPA vs. MPA: *AUC-ROC* = .59, *CI* (.47 to .72) SB vs. LPA and MVPA: *AUC-ROC* = .95, *CI* (.93 to .96) LPA vs. MPA: *AUC-ROC* = . 90, *CI* (.87 to .94) Cross-validation SB and LPA vs. MVPA: *accuracy* = 84%, *Se* = 84.1%, *Sp* = 84.6%, *κ* = .67	± (SB + ; PA ±)	
	Hager et al. (2016) [42]	*n* = 24 *Age* = 24.5 ± 5.2 (14.7 to 35.5) months *Sex* = 41.7% girls	Activity intensity: SB, LPA, and MVPA Laboratory (structured activities)	DO using CARS to score activity type and derive activity intensity (SB: stationary with no movement; LPA: stationary with movement of the limbs, and slow/easy translocation; MVPA: translocation fast, and with moderate effort) by one rater	D	Left ankle	Ac-Ha-omni-30	*r*_*sp*_ = .75 SB: *Se* = 81.8%, *Sp* = 77.5%; LPA: *Se* = 61.7%, *Sp* = 84.7%; MVPA: *Se* = 85.7%, *Sp* = 88.4%	± (SB ± ; PA +)	Very low (level 2)
	Oftedal et al. (2014) [47]	*n* = 10 *Age* = 29 ± 6 months *Sex* = 50% girls	Activity intensity: SB and non-SB Laboratory (semi-structured activity sessions)	DVO using DO software Behavioral Evaluation Strategy and Taxonomy to score activity type and determine activity intensity (SB: lying/sitting with(out) limb movement, and standing still) by one rater	I	Waist	**AG–O-VM-5**	*Se* = 82%, *Sp* = 83% bias = -5.1%, *LoA* (-27.5 to 16.1%)	SB: +	Very low (level 1)
							AG–O-VA-5	*Se* = 76%, *Sp* = 93%, bias = -17.3%, *LoA* (-44.3 to 8.3%)*	SB: ±	
	Nam & Park (2013) [50]	*n* = 10 *Age* = 22.4 ± 3.3 (16 to 29) months *Sex* = 50% girls	Activity type: Wiggling, rolling, standing still, standing up, sitting down, walking, toddling, crawling, climbing up/down, and stopping (precise definitions not indicated) Laboratory (simulated real home environment)	DVO to score activity type by one rater	I	Hip	mSCA-DT- VA/HA/DA/VM-n.r	*accuracy* = 74.0% (39.5 to 97.2%)	±	Very low (level 1)
							mSCA-NB- VA/HA/DA/VM-n.r	*accuracy* = 73.0% (28.0 to 90.9%)	-	
							**mSCA-BN-VA/HA/DA/VM-n.r**	*accuracy* = 84.8% (57.8 to 98.9%)	+	
							**mSCA-SVM-VA/HA/DA/VM-n.r**	*accuracy* = 86.2% (39.8 to 99.9%)	+	
							**mSCA-kNN-VA/HA/DA/VM-n.r**	*accuracy* = 84.1% (67.8 to 94.6%)	+	
							**mSCA-J48-VA/HA/DA/VM-n.r**	*accuracy* = 88.3% (71.7 to 98.7%)	+	
							mSCA-MLP-VA/HA/DA/VM-n.r	*accuracy* = 84.8% (52.5 to 99.5%)	±	
							**mSCA-MLR-VA/HA/DA/VM-n.r**	*accuracy* = 86.9% (72.1 to 98.7%)	+	

*Abbreviations:* Ac Actical, *AUC-ROC* area under the receiver operating curve, AG ActiGraph, cts counts, BN Bayes net, C Costa’s cut-points (2014) [46], CARS children’s activity rating system, *CCC* concordance correlation coefficient, *CI* confidence interval, CPAF children’s physical activity form, *d’* discriminability index, DA diagonal axis (z-axis), DO direct observation, DVO direct video observation, FFT fast Fourier transform, Ha Hager’s cut-points (2014) [42], HMM hidden Markov model, J48 decision tree (pruned), *κ* Kappa, kNN k-nearest neighbors, *LoA* limits of agreement, LPA light physical activity, LR logistic regression, *MD* mean difference, MLP multi-layer perceptron network, MLR multinomial logistic regression, MPA moderate physical activity, mSCA miniature semiconductor chip accelerometer, MVPA moderate-to-vigorous physical activity, N NHANES cut-points [88], NB naïve Bayes, n.r. not reported, O Oftedal’s cut-points (2014) [47], omni omnidirectional, OSRAC-P observational system for recording physical activity in children preschool, Pa Pate’s cut-points (2006) [52], PA physical activity, Pul Pulakka’s cut-points (2013) [43], Re Reilly’s cut-points (2003)[80], RF random forests, *r*_*sp*_ correlation coefficient (Spearman rank), SB sedentary behavior, *SD* standard deviation, *Se* sensitivity, Si Sirard’s age-specific cut-points (2005) [81], Si3 Sirard’s cut-points (2005) for 3-year-olds [81], *Sp* specificity, SVM support vector machine, T12 Trost’s cut-points (2012) [44], VA vertical axis (y-axis), vC van Cauwenberghe’s cut-points (2011) [45], VM vector magnitude, VPA vigorous physical activity, 5 5 s epoch, 15 15 s epoch, 30 30 s epoch

^**a**^Age presented as mean ± SD (range)

^**b**^Methodological study quality based on newly developed checklist: VG very good, A adequate, D doubtful, I inadequate

^**c**^Device-based method described using code combinations of four elements resulting in the following format: brand-axis-approach-epoch length

^**d**^Study result rating based on COSMIN guideline: + sufficient, ± inconsistent, - insufficient, ? intermediate

^**e**^Quality of evidence based on GRADE approach

^*^ p < *.05*

^**^ p < *.01*

^***^ p < *.001*

For the assessment of SB, LPA and MVPA no valid cut-point sets were found. Four studies evaluated the convergent validity of cut-points based methods for accelerometers using direct (video) observation as comparison measure [43, 44, 46, 47]. These studies suggested that cut-points can be used to distinguish SB [46, 47] or low intensity [43, 44] from high intensity PA. Cut-points to distinguish SB and LPA from MVPA were rated as sufficient, with MVPA for the vector magnitude (VM) ≥ 208 counts/15 s (*M*_*age*_ = 1.42 ± 0.05 years) [43], or for the VA ≥ 418 counts/15 s (*M*_*age*_ = 2.30 ± 0.40 years) [44]. In contrast, cut-points to distinguish SB from total PA were rated as sufficient, for the VM with SB < 6 counts/5 s (*M*_*age*_ = 2.99 ± 0.48 years) [46], or < 40 counts/5 s [47]. These results seemed promising as high agreement and low bias were found, but the results of these studies received low [44, 46] to very low [43, 47] quality of evidence due to small sample sizes (10 ≥ *n* ≤ 40), despite very good methodological quality of two studies [44, 46].

Using a multi-parameter method, SB (e.g., carrying) could be sufficiently distinguished from ambulation PA (e.g., running, crawling, and climbing) using time-domain and frequency acceleration signal features. Convergent validity of this random forests was rated as sufficient [49]. However, these results received low quality of evidence as the results were retrieved in a sample of only 21 toddlers, despite very good methodological study quality. In another study, compared to other multi-parameter methods, random forests provided the best classification of activity type (i.e., running/walking, crawling, climbing, standing, sitting, lying down, carried and stroller) [48]. To improve accuracy, the models were augmented by a hidden Markov model by providing the predictions of the models as observations. Despite small improvements, study results were rated as insufficient.

#### Preschoolers

Table 5 summarizes the results for validity, of which ten studies examined criterion validity [51–55, 66, 67, 75, 84, 88] and thirty studies examined convergent validity [51, 56–64, 68–74, 76–83, 85–87, 89, 90] in preschoolers.

**Table 5:** Criterion and convergent validity of accelerometer-based methods for preschoolers, sorted by methodological study quality, quality of evidence (level of evidence), and study result rating

	**Study**	**Study population**^**a**^	**Outcome(s) & Setting**	**Comparison measure(s)**	**Method** **ological study quality**^**b**^	**Placement**	**Device-based method**^**c**^	**Results**	**Study result rating**^**d**^	**Quality of evidence**^**e**^ **(level of evidence)**
Criterion validity	Adolph et al. (2012) [51]	*n* = 64 *Age* = 4.5 ± 0.8 (3 to 5) years *Sex* = 42.2% girls	cpm Laboratory (structured activities)	Indirect calorimetry using breath-by-breath VO_2_ and VCO_2_ method to determine AEE	VG	Chest	**Ah-cts-uni-60**	*r* = .72***	+	Moderate
						Right hip	**RT-cts-VM-60**	*r* = .80**	+	
							**RT-cts-VA-60**	*r* = .80**	+	
							**RT-cts-HA-60**	*r* = .78**	+	
							**RT-cts-DA-60**	*r* = .74**	+	
	Zakeri et al. (2013) [55]	*n* = 69 *Age* = 4.6 ± 1.0 (3 to 5) years Sex = 50.7% girls	Minute-by-minute EE Laboratory (structured activities)	Indirect calorimetry using breath-by-breath VO_2_ and VCO_2_ to determine EE	VG	Right hip	**AG-cts-VA-60**	*r* = .76***	+	Moderate
							**AG-cts-HA-60**	*r* = .74***	+	
							**AG-cts-DA-60**	*r* = .76***	+	
							**AG-CSTS-VA/HA/DA/VM-60**	*MAPE* = 0.3 ± 6.9%, *CCC* = .93	+	
							**AG-MARS-VA/HA/DA/VM-60**	*MAPE* = 0.3 ± 4.8%, *CCC* = .95	+	
	Bélanger et al. (2013) [66]	*n* = 12 *Age* = 3.1 ± 1.0 (2 to 5) years *Sex* = 66.7% girls	Sleep quality: sleep latency, TST, WASO (number), and sleep efficiency Free-living (at home)	Polysomnography to score sleep stages as sleep and wake	VG	Non-dominant wrist (raw data)	**Aw-AS-VA-30**	Sleep latency: *ICC* = .96** TST: *ICC* = .94**, overestimated by 13 min** WASO: *ICC* = .28, underestimated by 20** Sleep efficiency: *ICC* = .76**, overestimated by 2 min* *Se* = 98.7%, *Sp* = 58.7%, *accuracy* = 95.6%, *NVP* = 81.0%	+	Low
							Aw-ACT40-VA-30	Sleep latency: *ICC* = .92** TST: *ICC* = .94**, underestimated by 25 min** WASO: *ICC* = .42, overestimated by 37** Sleep efficiency: *ICC* = .73**, underestimated by 4 min** *Se* = 92.7%, *Sp* = 69.9%, *accuracy* = 90.7%, *NVP* = 47.4%	±	
							Aw-ACT80-VA-30	Sleep latency: *ICC* = .92** TST: *ICC* = .95* WASO: *ICC* = .32, overestimated by 25** Sleep efficiency: *ICC* = .70** *Se* = 95.8%, *Sp* = 56.7%, *accuracy* = 92.4%, *NVP* = 55.0%	±	
						Non-dominant wrist (adjusted data)	**Aw-ACT40-VA-30**	Sleep latency: *ICC* = .96** TST: *ICC* = .94**, underestimated by 58 min** WASO: *ICC* = .28, overestimated by 35** Sleep efficiency: *ICC* = .76**, underestimated by 9.4 min** *Se* = 87.9%, *Sp* = 81.0%, *accuracy* = 87.5%, *NVP* = 39.4%	+	
							**Aw-ACT80-VA-30**	Sleep latency: *ICC* = .96** TST: *ICC* = .97**, underestimated by 21.5 min** WASO: *ICC* = .36, overestimated by 34** Sleep efficiency: *ICC* = .82**, underestimated by 3.5 min** *Se* = 93.4%, *Sp* = 70.9%, *accuracy* = 91.4%, *NVP* = 49.6%	+	
							Aw-AS-VA-30	Sleep latency: *ICC* = .93** TST: *ICC* = .98** WASO: *ICC* = .36, underestimated by 20** Sleep efficiency: *ICC* = .90** *Se* = 97.7%, *Sp* = 61.2%, *accuracy* = 95.0%, *NVP* = 72.1%	±	
						Left ankle	Aw-ACT40-VA-30	Sleep latency: *ICC* = .83** TST: *ICC* = .94**, underestimated by > 25 min** WASO: *ICC* = .01, overestimated by 36** Sleep efficiency: *ICC* = .80**, underestimated by 6.5 min** *Se* = 90.5%, *Sp* = 75.1%, *accuracy* = 89.3%, *NVP* = 41.7%	±	
							Aw-ACT80-VA-30	Sleep latency: *ICC* = .85** TST: *ICC* = .95** WASO: *ICC* = .09, overestimated by 28** Sleep efficiency: *ICC* = .81** *Se* = 95.0%, *Sp* = 65.0%, *accuracy* = 92.1%, *NVP* = 53.7%	±	
							Aw-AS-VA-30	Sleep latency: *ICC* = .81** TST: *ICC* = .91** WASO: *ICC* = .14, underestimated by 20** Sleep efficiency: *ICC* = .77** *Se* = 97.6%, *Sp* = 57.7%, *accuracy* = 94.6%, *NVP* = 76.8%	±	
	Roscoe, James, & Duncan (2017) [67]	*n* = 21 *Age* = 4.7 ± 0.5 (4 to 5) years *Sex* = 38.1% girls	Activity intensity: SB (< 2 METs), LPA (2–2.99 METs), and MPA (3–5.99 METs) Laboratory (structured activities)	Indirect calorimetry using breath-by-breath VO_2_ and VCO_2_ to determine EE	VG	Non-dominant wrist	GA-Ro-VM-60	SB: *AUC-ROC* = .99, *95% CI* (.98 to 1.0), *Se* = 90%, *Sp* = 90% LPA: *AUC-ROC* = .75, *95% CI* (.65 to .85), *Se* = 40%, *Sp* = 20% MPA: *AUC-ROC* = .92, *95% CI* (.86 to .98), *Se* = 86%, *Sp* = 40%	± (SB + ; PA -)	Low
						Dominant wrist	GA-Ro-VM-60	SB: *AUC-ROC* = .99, *95% CI* (.97 to 1.0), *Se* = 100%, *Sp* = 10% LPA: *AUC-ROC* = .76, *95% CI* (.60 to .86), *Se* = 10%, *Sp* = 85% MPA: *AUC-ROC* = .90, *95% CI* (.83 to .96), *Se* = 76%, *Sp* = 40%	- (SB ± ; PA -)	
	Pfeiffer et al. (2006) [53]	*n* = 18 *Age* = 4.4 ± 0.7 (3 to 5) years *Sex* = 61.0% girls	cpm Laboratory (structured activities)	Indirect calorimetry using breath-by-breath VO_2_	VG	Right hip	**Ac-cts-omni-60**	Validation *r* = .89, *r*_*sp*_ = .90, *R*^*2*^ = .96, *SE* = 3.02, *AIC* = 435.9, *ICC* = .59, *r*_*sp*_ = .80***	+	Very low
			Activity intensity: MPA and VPA Free-living (in- and outdoor activities at preschool)		D		Ac-Pf-omni-60	Cross-validation MPA: a*ccuracy* = 73%, *κ* = .40, modified *κ* = .46 VPA: *accuracy* = 85%, *κ* = .26, modified *κ* = .71	PA: ±	
	Pate et al. (2006) [52]	*n* = 29 *Age* = 4.4 ± 0.8 (3.3 to 5.9) years *Sex* = 46.2% girls	cpm Laboratory (structured activities)	Indirect calorimetry using breath-by-breath VO_2_ and VCO_2_ to determine EE	VG	Right hip	AG-cts-VA-60	Validation *r*_*p*_ = .82, *R*^*2*^ = .90, *SE* = 4.70, *AIC* = 735.8*, ICC* = .57, *r*_*sp*_ = .66***	±	Very low
			Activity intensity: MVPA and VPA Free-living (in- and outdoor activities at preschool)		D		AG-Pa-VA-60	Cross-validation MVPA: *accuracy* = 69%, *Se* = 96.6%, *Sp* = 86.2% *κ* = .36, modified *κ* = .38 VPA: *accuracy* = 81%, *Se* = 65.5%, *Sp* = 95.4%, *κ* = .13, modified *κ* = .62	PA: -	
	Sijtsma et al. (2013) [54]	*n* = 30 *Age* = 3.4 ± 0.3 (3.1 to 4.4) years *Sex* = 60.0% girls	Mean activity cpm (ACM) and mean total activity counts per day (ACD) Free-living (at home for 5 days)	Indirect calorimetry using DLW to determine TEE; breath-by-breath VO_2_ and VCO_2_ to determine SMR to calculate PAL and AEE	D	Middle lower back	T_D_-cts-tri-60	PAL: ACM *r*_*p*_ = .61**, ACD *r*_*p*_ = 46* - ACM, gender & weight, *R*^*2*^ = .50*; ACD, height, gender & sleep duration, *R*^*2*^ = .48* AEE: ACM *r*_*p*_ = .56*, ACD *r*_*p*_ = .38 *-* ACM, *R*^*2*^ = .31*; ACD, gender & sleep duration, *R*^*2*^ = .39* TEE: ACM *r*_*p*_ = .34, ACD *r*_*p*_ = .21 - Multivariate models with ACM & SMR or ACM, ACD & sleep duration not significant Using 5 or 3 days of data did not provide different results for PAL estimation, *MD* = -0.02 ± 0.07, *LoA* (-0.16 to 0.11)	-	Very low
	Ahmadi et al. (2020) [84]	*n* = 31 *Age* = 4.1 ± 1.0 (3 to 5) years *Sex* = 20% girls	EE Free-living (free play)	Indirect calorimetry using breath-by-breath VO_2_ and VCO_2_ to determine EE	A	Non-dominant wrist	AG-RF-VM-10	Free-living (FL) model: *RMSE* = 0.63 (*SD* = 0.47) kcal/min, *RMSE* = 0.96 (*SD* = 0.67) METs, *MAPE* = 27.4% (*SD* = 14.0) Retrained lab (RL) model: *RMSE* = 0.66 (*SD* = 0.47) kcal/min, *RMSE* = 1.01 (*SD* = 0.67) METs, *MAPE* = 28.3% (*SD* = 15.0) Existing lab, FL and RL models: mean bias not different from zero, predicted EE within ± 6% of measured EE	?	Very low
							AG-SVM-VM-10	Free-living (FL) model: *RMSE* = 0.64 (*SD* = 0.51) kcal/min, *RMSE* = 0.99 (*SD* = 0.73) METs, *MAPE* = 25.4% (*SD* = 12.2) Retrained lab (RL) model: *RMSE* = 0.65 (*SD* = 0.54) kcal/min, *RMSE* = 0.99 (*SD* = 0.77) METs, *MAPE* = 26.0% (*SD* = 12.6) Existing lab, FL and RL models: mean bias not different from zero, predicted EE within ± 6% of measured EE	?	
						Right hip	AG-RF-VM-10	Free-living (FL) model: *RMSE* = 0.63 (*SD* = 0.42) kcal/min, *RMSE* = 0.96 (*SD* = 0.59) METs, *MAPE* = 28.1% (*SD* = 12.0) Retrained lab (RL) model: *RMSE* = 0.67 (*SD* = 0.41) kcal/min, *RMSE* = 1.02 (*SD* = 0.57) METs, *MAPE* = 28.3% (*SD* = 12.7) FL and RL models: EE overestimated during play sessions with low total EE and underestimated during sessions with high total EE Existing lab models: mean bias not different from zero, predicted EE within ± 6% of measured EE	?	
							AG-ANN-VM-10	Free-living (FL) model: *RMSE* = 0.63 (*SD* = 0.43) kcal/min, *RMSE* = 0.96 (*SD* = 0.61) METs, *MAPE* = 27.1% (*SD* = 11.1) Retrained lab (RL) model: *RMSE* = 0.65 (*SD* = 0.44) kcal/min, *RMSE* = 0.99 (*SD* = 0.62) METs, *MAPE* = 28.4% (*SD* = 11.8) Existing lab, FL and RL models: EE overestimated during play sessions with low total EE and underestimated during sessions with high total EE	?	
	Steenbock et al. (2019) [88]	*n* = 41 *Age* = 4.8 ± 0.8 (3 to 6) years *Sex* = 46% girls	Absolute (kJ/min) and relative (kg/min/J) EE Free-living (in- and outdoor semi-structured activities at school)	Indirect calorimetry using breath-by-breath VO_2_ and VCO_2_ to determine EE	A	Right hip	AG-LM- HA/VA/DA-30	*RMSE* = 2.91 (*SD* = 0.95) kJ/min, *RMSE* = 124.91 (*SD* = 30.58) J/min/kg, *RMSE* = 1.70 (*SD* = 0.39) METs	?	Very low
							AG-MLM- HA/VA/DA-30	*RMSE* = 2.91 (*SD* = 0.99) kJ/min, *RMSE* = 125.40 (*SD* = 31.50) J/min/kg, *RMSE* = 1.70 (*SD* = 0.40) METs	?	
							AG-RF- HA/VA/DA-30	*RMSE* = 2.74 (*SD* = 0.96) kJ/min, *RMSE* = 115.56 (*SD* = 27.35) J/min/kg, *RMSE* = 1.56 (*SD* = 0.36) METs	?	
							AG-ANN- HA/VA/DA-30	*RMSE* = 2.86 (*SD* = 0.95) kJ/min, *RMSE* = 121.51 (*SD* = 29.57) J/min/kg, *RMSE* = 1.66 (*SD* = 0.39) METs	?	
							GA-LM- HA/VA/DA-30	*RMSE* = 2.89 (*SD* = 0.95) kJ/min, *RMSE* = 123.79 (*SD* = 31.28) J/min/kg, *RMSE* = 1.68 (*SD* = 0.39) METs	?	
							GA-MLM- HA/VA/DA-30	*RMSE* = 2.90 (*SD* = 0.98) kJ/min, *RMSE* = 124.70 (*SD* = 32.95) J/min/kg, *RMSE* = 1.69 (*SD* = 0.42) METs	?	
							GA-RF- HA/VA/DA-30	*RMSE* = 2.73 (*SD* = 1.00) kJ/min, *RMSE* = 112.57 (*SD* = 28.83) J/min/kg, *RMSE* = 1.53 (*SD* = 0.38) METs	?	
							GA-ANN- HA/VA/DA-30	*RMSE* = 2.83 (*SD* = 0.98) kJ/min, *RMSE* = 118.04 (*SD* = 29.80) J/min/kg, *RMSE* = 1.66 (*SD* = 0.43) METs	?	
						Left hip	AG-LM- HA/VA/DA-30	*RMSE* = 2.84 (*SD* = 0.94) kJ/min, *RMSE* = 123.64 (*SD* = 31.82) J/min/kg, *RMSE* = 1.67 (*SD* = 0.41) METs	?	
							AG-MLM- HA/VA/DA-30	*RMSE* = 2.81 (*SD* = 0.99) kJ/min, *RMSE* = 123.58 (*SD* = 31.94) J/min/kg, *RMSE* = 1.67 (*SD* = 0.41) METs	?	
							AG-RF- HA/VA/DA-30	*RMSE* = 2.60 (*SD* = 0.97) kJ/min, *RMSE* = 112.32 (*SD* = 28.40) J/min/kg, *RMSE* = 1.52 (*SD* = 0.38) METs	?	
							AG-ANN- HA/VA/DA-30	*RMSE* = 2.78 (*SD* = 1.01) kJ/min, *RMSE* = 116.89 (*SD* = 28.41) J/min/kg, *RMSE* = 1.63 (*SD* = 0.42) METs	?	
						Right wrist	GA-LM- HA/VA/DA-30	*RMSE* = 2.85 (*SD* = 0.83) kJ/min, *RMSE* = 125.60 (*SD* = 28.52) J/min/kg, *RMSE* = 1.69 (*SD* = 0.38) METs	?	
							GA-MLM- HA/VA/DA-30	*RMSE* = 2.83 (*SD* = 0.87) kJ/min, *RMSE* = 125.42 (*SD* = 28.74) J/min/kg, *RMSE* = 1.69 (*SD* = 0.38) METs	?	
							GA-RF- HA/VA/DA-30	*RMSE* = 2.56 (*SD* = 0.83) kJ/min, *RMSE* = 109.34 (*SD* = 26.98) J/min/kg, *RMSE* = 1.48 (*SD* = 0.37) METs	?	
							GA-ANN- HA/VA/DA-30	*RMSE* = 2.74 (*SD* = 0.88) kJ/min, *RMSE* = 115.91 (*SD* = 28.55) J/min/kg, *RMSE* = 1.62 (*SD* = 0.40) METs	?	
						Non-dominant wrist	GA-LM- HA/VA/DA-30	*RMSE* = 2.83 (*SD* = 0.86) kJ/min, *RMSE* = 125.21 (*SD* = 27.38) J/min/kg, *RMSE* = 1.70 (*SD* = 0.37) METs	?	
							GA-MLM- HA/VA/DA-30	*RMSE* = 2.83 (*SD* = 0.89) kJ/min, *RMSE* = 124.91 (*SD* = 27.58) J/min/kg, *RMSE* = 1.69 (*SD* = 0.38) METs	?	
							GA-RF- HA/VA/DA-30	*RMSE* = 2.56 (*SD* = 0.83) kJ/min, *RMSE* = 108.64 (*SD* = 26.33) J/min/kg, *RMSE* = 1.47 (*SD* = 0.36) METs	?	
							GA-ANN- HA/VA/DA-30	*RMSE* = 2.72 (*SD* = 0.91) kJ/min, *RMSE* = 114.01 (*SD* = 27.88) J/min/kg, *RMSE* = 1.61 (*SD* = 0.44) METs	?	
						Right thigh	aP-LM- HA/VA/DA-30	*RMSE* = 2.94 (*SD* = 0.91) kJ/min, *RMSE* = 126.22 (*SD* = 29.46) J/min/kg, *RMSE* = 1.69 (*SD* = 0.38) METs	?	
							aP-MLM- HA/VA/DA-30	*RMSE* = 2.92 (*SD* = 0.95) kJ/min, *RMSE* = 125.96 (*SD* = 29.86) J/min/kg, *RMSE* = 1.69 (*SD* = 0.39) METs	?	
							aP-RF- HA/VA/DA-30	*RMSE* = 2.76 (*SD* = 0.94) kJ/min, *RMSE* = 115.61 (*SD* = 27.93) J/min/kg, *RMSE* = 1.56 (*SD* = 0.38) METs	?	
							aP-ANN- HA/VA/DA-30	*RMSE* = 3.08 (*SD* = 1.00) kJ/min, *RMSE* = 125.98 (*SD* = 31.50) J/min/kg, *RMSE* = 1.81 (*SD* = 0.40) METs	?	
	Butte et al. (2014) [75]	*n* = 50 *Age* = 4.5 ± 0.8 (3 to 5) years *Sex* = 50.0% girls	cpm Laboratory (structured activities)	Indirect calorimetry using breath-by-breath VO_2_ and VCO_2_ to determine minute-by-minute EE	I	Right hip	**AG-CSTS-VA/HA/DA/VM-60**	*MAPE* = -0.2 ± 6.7%, MAE = -0.005 ± 0.07 kcal/min, *RMSE* = 0.07 kcal/min, *R*^*2*^ = .88	+	Very low
							AG-MARS-VA/HA/DA/VM-60	*MAPE* = 1.1 ± 6.6%, MAE = 0.009 ± 0.07 kcal/min, *RMSE* = 0.07 kcal/min	?	
		*n* = 105 *Age* = 4.6 ± 0.9 years *Sex* = 66.7% girls	Activity intensity: SB, LPA, MPA, and VPA Free-living	Indirect calorimetry using DLW to determine AEE	I		AG-B-VM-60	*accuracy* = 70% SB: *Sp* = 83% LPA: *Sp* = 64% MPA: *Sp* = 35% VPA: *Sp* = 38% MVPA collapsed: *accuracy* = 74%	- (SB + ; PA -)	
							AG-B-HA-60	*accuracy* = 68% SB: *Sp* = 82% LPA: *Sp* = 58% MPA: *Sp* = 37% VPA: *Sp* = 27% MVPA collapsed: *accuracy* = 71%	- (SB + ; PA -)	
						Chest	Ah-B-HA-60	*accuracy* = 70%; *Sp*: SB: *Sp* = 81% LPA: *Sp* = 64% MPA: *Sp* = 48% VPA: *Sp* = 39% MVPA collapsed: *accuracy* = 74%	- (SB + ; PA -)	
Convergent validity	Sirard et al. (2005) [81]	*n* = 269 (*n* = 69 3-, *n* = 125 4-, *n* = 75 5-year-olds) *Sex* = 58% 3-, 52% 4-, 52% 5-year-old girls	Activity intensity: SB, LPA, MPA and VPA Free-living (at preschool)	DVO using CARS to score activity type and derive activity intensity (SB: stationary, motionless or with trunk/limb movements; LPA: slow/easy movement; MPA: moderate movement; VPA: fast movement) by a single rater (beginning and end of video by four additional raters)	VG	Right hip	**AG-Si-VA-15**	Total counts: *r*_*p*_ = .58*** SB: *r*_*p*_ = .70***, *Se* (94.4 to 100%), *Sp* (91.7 to 100%) LPA: *r*_*p*_ = .59*** VPA: *r*_*p*_ = .61***, *Se* (95.8 to 100%), *Sp* (80.0 to 83.3%) MPA: *r*_*p*_ = .50***, *Se* (86.7 to 94.4%), *Sp* (66.7 to 100%) MVPA: *r*_*p*_ = .46***	+ (SB + ; PA +)	High (level 1)
	Peirera et al. (2020) [74]	*n* = 60 *Age* = 2.7 ± 0.4 (1.8 to 3.5) years *Sex* = 50% girls	% time spent in SB Free-living (at childcare)	aP-PRE-uni-n.r. at right thigh (SB: sitting/lying)	VG	Hip		All outside the ± 10% equivalent interval of (-4.05 to 4.05)		Moderate (level 2)
							AG-C-VA-5	bias = -5.11, *90% CI* (-7.90 to -2.32)	?	
							AG-Ev-VA-15	bias = 8.15, *90% CI* (5.44 to 10.86)	?	
							AG-T12-VA-15	bias = 4.46, *90% CI* (1.65 to 7.28)	?	
							AG-K-VA-15	bias = -7.95, *90% CI* (-11.01 to -4.89)	?	
							AG-Pa-VA-15	bias = 6.17, *90% CI* (3.40 to 8.93)	?	
							AG-Pa2-VA-15	bias = -9.26, *90% CI* (-12.32 to -6.21)	?	
							AG-Re-VA-60	bias = -40.52, *90% CI* (-43.82 to -37.22)	?	
							AG-Si-VA-15	bias = -15.07*, 90% CI* (-18.16 to -11.97)	?	
							AG-vC-VA-15	bias = -18.71, *90% CI* (-21.89 to -15.54)	?	
	Dobell et al. (2019) [58]	*n* = 62 *Age* = 3.5 ± 0.5 (3 to 5) years *Sex* = 45.5% girls	Activity intensity: SB, LPA, and MVPA Free-living (free-play in nursery)	DO using OSRAC-P (modified) to score activity type and derive activity intensity (SB: stationary/ motionless; LPA: stationary torso with limb and slow/easy movement; MVPA: moderate and fast movement) by two independent raters	VG	Non-dominant wrist	AG-cts-VM-5/10/15/30	*r*_*sp*_ = .34, *R*^*2*^ = .11* (5 s) to *r*_*sp*_ = .48, *R*^*2*^ = .23* (30 s) SB: *AUC-ROC* (.71 to .78), *Se* (.64 to .68), *Sp* (.64 to .78) LPA: *AUC-ROC* (.55 to .56), *Se* (.74 to .80), *Sp* (.33 to .43) MVPA: *AUC-ROC* (.67 to .78), *Se* (.63 to .80), *Sp* (.80 to .82)	- (SB -; PA -)	Moderate (level 2)
						Right hip	AG-cts-VA-5/10/15/30	*r*_*sp*_ = .38, *R*^*2*^ = .16* (5 s) to *r*_*sp*_ = .52, *R*^*2*^ = .27* (10 s) SB: *AUC-ROC* (.69 to .77), *Se* (.61 to .72), *Sp* (.69 to .72) LPA: *AUC-ROC* (.53 to .54), *Se* (.59 to .76), *Sp* (.39 to .50) MVPA: *AUC-ROC* (.71 to .81), *Se* (.55 to .70), *Sp* (.77 to .79)	- (SB -; PA -)	
							AG-cts-VM-5/10/15/30	*r*_*sp*_ = .40, *R*^*2*^ = .16* (5 s) to *r*_*sp*_ = .55, *R*^*2*^ = .30* (30 s) SB: *AUC-ROC (*.73 to .79), *Se* (.69 to .72), *Sp* (.67 to .75) LPA: *AUC-ROC* = .55, *Se* (.78 to .82), *Sp* (.35 to .36) MVPA: *AUC-ROC* (.72 to .82), *Se* (.68 to .78), *Sp* (.62 to .79)	- (SB -; PA -)	
	Ahmadi, Pavey, & Trost (2020) [85]	*n* = 31 *Age* = 4.0 ± 0.9 (3 to 5) years *Sex* = 29.0% girls	Activity intensity: SB, LPA, MVPA, run and walk Free-living (20 min active play session)	DVO using customized scheme to score activity type and derive activity intensity (SB: sitting/lying down, and stationary, motionless; LPA: standing, stationary, movement of the limbs/trunk, easy translocation, MPA: moderate and fast translocation; walk: translocation, medium speed; run: translocation (very) fast)	VG	Right hip	AG-RF-VM-1	Time and frequency features: *F-score* = 70.6%, + temporal features: *F-score* = 75.8% SB: *F-score* = 73.3%, + temporal: *F-score* = 80.0% LPA: *F-score* = 76.0%, + temporal: *F-score* = 80.4% MVPA: *F-score* = 61.0%, + temporal: *F-score* = 65.2% Walk: *F-score* = 55.7%, + temporal: *F-score* = 62.9% Run: *F-score* = 53.1%, + temporal: *F-score* = 67.2%	-	Low (level 1)
							AG-RF-VM-5	Time and frequency features: *F-score* = 79.5%, + temporal features: *F-score* = 82.2% SB: *F-score* = 80.6%, + temporal: *F-score* = 85.7% LPA: *F-score* = 82.7%, + temporal: *F-score* = 85.6% MVPA: *F-score* = 75.5%, + temporal: *F-score* = 76.3% Walk: *F-score* = 69.8%, + temporal: *F-score* = 68.8% Run: *F-score* = 71.2%, + temporal: *F-score* = 72.8%	- (incl. temp oral ±)	
							AG-RF-VM-10	Time and frequency features: *F-score* = 83.1%, + temporal features: *F-score* = 85.3% SB: *F-score* = 82.3%, + temporal: *F-score* = 87.6% LPA: *F-score* = 85.7%, + temporal: *F-score* = 87.9% MVPA: *F-score* = 77.6%, + temporal: *F-score* = 76.4% Walk: *F-score* = 80.7%, + temporal: *F-score* = 81.0% Run: *F-score* = 74.4%, + temporal: *F-score* = 73.6%	±	
							AG-RF-VM-15	Time and frequency features: *F-score* = 84.0%, + temporal features: *F-score* = 85.9% SB: *F-score* = 85.0%, + temporal: *F-score* = 87.7% LPA: *F-score* = 86.8%, + temporal: *F-score* = 88.2% MVPA: *F-score* = 75.3%, + temporal: *F-score* = 78.5% Walk: *F-score* = 78.4%, + temporal: *F-score* = 79.4% Run: *F-score* = 80.0%, + temporal: *F-score* = 82.6%	±	
						Non-dominant wrist	AG-RF-VM-1	Time and frequency features: *F-score* = 62.6%, + temporal features: *F-score* = 68.8% SB: *F-score* = 63.3%, + temporal: *F-score* = 69.2% LPA: *F-score* = 71.4%, + temporal: *F-score* = 75.5% MVPA: *F-score* = 45.7%, + temporal: *F-score* = 57.5% Walk: *F-score* = 45.9%, + temporal: *F-score* = 54.8% Run: *F-score* = 55.1%, + temporal: *F-score* = 61.6%	-	
							AG-RF-VM-5	Time and frequency features: *F-score* = 70.8%, + temporal features: *F-score* = 75.5% SB: *F-score* = 69.3%, + temporal: *F-score* = 78.% LPA: *F-score* = 76.5%, + temporal: *F-score* = 80.1% MVPA: *F-score* = 60.9%, + temporal: *F-score* = 66.9% Walk: *F-score* = 60.7%, + temporal: *F-score* = 60.3% Run: *F-score* = 68.5%, + temporal: *F-score* = 68.8%	-	
							AG-RF-VM-10	Time and frequency features: *F-score* = 74.5%, + temporal features: *F-score* = 80.0% SB: *F-score* = 73.7%, + temporal: *F-score* = 82.4% LPA: *F-score* = 79.1%, + temporal: *F-score* = 82.9% MVPA: *F-score* = 62.0%, + temporal: *F-score* = 70.3% Walk: *F-score* = 68.8%, + temporal: *F-score* = 70.8% Run: *F-score* = 73.4%, + temporal: *F-score* = 71.5%	- (incl. temp oral ±)	
							AG-RF-VM-15	Time and frequency features: *F-score* = 77.3%, + temporal features: *F-score* = 80.6% SB: *F-score* = 78.2%, + temporal: *F-score* = 83.3% LPA: *F-score* = 81.2%, + temporal: *F-score* = 83.7% MVPA: *F-score* = 62.1%, + temporal: *F-score* = 70.7% Walk: *F-score* = 70.5%, + temporal: *F-score* = 69.0% Run: *F-score* = 82.4%, + temporal: *F-score* = 82.4%	- (incl. temp oral ±)	
						Right hip + Non-dominant wrist	AG-RF-VM-1	Time and frequency features: *F-score* = 72.5%, + temporal features: *F-score* = 76.8% SB: *F-score* = 75.8%, + temporal: *F-score* = 80.8% LPA: *F-score* = 77.7%, + temporal: *F-score* = 81.0% MVPA: *F-score* = 62.1%, + temporal: *F-score* = 66.7% Walk: *F-score* = 58.4%, + temporal: *F-score* = 64.9% Run: *F-score* = 64.5%, + temporal: *F-score* = 67.9%	-	
							AG-RF-VM-5	Time and frequency features: *F-score* = 79.6%, + temporal features: *F-score* = 82.1% SB: *F-score* = 81.6%, + temporal: *F-score* = 86.0% LPA: *F-score* = 82.8%, + temporal: *F-score* = 85.6% MVPA: *F-score* = 74.3%, + temporal: *F-score* = 74.5% Walk: *F-score* = 70.1%, + temporal: *F-score* = 69.5% Run: *F-score* = 71.7%, + temporal: *F-score* = 72.5%	- (incl. temp oral ±)	
							AG-RF-VM-10	Time and frequency features: *F-score* = 83.2%, + temporal features: *F-score* = 85.3% SB: *F-score* = 83.9%, + temporal: *F-score* = 87.5% LPA: *F-score* = 85.8%, + temporal: *F-score* = 87.9% MVPA: *F-score* = 75.6%, + temporal: *F-score* = 77.2% Walk: *F-score* = 79.5%, + temporal: *F-score* = 80.7% Run: *F-score* = 74.9%, + temporal: *F-score* = 73.0%	- (incl. temp oral ±)	
							AG-RF-VM-15	Time and frequency features: *F-score* = 84.3%, + temporal features: *F-score* = 86.4% SB: *F-score* = 85.5%, + temporal: *F-score* = 88.7% LPA: *F-score* = 86.4%, + temporal: *F-score* = 88.4% MVPA: *F-score* = 77.1%, + temporal: *F-score* = 78.0% Walk: *F-score* = 78.1%, + temporal: *F-score* = 79.8% Run: *F-score* = 86.6%, + temporal: *F-score* = 86.8%	±	
	Hislop et al. (2012) [70]	*n* = 31 *Age* = 4.4 ± 0.8 years *Sex* = 51.6% girls	cpm and % time spent in MVPA Free-living (unstructured outdoor free play during preschool)	DVO using CARS (modified) to score activity type and derive activity intensity (MVPA: moderate/fast translocation) by one rater	VG	Right hip	AG-cts-VA-15	*r*_*sp*_ = .39*	-	Low (level 1)
							RT-cts-VM-15	*r*_*sp*_ = .56**	-	
							AG-Si-VA-15	*MD* = 0.8 min*	?	
							RT-Swr-VM-15	*MD* = -12.2 min*	?	
							RT-Slj-VM-15	*MD* = 0 min	?	
	Hislop et al. (2012) [61]	*n* = 31 *Age* = 4.4 ± 0.8 years *Sex* = 51.6% girls	Activity intensity: SB, LPA, and MVPA Free-living (unstructured outdoor free play during preschool)	DVO using CARS to score activity type and derive activity intensity (SB: stationary, motionless or with movement of limbs/trunk; LPA: easy translocation; MVPA: moderate/fast translocation) by one rater	VG	Right hip	AG-Ev-VA-15	SB: *MD* = 13.2, *LoA* (-2.2 to 28.6)**	?	Low (level 1)
							AG-Si-VA-15	SB: *MD* = -7.2, *LoA* (-20.2 to 5.7)**; LPA: *MD* = 6.3, *LoA* (-6.0 to 18.6)** MVPA: *MD* = 0.8, *LoA* (-6.2 to 7.8)	?	
							AG-Puy-VA-15	SB: *MD* = 0.4, *LoA* (-13.25 to 13.99); LPA: *MD* = -2.2, *LoA* (-15.0 to 10.5) MVPA: *MD* = 1.7, *LoA* (-8.0 to 11.5)	?	
							AG-vC-VA-15	SB: *MD* = -8.2, *LoA* (-22.7 to 6.2)**; LPA: *MD* = 11.4, *LoA* (-1.4 to 24.2)** MVPA: *MD* = -3.3, *LoA* (-11.8 to 5.3)**	?	
							AG-Pa-VA-15	MVPA: *MD* = -8.7, *LoA* (-19.9 to 2.5)**	?	
							AG-Re-VA-15	SB: *MD* = -3.6, *LoA* (-17.6 to 10.4)**	?	
	Hislop et al. (2016) [76]	*n* = 32 *Age* = 4.20 ± 0.5 (3 to 5) years *Sex* = 34.4% girls	Activity intensity: SB, LPA, MVPA and total PA Free-living (free-play in nursery)	DVO using CARS (modified) to score activity type and derive activity intensity (SB: stationary, motionless; LPA: stationary, movement of the trunk/limbs and easy translocation; MVPA: moderate and fast translocation)	VG	Non-dominant wrist	AG-J13-VM-5	Total PA: *MD* = 1.1, *LoA* (-9.9 to 12.1) Locomotion CARS vs. high intensity: *MD* = -2.0, *LoA* (-9.4 to 5.4) No clear agreement MVPA: *MD* = -9.3, *LoA* (-20.0 to 1.5); Locomotion vs. high wrist estimate: *MD* = 3.4, *LoA* (-3.1 to 10.0); Standing vs. low wrist estimate: *MD* = 2.8, *LoA* (-10.3 to 16.4)	?	Low (level 1)
						Waist	AG-Ev-VA-5	No clear agreement: Total PA: *MD* = 6.3, *LoA* (-8.8 to 21.4); MVPA: *MD* = -3.8, *LoA* (-10.7 to 3.1); Locomotion: *MD* = 3.4, *LoA* (-3.1 to 10.0); Standing: *MD* = 2.8, *LoA* (-10.3 to 16.4)	?	
	Martin et al. (2011) [72]	*n* = 23 *Age* = 4.5 ± 0.7 years *Sex* = 60.9% girls	SB and total PA Free-living	aP-PRE-uni-n.r. at right thigh (SB: sitting/lying, and quiet standing)	VG	Right hip	AG-Re-VA-60	*r*_*sp*_ = .66***, *MD* = -2.1 ± 4.6%, *LoA* (-11.4 to 7.2%)* Corrected (counts increased by 9%): *r*_*sp*_ = .68***, *MD* = -4.3 ± 4.8%, *LoA* (-14.0 to 5.4%)***	+	Low (level 2)
	van Cauwenberghe et al. (2012) [64]	*n* = 49 *Age* = 4.0 ± 0.5 (3 to 4) years *Sex* = 55.1% girls	SB and total PA Free-living (at childcare)	aP-PRE-uni-n.r. at right thigh (SB: sitting/lying)	VG	Right hip	Ac-Ev-omni-15	*accuracy* = 73.0%, *κ* = .46, *95% CI* (.45 to .47) *MD* = 1.3 min; 0.1%, *LoA* (-59.4 to 61.9); 39.2%	-	Low (level 2)
	Kahan, Nicaise, & Reuben (2013) [71]	*n* = 12 *Age* = 4.7 ± 0.3 years *Sex* = 75.0% girls	Activity intensity: SB and MVPA Free-living (unstructured outdoor free play)	DO using OSRAC-P to score activity type and derive activity intensity (SB: stationary, motionless or with limb/trunk movement, light activity; MVPA: moderate/ vigorous activity) scored by one rater	VG	Right hip	AG-Si-VA-5	*accuracy* = 81.2, 95% *CI* (79.2 to 83.1), *κ* = .48 SB: *Se* = 81.4%, *Sp* = 50.7%, bias = -8.1% MVPA: *Se* = 35.8%, *Sp* = 87.6%, bias = 4.7%	± (SB ± ; PA ±)	Low (level 2)
							AG-vC-VA-5	*accuracy* = 78.5, *95% CI* (76.4 to 80.3), *κ* = .48 SB: *Se* = 80.0%, *Sp* = 52.4%, bias = -8.6% MVPA: *Se* = 53.9%, *Sp* = 79.1%, bias = -5.3%	- (SB ± ; PA -)	
							AG-Ev-VA-5	*accuracy* = 79.9, *95% CI* (77.5 to 82.1), *κ* = .58 SB: *Se* = 75.2%, *Sp* = 66.4%, bias = 36.3%*** MVPA: *Se* = 54.8%, *Sp* = 78.9%, bias = -5.9%	- (SB -; PA -)	
							AG-Pa-VA-5	*accuracy* = 77.2, *95% CI* (74.9 to 79.4), *κ* = .53 SB: *Se* = 50.0%, *Sp* = 58.7%, bias = 33.2%*** MVPA: *Se* = 62.7%, *Sp* = 72.2%, bias = -15.8%***	- (SB -; PA -)	
	Alhassan et al. (2017) [56]	*n* = 33 *Age* = 4.4 ± 0.8 (2.9 to 5) years *Sex* = 36.4% girls	Counts Free-living (at preschool)	Aw-cts-uni-15 at non-dominant wrist	VG	Waist	AG-cts-VA-15	*r*_*sp*_ = .41***	-	Low (level 2)
			Activity intensity: SB, LPA, MPA, and VPA Free-living (at preschool)	DO using OSRAC-P (modified) to score activity type and derive activity intensity (SB: stationary, motionless or with trunk/limb movement; LPA: easy translocation; MPA: moderate translocation; VPA: vigorous translocation) by one rater	A VG (for MVPA)		AG-cts-VA-15	*r*_*sp*_ = .47***	-	Low (MVPA) Very low (rest) (level 2)
							AG-Si-VA-15	*accuracy* = 50.3%, *κ* = .13, *κ*_*w*_ = .19 SB: *accuracy* = 62.7%, *κ* = .26, *Se* = 87.4%, *Sp* = 38.2% LPA: a*ccuracy* = 65.0%, *κ* = .01, *Se* = 18.5%, *Sp* = 82.2% MPA: *accuracy* = 86.3%, *κ* = .06, *Se* = 10.0%, *Sp* = 95.1% VPA: *accuracy* = 86.5%, *κ* = .06, *Se* = 5.3%, *Sp* = 98.5% MVPA: *accuracy* = 76.9%, *κ* = .15, *Se* = 16.2%, *Sp* = 95.2%	- (SB -; PA -)	
							AG-Pa-VA-15	*accuracy* = 44.1%, *κ* = .21, *κ*_*w*_ = .32 SB: *accuracy* = 61.5%, *κ* = 0.40, *Se* = 60.1%, *Sp* = 79.7% LPA: *accuracy* = 60.7%, *κ* = .13, *Se* = 51.1%, *Sp* = 64.3% MPA: *accuracy* = 80.3%, *κ* = .09, *Se* = 24.0%, *Sp* = 86.8% VPA: *accuracy* = 86.1%, *κ* = .19, *Se* = 18.0%, *Sp* = 96.2% MVPA: *accuracy* = 75.3%, *κ* = .27, *Se* = 39.8%, *Sp* = 86.0%	- (SB -; PA -)	
						Non-dominant wrist	Aw-cts-uni-15	*r*_*sp*_ = .47***	-	
							Aw-Ek-uni-15	*accuracy* = 50.3%, *κ* = .13, *κ*_*w*_ = .19 SB: *accuracy* = 65.47%, *κ* = 0.31, *Se* = 41.5%, *Sp* = 89.1% LPA: *accuracy* = 53.8%, *κ* = .10, *Se* = 62.3%, *Sp* = 50.7% MPA: *accuracy* = 82.8%, *κ* = .11, *Se* = 22.0%, Sp = 89.7% VPA: *accuracy* = 86.3%, *κ* = .31, *Se* = 33.9%, *Sp* = 94.0% MVPA: *accuracy* = 77.5%, *κ* = .35, *Se* = 46.9%, *Sp* = 86.8%	- (SB -; PA -)	
	Ahmadi et al. (2020) [83]	*n* = 31 *Age* = 4.0 ± 0.9 (3 to 5) years *Sex* = 29% girls	Activity intensity: SB, LPA, MVPA, run, and walk Free-living (play session)	DVO using customized scheme to score activity type and derive activity intensity (SB: sitting/lying down, and stationary, motionless; LPA: standing, stationary, movement of the limbs/trunk, easy translocation, MPA: moderate and fast translocation; walk: translocation, medium speed; run: translocation (very) fast)	A	Non-dominant wrist	AG-RF-VM-15	*accuracy* = 59.1%, *95% CI* (57.1 to 61.1%), *κ* = .37 SB: *accuracy* = 76.8% LPA: *accuracy* = 57.5% MVPA: *accuracy* = 83.6% Walk: *accuracy* = 14.8% Run: *accuracy* = 68.7% Excl. multiple activities: walk *accuracy* (14.8 to 43.8%) and run *accuracy* (68.7 to 100%)	- (SB -; PA -)	Very low (level 1)
							AG-SVM-VM-15	*accuracy* = 59.3%, *95% CI* (57.3 to 61.3%), *κ* = .37 SB: *accuracy* = 76.8% LPA: *accuracy* = 58.3% MVPA: *accuracy* = 82.6% Walk: *accuracy* = 12.3% Run: *accuracy* = 70.1% Excl. multiple activities: walk *accuracy* (12.3 to 45.8%) and run *accuracy* (70.1 to 100%)	- (SB -; PA -)	
						Right hip	AG-RF-VM-15	*accuracy* = 69.4%, *95% CI* (67.4 to 71.2%), *κ* = .48 SB: *accuracy* = 71.7% LPA: *accuracy* = 79.3% MVPA: *accuracy* = 71.3% Walk: *accuracy* = 10.4% Run: *accuracy* = 74.5% Excl. multiple activities: walk *accuracy* (10.4 to 29.2%) and run *accuracy* (74.5 to 100%)	- (SB -; PA -)	
							AG-SVM-VM-15	*accuracy* = 66.4%, *95% CI* (64.4 to 68.3%), *κ* = .45 SB: *accuracy* = 77.7% LPA: *accuracy* = 71.1% MVPA: *accuracy* = 73.0% Walk: *accuracy* = 8.9% Run: *accuracy* = 65.8% Excl. multiple activities: walk *accuracy* (8.9 to 33.3%) and run *accuracy* (65.8 to 100%)	- (SB -; PA -)	
	Trost et al. (2018) [89]	*n* = 11 *Age* = 4.8 ± 0.87 (3 to 6) years *Sex* = 55% girls	Activity intensity: SB, LPA, MVPA, run, and walk Laboratory (semi-standardized activities	DO to score activity type and determine activity intensity based on preschool-specific MET thresholds (SB < 1.5 METs, LPA 1.5–2.7, MPA 2.8–3.4 VPA ≥ 3.5)	A	Non-dominant wrist	**AG-SVM-VM-15**	*accuracy* = 80.4%, *95% CI* (78.9 to 81.9%), *κ*_*w*_ = .73, *CV κ*_*w*_ = .78 SB: *accuracy* = 90.0% LPA: *accuracy* = 73.7% MVPA: *accuracy* = 78.6% Walk: *accuracy* = 70.6% Run: *accuracy* = 71.2%	+ (SB + ; PA -)	Very low (level 2)
							AG-RF-VM-15	*accuracy* = 78.1%, *95% CI* (76.6 to 79.9%), *κ*_*w*_ = .70, *CV κ*_*w*_ = .76 SB: *accuracy* = 89.1% LPA: *accuracy* = 68.7% MVPA: *accuracy* = 79.1% Walk: *accuracy* = 61.1% Run: *accuracy* = 68.8%	± (SB + ; PA -)	
							AG-T18-VA-15	*κ*_*w*_ = .64	-	
							AG-T18-VM-15	*κ*_*w*_ = .49	-	
						Right hip	**AG-SVM-VM-15**	*accuracy* = 81.3%, 95% *CI* (79.9 to 82.8%), *κ*_*w*_ = .74, CV κ_w_ = .82 SB: *accuracy* = 94.0% LPA: *accuracy* = 76.8% MVPA: *accuracy* = 76.0% Walk: *accuracy* = 63.5% Run: *accuracy* = 69.8%	+ (SB + ; PA -)	
							AG-RF-VM-15	*accuracy* = 80.2%, *95% CI* (79.9 to 81.6%), *κ*_*w*_ = .72, *CV κ*_*w*_ = .81 SB: *accuracy* = 93.2% LPA: *accuracy* = 72.1% MVPA: *accuracy* = 76.7% Walk: *accuracy* = 61.1% Run: *accuracy* = 73.0%	± (SB + ; PA -)	
							AG-T18-VA-15	*κ*_*w*_ = .65	-	
							AG-T18-VM-15	*κ*_*w*_ = .61	-	
						Non-dominant wrist + hip	**AG-SVM-VM-15**	*accuracy* = 85.2%, *95% CI* (83.8 to 86.5%), *κ*_*w*_ = .80, *CV κ*_*w*_ = .84 SB: *accuracy* = 94.7% LPA: *accuracy* = 80.2% MVPA: *accuracy* = 81.7% Walk: *accuracy* = 69.7% Run: *accuracy* = 82.3%	+ (SB + ; PA +)	
							**AG-RF-VM-15**	*accuracy* = 81.8%, *95% CI* (80.4 to 83.2%), *κ*_*w*_ = .75, *CV κ*_*w*_ = .81 SB: *accuracy* = 93.0% LPA: *accuracy* = 73.9% MVPA: *accuracy* = 80.7% Walk: *accuracy* = 63.0% Run: *accuracy* = 75.3%	+ (SB + ; PA +)	
	Finn & Specker (2000) [60]	*n* = 40 *Age* = (3 to 4) years *Sex* = 60% girls	Mean activity counts (3-min) Free-living	DVO using CARS (3-min average) to score activity type and derive activity intensity (SB: stationary, motionless or with movement of limbs/trunk; LPA: easy translocation; MVPA: moderate/fast translocation) by two independent raters	D	Waist	Aw-cts-uni-60	*r* = .03 to *r* = .92 (median = .74) More active children higher association between CARS and counts: within-child correlation coefficients vs. CARS *r* = .37* or counts *r* = .31	PA: -	Very low (level 1)
	Brønd et al. (2020) [86]	*n* = 29 *Age* = 4.4 (3 to 6) years *Sex* = n.r. % girls	Activity type: Sit, stand, run, walk, and bike Free-living (structured activities at school)	DO to score activity type (sit: on floor/chair playing; stand: stand close to table playing; run: run together with instructor; walk: walks together with instructor; bike: on running bike) by one rater	D	Thigh	**AX-DT-HA/VA/DA-2**	Sit: *Se* = 100%, *Sp* = 100% Stand: *Se* = 100%, *Sp* = 99.8% Bike (sitting bike): *Se* = 64.8%, *Sp* = 100% Walk: *Se* = 82.6%, *Sp* = 98.1% Run: *Se* = 92.4%, *Sp* = 95.0%	+	Very low (level 2)
	Zhao et al. (2013) [90]	*n* = 69 *Age* = (3 to 5) years *Sex* = 55% girls	Activity type: Sleep, rest, quiet play, low active play, moderately active play, and very active play Laboratory (structured activities)	DO to score activity type (sleep: lying and sleeping; rest: watching tv, quiet play: sitting while coloring, puzzle, video game; low active play: stand and play; moderately active play: standing while ball toss, active video game, dance; very active play: running in place)	D	Right hip	AG-SVM -VA/HA/DA/VM-60	*accuracy* = 75.3% Sleep: *accuracy* = 91.44% Rest: *accuracy* = 65.66% Quiet play: *accuracy* = 74.07% Low active play: *accuracy* = 68.49% Moderately active play: *accuracy* = 93.73% Very active play: *accuracy* = 98.73% Misclassification highest for sleep (31.6%) and quiet play (33.0%)	±	Very low (level 2)
							AG-MLR—VA/HA/DA/VM-60	*accuracy* (67.2 to 73.2%)	-	
	Hagenbuchner et al. (2015) [87]	*n* = 11 *Age* = 4.8 ± 0.87 (3 to 6) years *Sex* = 55% girls	Activity intensity: SB, LPA, MVPA, run, and walk Laboratory (activity trials)	DO to score activity type and derive activity intensity (SB: watching tv, tablet/computer, reading, quiet play; LPA: clean up toys, standing art, treasure hunt; MVPA: bicycle riding, obstacle course, active game; walk: walk at self-selected pace; run: run at self-selected speed)	D	Right hip	AG-MLP-VM-60	*accuracy* = 69.7% SB: *accuracy* = 81.8% LPA: *accuracy* = 78.8% MVPA: *accuracy* = 63.6% Walk: *accuracy* = 36.4% Run: *accuracy* = 45.5%	- (SB + ; PA -)	Very low (level 2)
							AG-SOM-VM-60	*accuracy* = 53.8% SB: *accuracy* = 59.1% LPA: *accuracy* = 57.6% MVPA: *accuracy* = 66.7% Walk: *accuracy* = 36.4% Run: *accuracy* = 0.0%	- (SB -; PA -)	
							AG-DLEN-VM-60	*accuracy* = 82.6% SB: *accuracy* = 84.1% LPA: *accuracy* = 90.9% MVPA: *accuracy* = 78.8% Walk: *accuracy* = 72.7% Run: *accuracy* = 72.7%	± (SB + ; PA -)	
	de Bock et al. (2010) [68]	*n* = 33 *Age* = 3.5 ± 0.8 (boys); 4.3 ± 1.1 (girls) years *Sex* = 36.36% girls	Activity intensity: SB and MVPA Free-living (at preschool)	DO using CARS to score activity type and derive activity intensity (SB: stationary, motionless or with trunk/limb movement; MVPA: easy, moderate, and fast translocation) by five independent raters	D	Chest	Ah-dB-VA-15	Calibration MVPA: *AUC-ROC* = .86 SB: *AUC-ROC* = .79 Cross-validation SB: *accuracy* 67% (boys), 69% (girls); *Sp* = 52% (boys), 61% (girls); *Se* = 78% (boys), 75% (girls) MVPA: *accuracy* = 84% (boys), 87% (girls)	- (SB -; PA +)	Very low (level 2)
	Fairweather et al. (1999) [59]	*n* = 11 *Age* = 3.7 ± 0.5 years *Sex* = 72.7% girls	Mean cpm Free-living (exercise class)	DO using 1-min average CPAF score (ranging from 1 to 4) scored by one rater	D	Right hip	**CSA-cts-uni-60**	*r* = .87***, *r*_*sp*_ = .79**	PA: +	Very low (level 2)
		*n* = 10 *Age* = 4.0 ± 0.4 years *Sex* = 90% girls		CSA at left hip				*r* = .92**, *MD* = 31 (*SD* = 64, *95% CI* (1 to 61)*, *r*_*sp*_ = .97** Relative PA assessment largely unaffected by location	PA: +	
	Klesges & Klesges (1987) [62]	*n* = 28 *Age* = 33.9 ± 8.0 months *Sex* = 43.3% girls	Total PA (all-day) and hourly PA Free-living (unstructured activities)	DO using FATS to score behavior and intensity	D	Left hip	CPAC-cts-uni-n.r	*r* = .54*** (total PA); *r* = .62** to .95*** (hourly PA) Behavior: lying *r*_*sp*_ = -.37; sitting *r*_*sp*_ = -.26; standing *r*_*sp*_ = .60**; walking *r*_*sp*_ = .50**; running *r*_*sp*_ = .37 Intensity: minimal *r*_*sp*_ = -.51**; moderate *r*_*sp*_ = .51**; extreme *r*_*sp*_ = .40* Summary: correlations higher for older children (< 32.5 months *r*_*sp*_ = .39; > 32.5 months *r*_*sp*_ = .80***), girls (*r*_*sp*_ = ,67*, boys *r*_*sp*_ = .43), and obese children (*r*_*sp*_ = .75*, normal weight *r*_*sp*_ = .46*)	PA: ±	Very low (level 2)
	Klesges et al. (1985) [63]	*n* = 30 *Age* = 47.7 ± 12.5 months *Sex* = 40% girls	Total PA Free living (free play)	DO using FATS to score behavior and intensity	D	Left hip	CPAC-cts-uni-n.r	Behavior: *r* = .38*, *r*_*s*p_ = .28: lying *r* = .17, *r*_*sp*_ = -.18; sitting *r* = -.31*, *r*_*sp*_ = -.32*; crawling *r* = -.22, *r*_*sp*_ = -.20; climbing *r* = -.06, *r*_*sp*_ = .02; standing *r* = .35*, *r*_*sp*_ = .37*; walking *r* = -.11, *r*_*sp*_ = -.03; running *r* = .32*, *r*_*sp*_ = .22 Intensity: *r* = .32*, *r*_*sp*_ = .29: minimal *r* = -.27, *r*_*sp*_ = -.23; moderate *r* = .05, *r*_*sp*_ = .07; extreme *r* = .44**, *r*_*sp*_ = .43** Summary: *r* = .40**, *r*_*sp*_ = .36*	PA: -	Very low (level 2)
						Right hip	LSI-cts-uni-n.r	Behavior: *r* = .38*, *r*_*s*p_ = .28: lying *r* = .17, *r*_*sp*_ = -.18; sitting *r* = -.31*, *r*_*sp*_ = -.32*; crawling *r* = -.22, *r*_*sp*_ = -.20; climbing *r* = -.06, *r*_*sp*_ = .02; standing *r* = .35*, *r*_*sp*_ = .37*; walking *r* = -.11, *r*_*sp*_ = -.03; running *r* = .32*, *r*_*sp*_ = .22 Intensity: *r* = .32*, *r*_*sp*_ = .29: minimal *r* = -.27, *r*_*sp*_ = -.23; moderate *r* = .05, *r*_*sp*_ = .07; extreme *r* = .44**, *r*_*sp*_ = .43** Summary: *r* = .40**, *r*_*sp*_ = .36*	PA: -	
	Ettienne et al. (2016) [69]	*n* = 30 *Age* = 3.5 ± 0.6 years *Sex* = 43% girls	Activity intensity: SB, LPA, and MVPA Free-living (during preschool)	DO using (modified) SOFIT to score activity type and derive activity intensity (SB: lying; LPA: sitting, standing; MVPA: walking, very active)	D	Non-dominant wrist	Ac-Sc-omni-60	*accuracy* = 74%, *κ*_*w*_ = .17*** 391 min classified as MVPA, whereas DO class was LPA; 406 min DO classified as MVPA and accelerometer as LPA	-	Very low (level 2)
	Djafarian et al. (2013) [57]	*n* = 42 *Age* = 4.1 ± 0.8 (3 to 5) years *Sex* = 47.6% girls	cpm 1–10-min periods Free-living (structured & unstructured)	DO using 1-min average CARS score	D	Non-dominant wrist	Aw-cts-uni-300	*r* = .52** (*n* = 21 cross-validation sample) Measured (DO) vs. predicted (log accelerometry) CARS score *MD* = 0.025 (± 0.38), *CI 95%* with 0.74 CARS unit	PA: -	Very low (level 2)
	Davies et al. (2012) [82]	*n* = 30 *Age* = 4.1 ± 0.5 (3.1 to 4.9) years *Sex* = 60% girls	Activity type: Sit/lie, stand, and walk Free-living (usual nursery setting)	DVO using to score activity type (walk, sit, stand, lie, other (crouching down, kneeling up, crawling) by one rater	I	Right thigh	**aP-PRE-uni-1**	Time spent: sitting *MD* = -4.4%**, standing *MD* = 7.1%** Sit/lie: *Se* = 86.7%, *Sp* = 97.1%, *PPV* = 96.3% Stand: *Se* = 91.8%, *Sp* = 84.3%, *PPV* = 75.4% Walk: *Se* = 80.3%, *Sp* = 95.9%, *PPV* = 78.4%	+	Very low (level 1)
	Johansson et al. (2015) [77]	Calibration *n* = 26 *Age* = 26 ± 6.0 months *Sex* = 38% girls Cross-validation *n* = 12 *Age* = 25 ± 5.6 (15 to 36) months *Sex* = 50% girls	Activity intensity: SB, LPA, and MVPA Free-living (structured activities and free play at preschool)	DVO using CARS to score activity type and derive activity intensity (SB: stationary, motionless; LPA: stationary, movement of the limbs/trunk, easy translocation; MVPA: moderate and fast translocation)	I	Non-dominant wrist	**AG-J13-VM-5**	Calibration SB: *AUC-ROC* = .98, *CI* (.95 to 1.0) MVPA: *AUC-ROC* = .90, *CI* (.81 to .98) Cross-validation SB: *r*_*sp*_ = .91 LPA: *r*_*sp*_ = .77* MVPA: *r*_*sp*_ = .69*	+ (SB + ; PA ±)	Very low (level 1)
							**AG-J13-VA-5**	Calibration SB: *AUC-ROC* = .98, *CI* (.95 to 1.0) MVPA: *AUC-ROC* = .88, *CI* (.78 to .97) Cross-validation SB: *r*_*sp*_ = .91 LPA: *r*_*sp*_ = .89* MVPA: *r*_*sp*_ = .77*	+ (SB + ; PA +)	
	Johansson et al. (2016) [78]	*n* = 30 *Age* = 49.0 ± 3.7 months *Sex* = 53% girls	Activity intensity: SB, LPA, and MVPA Free-living (structured indoor activities and free play at preschool)	DO using CARS to score activity type and derive activity intensity (SB: stationary, motionless; LPA: stationary, movement of the limbs/trunk, easy translocation; MVPA: moderate and fast translocation) by one rater	I	Left hip	**AG-J16-VM-5**	*κ*_*qw*_ = .86, *95% CI* (.85 to .87), *accuracy* = 75% SB: *Se* = 100%, *Sp* = 60%, *AUC-ROC* = .95 MVPA: *Se* = 70%, *Sp* = 93%, *AUC-ROC* = .91	+ (SB ± ; PA ±)	Very low (level 2)
							AG-J16-VA-5	*κ*_*qw*_ = .76, *95% CI* (.74 to .77), *accuracy* = 68% SB: *Se* = 100%, *Sp* = 60%, *AUC-ROC* = .93 MVPA: *Se* = 70%, *Sp* = 100%, *AUC-ROC* = .95	± (SB ± ; PA ±)	
						Non-dominant wrist	**AG-J16-VM-5**	*κ*_*qw*_ = .95, *95% CI* (.94 to .96), *accuracy* = 82% SB: *Se* = 100%, *Sp* = 60%, *AUC-ROC* = .99 MVPA: *Se* = 70%, *Sp* = 100%, *AUC-ROC* = .96	+ (SB ± ; PA ±)	
							**AG-J16-VA-5**	*κ*_*qw*_ = .92, *95% CI* (.91 to .93), *accuracy* = 82% SB: *Se* = 100%, *Sp* = 60%, *AUC-ROC* = .99 MVPA: *Se* = 70%, *Sp* = 100%, *AUC-ROC* = .95	+ (SB ± ; PA ±)	
	Reilly et al. (2003) [80]	*n* = 52 *Age* = 3.5 ± 0.5 (3 to 4) years *Sex* = 59.62% girls	Activity intensity: SB Free-living	DO using CPAF to score activity type and derive activity intensity (SB: stationary, motionless or with limb movement) by one rater	I	Right hip	**CSA-Re-uni-60**	*Se* = 83%, *95% CI* (78 to 86%) *Sp* = 82%, *95% CI* (89 to 86%)	SB: +	Very low (level 2)
	Adolph et al. (2012) [51]	*n* = 64 *Age* = 4.5 ± 0.8 (3 to 5) years *Sex* = 42.2% girls	Activity intensity: SB, LPA, and MVPA Laboratory	DO using CARS to score activity type and derive CARS levels (1 to 5)	I	Chest	Ah-A-uni-60	*accuracy* = 72%, *TPR* = 82% SB: *accuracy* = 75% LPA: *accuracy* = 61% MVPA: *accuracy* = 82%	- (SB -; PA ±)	Very low (level 2)
						Right hip	Ac-A-omni-60	*accuracy* = 71%, *TPR* = 69% SB: *accuracy* = 77% LPA: *accuracy* = 63% MVPA: *accuracy* = 69%	- (SB -; PA -)	
							RT-A-VM-60	*accuracy* = 73%, *TPR* = 79% SB: *accuracy* = 76% LPA: *accuracy* = 65% MVPA: *accuracy* = 79%	- (SB -; PA ±)	
	Li et al. (2020) [79]	*n* = 34 *Age* = 4.0 ± 0.5 (3 to 5) years *Sex* = 58.8% girls	Activity intensity: SB, LPA, MPA, and VPA Free-living (during preschool)	AG-B-VM-60	I	Right hip	AG-Li_ROC_-VM-60	*κ* = .30, *accuracy* = 59.5% SB: *Se* = 77.8%, *Sp* = 77.8%, *FPR* = 22.2%, *FNR* = 22.2%, *κ* = .54 LPA: *Se* = 26.8%, *Sp* = 87.5%, *FPR* = 12.5%, *FNR* = 73.2%, *κ* = .16 MPA: *Se* = 4.5%, *Sp* = 69.8%, *FPR* = 3.2%, *FNR* = 95.5%, *κ* = .02 VPA: = *Se* = 79.0%, *Sp* = 79.0%, *FPR* = 21.0%, *FNR* = 21.0%, *κ* = .11 MVPA collapsed: *κ* = .35, *accuracy* = 63.6%, *Se* = 78.9%, *Sp* = 78.9%, *FPR* = 20.1%, *FNR* = 21.1%, *κ* = .28	- (SB -; PA -)	Very low (level 2)
							AG-Li_OLR_-VM-60	*κ* = .37, *accuracy* = 71.5% SB: *Se* = 91.0%, *Sp* = 52.8%, *FPR* = 47.2%, *FNR* = 9.1%, *κ* = .47 LPA: *Se* = 43.3%, *Sp* = 86.5%, *FPR* = 56.7%, *FNR* = 13.5%, *κ* = .32 MPA: *Se* = 8.8%, *Sp* = 99.1%, *FPR* = 1.0%, *FNR* = 76.1%, *κ* = .12 VPA: *Se* = 23.9%, *Sp* = 99.0%, *FPR* = 1.0%, *FNR* = 76.1%, *κ* = .28 MVPA collapsed: *κ* = .39, *accuracy* = 72.4%, *Se* = 24.3%, *Sp* = 99.0%, *FPR* = 1.0%, *FNR* = 75.7%, *κ* = .33	- (SB -; PA -)	
				AG-J13-VM-60		Non-dominant wrist	AG-Li_4k_-VM-60	*κ* = .37, *accuracy* = 63.7% SB: *Se* = 71.6%, *Sp* = 85.1%, *FPR* = 14.9%, *FNR* = 28.4%, *κ* = .53 LPA: *Se* = 50.9%, *Sp* = 76.8%, *FPR* = 23.2%, *FNR* = 49.1%, *κ* = .27 MPA: *Se* = 48.4%, *Sp* = 86.8%, *FPR* = 13.2%, *FNR* = 51.6%, *κ* = .19 VPA: *Se* = 33.9%, *Sp* = 98.2%, *FPR* = 1.8%, *FNR* = 66.1%, *κ* = .31 MVPA collapsed: *κ* = .40, *accuracy* = 65.6%, *Se* = 68.4%, *Sp* = 86.6%, *FPR* = 13.4%, *FNR* = 31.6%, *κ* = .35	- (SB -; PA -)	
	Pagels, Boldemann, & Raustorp (2011) [73]	*n* = 55 *Age* = 4.5 ± 0.6 (3.4 to 5.7) years *Sex* = 49.1% girls	Activity intensity: LPA and MVPA Free-living (at preschool)	Pedometer (Yamax SW200) to measure daily steps	I	Waist	AG-Si-VA-15	*r* = .67***, *R*^*2*^_*adj*_ = 45%: (3-year-olds: *r* = .37, 4-year-olds: *r* = .52**, 5-year-olds: *r* = .84***) LPA: *r* = .76***, *R*^*2*^_*adj*_ = 52% MVPA: 3-year-olds (*r* = .19, *R*^*2*^_*adj*_ = 4%); 4-to-5-year-olds (*r* = .50***, *R*^*2*^_*adj*_ = 23%)	PA: -	Very low (level 3)

*Abbreviations:* A Adolph’s cut-points (2012) [51], Ac Actical, ACT40 Actiware software with wake threshold value of 40, ACT80 Actiware software with wake threshold value of 80, AEE activity energy expenditure, *AIC* Akaike information criterion, AG ActiGraph, Ah Actiheart, ANN artificial neural network, aP activPAL, AS AlgoSmooth [94], *AUC-ROC* area under the receiver operating curve, Aw Actiwatch, AX Axivity, B Butte’s cut-points (2014) [75], C Costa’s cut-points (2014) [46], CARS children’s activity rating system, *CCC* concordance correlation coefficient, *CI* confidence interval, CPAC Caltrac personal activity computer, CPAF children’s physical activity form, cpm counts per minute, cts counts, CSA computer science and applications activity monitor, CSTS cross-sectional time series, *CV* cross-validation, DA diagonal axis (z-axis), dB de Bock’s cut-points (2010) [68], DLEN deep learning ensemble network, DLW doubly labelled water, DO direct observation, DT decision table, DVO direct video observation, EE energy expenditure, Ek Ekblom’s cut-points (2012) [95], Ev Evenson’s cut-points (2008) [91], FATS Fargo activity time-sampling survey, *FNR* false negative rate, *FPR* false positive rate, GA GENEActiv, HA horizontal axis (x-axis), *ICC* intraclass correlation coefficient, J13 Johansson’s cut-points (2015) [77], J16 Johansson’s cut-points (2016) [78], *κ* Kappa, K Kelly’s cut-points (2016) [96], *κ*_*w*_ weighted Kappa, *κ*_*qw*_ quadratic weighted Kappa, Li_ROC_ Li’s cut-points (2020) [79], Li_ORL_ Li’s cut-points (2020) [79], Li_4k_ Li’s cut-points (2020) [79], *LoA* limits of agreement, LM linear model, LPA light physical activity, LSI LSI moving activity monitor, *MAE* mean absolute error, *MAPE* mean absolute percentage error, MARS multivariate adaptive regression splines, *MD* mean difference, METs metabolic equivalents, MLM mixed linear model, MLP multi-layer perceptron network, MLR multinomial logistic regression, MPA moderate physical activity, MVPA moderate-to-vigorous physical activity, n.r. not reported, *NVP* negative predictive value, omni omnidirectional, OSRAC-P observational system for recording physical activity in children preschool, Pa Pate’s cut-points (2006) [52], PA physical activity, Pa2 Pate’s cut-points (2006) [52], PAL physical activity level, Pf Pfeiffer’s cut-points (2006) [53], *PPV* positive predictive value, PRE activPAL professional research edition software, Puy Puyau’s cut-points (2002) [97], *r* correlation coefficient (unknown), Re Reilly’s cut-points (2003) [80], RF random forests, Ro Roscoe’s cut-points (2017) [67], *r*_*p*_ correlation coefficient (Pearson), *r*_*sp*_ correlation coefficient (Spearman rank), *R*^*2*^ R-squared value, *R*.^*2*^_*adj*_ adjusted R-squared value, *RMSE* root mean square error, RT Research Tracker 3, SB sedentary behavior, Sc Schaefer’s cut-points (2014) [98], *SD* standard deviation, *Se* sensitivity, *SE* standard error, Si Sirard’s age-specific cut-points (2005) [81], Slj Sun’s cut point (2008) [99], SMR sleeping metabolic rate, SOFIT system for observing fitness instruction time, SOM self-organizing map, *Sp* specificity, SVM support vector machine, Swr Sun’s cut point (2008) [99], T_D_ Tracmor_D_, TEE total energy expenditure, tri mean of the three axes, *TPR* true positive rate, TST total sleep time, T12 Trost’s cut-points (2012) [44], T18 Trost’s cut-points (2018) [89], uni uniaxial (axis not specified), VA vertical axis (y-axis), vC van Cauwenberghe’s cut-points (2011) [45], VPA vigorous physical activity, VCO_2_ carbon dioxide, VM vector magnitude, VO_2_ oxygen consumption, WASO wake after sleep onset, 1 1 s epoch, 2 2 s epoch, 5 5 s epoch, 10 10 s epoch, 15 15 s epoch, 30 30 s epoch, 60 60 s epoch, 300 5 min epochs

^**a**^Age presented as mean ± SD (range)

^**b**^Methodological study quality based on newly developed checklist: VG very good, A adequate, D doubtful, I inadequate

^**c**^Device-based method described using code combinations of four elements resulting in the following format: brand-axis-approach-epoch length

^**d**^Study result rating based on COSMIN guideline: + sufficient, ± inconsistent, - insufficient, ? intermediate

^**e**^Quality of evidence based on GRADE approach

^*^ p < *.05*

^**^ p < *.01*

^***^ p < *.001*

For the assessment of SB, LPA and MVPA, hip [81] and wrist [78] cut-points were evaluated as valid. Sirard and colleagues (2005) evaluated convergent validity of age specific cut-points [81]. Compared to observational scores using the Children’s Activity Rating Scale (CARS) agreement was high. Highest correlation between predicted and observed scores was found for SB, whereas lowest correlation between predicted and observed scores was found for MVPA. These results received high quality of evidence as the methodological study quality was very good and results were retrieved in a sample of 269 preschoolers. Similar results for convergent validity of Sirard’s SB and MVPA cut-points were found, using the Observational System for Recording physical Activity in Children-Preschool (OSRAC-P) as comparison measure [71]. Highest sensitivity was found for SB, while sensitivity was lowest for VPA. However, specificity was lowest for SB and highest for MVPA. Compared to other cut-points based methods, Sirard’s cut-points were most sensitive in detecting SB and converged best with direct observation [45, 52, 71, 91]. However, convergent validity of Sirard’s cut-points was rated as inconsistent with moderate quality of evidence, as these results were retrieved in a sample of 56 preschoolers. Another study evaluated convergent validity of the Sirard MVPA cut-point compared to the same direct observation scheme, i.e., CARS [61]. Bias was lowest when applying the Sirard MVPA cut-point versus other cut-points based methods in a sample of only 32 preschoolers [45, 52, 97]. When applied to accelerometer-derived data in toddlers, results of Sirard’s cut-points were rated as insufficient [45].

Johansson and colleagues (2016) evaluated convergent validity of wrist cut-points (both VA and VM) [78]. Agreement with direct observation (i.e., CARS) was high [78]. However, these results received very low quality of evidence, as the methodological study quality was inadequate, and results were retrieved in a limited sample of 30 preschoolers.

Some multi-parameter methods were also suitable for assessing SB, LPA and MVPA. Convergent validity of a support vector machine was evaluated as sufficient for distinguishing SB, LPA and MVPA, walking and running in laboratory setting [89] but not in free-living settings [83, 85]. Trost and colleagues (2018) developed random forest and support vector machine models to categorize SB, light activities and games (i.e., LPA), moderate-to-vigorous activities and games (i.e., MVPA), walking and running [89]. Almost perfect agreement with direct observation was found (hip and combination hip and wrist), while cut-points for this sample resulted in only moderate to substantial agreement. Using hip or wrist only data, convergent validity of support vector machines was rated as sufficient, while both random forests and support vector machines were rated as sufficient when hip and wrist data were combined. In free-living settings accuracy of these models decreased by 11 to 15% [85]. Hip classifiers had moderate agreement with direct video observation, while agreement of wrist classifiers was lower in free-living. In free-living settings, accuracy of random forests combining hip and wrist data was comparable. However, study results were rated as insufficient. In addition, support vector machine models have not been tested while combining hip and wrist data. Despite very good [85] and adequate [83, 89] methodological study quality, these results received low quality of evidence as the results were retrieved in limited samples of 11 [89] and 31 [83, 85] preschoolers. Convergent validity of a simple decision table was evaluated as sufficient for distinguishing comparable activity types (i.e., sit, stand, run, walk and bike) in a free-living setting [86]. These study results received very low quality of evidence as methodological study quality was doubtful and the results were retrieved in a sample of only 29 preschoolers.

Criterion validity of support vector machine models, random forests, and artificial neural networks was evaluated [84, 88]. Despite intermediate study results, random forests and artificial neural networks seemed to result in equal prediction accuracy of energy expenditure [88]. Results of the other study indicated that predicted energy expenditure of existing lab, free-living and retrained random forests (wrist and hip) and support vector machines (wrist) were within ± 6% of measured energy expenditure, but not for artificial neural networks (hip) [84]. Despite adequate methodological study quality, these study results received very low quality of evidence as they were retrieved in limited samples of 31 [84] and 41 [88] preschoolers. Criterion validity of cross-sectional time series models [55, 75] and multivariate adaptive regression splines [55] was evaluated as sufficient for predicting minute-by-minute energy expenditure. Results indicated a lack of bias and acceptable limits of agreement for these models, however, bias was slightly lower for multivariate adaptive regression splines. These results received moderate [55] and very low [75] quality of evidence.

For sleep assessment, different cut-points based methods (i.e., wake threshold values) were evaluated to distinguish sleep from wake. Moreover, the multi-parameter method AlgoSmooth was evaluated, which smooths data derived from the wake threshold value of 40 before scoring sleep or wake [100]. Criterion validity of wake threshold values of 40 and 80 applied to adjusted wrist data (i.e., regressed using lower activity counts of the ankle device) was rated as sufficient for wake time but poor for wake time after onset, but results were rated as inconsistent when applied to raw data [66]. In contrast, when the raw wrist data was smoothed using the AlgoSmooth algorithm, criterion validity was evaluated as sufficient [66]. Correlations between an Actiwatch and polysomnography were high, except for wake time after onset. Results were worse for ankle data. Despite very good methodological study quality, these results received low quality of evidence as the results were retrieved in a limited sample of 12 preschoolers [66].

### Convergence between devices

Few studies examined the convergence between different accelerometers [56, 64, 72, 74]. No studies evaluated convergence between accelerometer-based methods in infants and toddlers. Convergence of both time spent in SB derived by ActiGraph and activPAL (hip vs. thigh) [72, 74], Actical and activPAL (wrist vs. thigh) [64], and ActiGraph and Actiwatch (waist vs. wrist) count data [56] was rated as insufficient as low to moderate agreement was found. Despite very good methodological study quality of all studies, these results received moderate [74] to low [56, 64, 72] quality of evidence due to sample sizes of 23 to 60 preschoolers.

### Combining data

Combining data derived from multiple sensors [39, 85, 89] or multiple axes [34, 43, 47, 49, 77, 78] resulted in more valid predictions. Single placed sensors resulted in lower performance and insufficient data for posture and movement classification, compared to combining data from two or four different locations [39]. In line with these results, the combination of hip and wrist data in models for activity intensity prediction resulted in higher performance compared to using hip or wrist data only [85, 89]. Despite very good [85] and adequate [39, 89] methodological study quality, these results received (very) low quality of evidence due to limited sample sizes of 31 [85], 22 [39], and 11 [89] children.

Generally, using data from the VM to categorize activity intensity provided better results (higher agreement between accelerometry and comparison measure) than the VA (y-axis) [43, 47, 77, 78]. In line with these results, for activity type classification (i.e., running walking climbing up/down, crawling, riding on a ride on toy, standing, sitting, stroller, being carried) feature importance was highest for the VM standard deviation versus features of the different axes or other VM features [49]. In addition, combined cut-points for the VA (y-axis) and horizontal axis (x-axis) were used to classify the postures of infants as prone and non-prone [34]. Despite the inconsistent results, the study indicated that combining data from these axes is required to accurately assess horizontal movement behaviors such as tummy time (time spent prone on floor). The combination of the acceleration signal time-domain and frequency features also resulted in better activity type classification [49, 50]. When comparing feature sets, accuracy was higher when frequency-domain features were included in addition to time-domain features. Moreover, activity intensity classification improved when adding temporal features to this base set (i.e., time domain and frequency features) [85].

## Discussion

This review summarizes studies that evaluated the measurement properties of accelerometer-based methods for assessing physical behavior in young children (< 5 years old). To assess the methodological quality of the 62 included studies, we developed a new checklist inspired by COSMIN [25–27]. Despite very good to adequate methodological study quality of 58% of the studies, only ten percent of the study results received high or moderate quality of evidence, due to limited samples sizes.

Validated cut-points for the youngest age group (i.e., infants) are still lacking, while multi-parameter methods were evaluated as sufficient to distinguish posture, SB and PA using multiple sensors [39], movement and sleep [40], leg movements [41], and sleep–wake [30, 36, 38]. Despite very good [30] or adequate [39, 40] methodological study quality of some of these studies, quality of evidence was rated as low [30] to very low [36, 38–41].

In toddlers, hip cut-points were considered valid for distinguishing SB and LPA [46, 47] from high intensity PA [43, 44], despite large differences between cut-points based methods. No studies were found for identifying sleep in toddlers using cut-points based or multi-parameter methods. For SB, VM cut-points varied between < 40 counts/5 s [47] and < 97 counts/5 s [46]. For MVPA, cut-points were VM ≥ 208 counts/15 s [43] and VA ≥ 418 counts/15 s [44]. The difference between the SB cut-points is probably due to inconsistency in the definition of SB adopted by the different observational schemes used. Specifically, in the study by Costa and colleagues, SB was defined as “stationary with no movement and stationary with movement of the limbs”, resulting in a higher cut-point [46] than Oftedal and colleagues who did not include limb movement [47]. Notably, the MVPA cut-points were derived in study samples with divergent characteristics. Trost and colleagues included children from an urban area that were around one year older than the children from a rural area included by Pulakka and colleagues [43, 44]. Conversely, cut-points could not identify toddler specific behaviors such as “being carried” as SB. A random forest model was considered valid to distinguish SB (including this toddler specific behavior) from PA [49]. Despite very good methodological study quality of some studies [44, 46, 49], quality of evidence for these cut-points based and multi-parameter methods was low.

For distinguishing different physical intensities (i.e., SB, LPA, and MVPA), we found strongest evidence in preschoolers. Next to age-specific hip cut-points [81], wrist cut-points were also evaluated as valid [78]. For assessing sleep, adjusted wrist [66] cut-points were evaluated as valid. Quality of evidence for hip cut-points was high [81], and these cut-points were also positively evaluated in cross-validation studies [61, 71], but not when applied in toddlers [45]. The wrist cut-points, however, have not been cross-validated and despite very good methodological study quality of the study assessing sleep cut-points [66], quality of evidence was low [66, 78]. Conversely, for identifying SB, LPA, and MVPA, using multi-parameter methods resulted in more promising results compared to cut-points based methods [83, 85, 89]. Although random forest and support vector machine models were rated as sufficient in laboratory setting [89], these were rated as insufficient in a free-living setting [83, 85]. Activity type could be distinguished using a decision table in a free-living setting [86]. In addition, sleep could be distinguished from wake using the AlgoSmooth algorithm applied to (raw) wrist data [66]. Despite very good [66, 85] and adequate [83, 86, 89] methodological study quality, quality of evidence was low [66, 85] or very low [83, 86, 89].

Despite the promising results of multi-parameter methods, only few models were accessible as open-source software [84–86, 88]. This hampers the replication of study results as closed source models cannot be reused. In case software is available, be aware to use the same version, configuration, and implementation (e.g., brand, axis, placement, parameters, target group). It is not recommended to reuse cut-points based methods when deviating from accelerometer specifications (i.e., brand, axis, and placement) or target population (age group), as cut-points need to be re-evaluated. Moreover, the inconvenience of cut-points based methods is related to the derivation of the magnitude of acceleration (count data), which is kept close source by most manufacturers.

Most studies used a single device or axis to measure physical behavior, while more promising results were found when combining data derived from different sensor placements [39, 85, 89] or multiple axes [34, 43, 47, 49, 50, 90]. Movement patterns of young children are sporadic, omnidirectional, and unique per developmental stage (e.g., lying on back or tummy, crawling or walking), and accelerometers capture only the movement of the body segment it is attached to [15, 20, 23]. This requires accelerometers that can capture movement in multiple planes, and placement of accelerometers on different sites (e.g., wrist and hip, legs, and arms).

There are a few reasons that contributed to the (very) low quality of evidence of the studies. Firstly, the quality of evidence was mostly downgraded due to limited sample sizes of < 100 children included in studies, i.e., imprecision [26]. If more studies would have used the same measurement and analyses protocol (i.e., accelerometer brand, accelerometer data analysis approach, axis, and epoch length), this sample size issue could have been resolved by pooling the results. Besides sample size, another important aspect for the generalizability is that the variation of the performed physical behaviors in the target population is captured. Secondly, the doubtful methodological study quality contributed to the downgrading of the quality of evidence, i.e., risk of bias [26]. Common methodological limitations varied per measurement property. Regarding convergent validity, both the unknown or insufficient measurement properties of the comparison instrument and the too long epoch lengths [101] resulted in low methodological study quality. For most studies that used direct observation as comparison measure, interrater agreement was insufficient, or a non-validated observation scheme was adopted. In some studies, physical behavior of toddlers was assessed using observation schemes developed and validated in preschoolers, thereby disregarding developmental specific physical behavior. Thus, physical activities and their intensities may have been misinterpreted. In addition, an epoch length < 60 s is preferred to measure the sporadic and intermittent nature of physical behavior in young children. However, some studies reintegrated the epochs up to three minutes without providing a valid reason, e.g., to align the epochs with the comparison instrument. For studies evaluating validity of cut-points based methods, low methodological study quality was mostly due to validation using data derived under the same circumstances as for the cut-point calibration, e.g., using the same sample instead of an independent sample. Studies that assessed validity of multi-parameter methods, mainly received low methodological study quality due to not reporting statistics suitable for comparing the performance of prediction models.

Next to the low quality of evidence, there were some general study limitations. For instance, in the reliability studies, differences between accelerometer recordings might be due to slightly different placement of the accelerometer, or actual different physical behaviors during the repeated measurements. Differences based on mechanical shaker experiments, on the other hand, are purely device related. Regarding validity studies using observation as comparison measure, it is difficult to estimate what observation time would be sufficient for validation of the targeted physical intensities and representative for physical behavior. This is also dependent on the observed activities and setting. Observation durations of included studies varied from 8 to 180 min. For instance, if observation took place in the childcare setting, this might not be representative for daily life physical behavior. Another general limitation is that activity types were not described in detail, resulting in different activity intensity ranges between studies.

### Strengths and limitations

A strength of this review is that the methodological study quality was systematically assessed using the newly developed CAMQAM checklist. Another strength is that our search had no publication date limit. Although this resulted in including studies on devices that are no longer available on the market, this review includes all available studies examining measurement properties of accelerometer-based methods in 0—5-year-olds. A limitation of this review is that due to the great variability of accelerometer-based methods, it was not feasible to pool the study results, resulting in low quality of evidence ratings due to small sample sizes. Appropriate sample sizes are important for precision but also in order to capture adequate variation in physical activities. Another limitation is that we were unable to rate study results of measurement errors, as the minimal important difference or minimal important change is needed to conclude on the magnitude of measurement error. Since this information was not available for 0—5-year-olds, we only described these results. A limitation of the study result rating is that, the ratings were not weighted for the number of reported results. Lastly, our focus was limited to the evaluation and quality of measurement properties and did not include feasibility. Feasibility is context-specific because studies differ in available expertise and computational resources to perform the data analysis. Further, feasibility may change over time as software is subject to ongoing development and maintenance, or lack thereof, and accelerometers may change in price or dimensions as newer models enter the market.

### Recommendations for future studies

High quality studies and standardized protocols are required to assess measurement properties (including feasibility) of these accelerometer-based methods and enable pooling of data. To improve methodological study quality of future studies, we recommend using our developed CAMQAM Checklist for Assessing the Methodological Quality of studies using Accelerometer-based Methods. Future studies should incorporate more precise definitions for physical activity types, adapted to the child’s developmental stage. For example, activity types such as crawling can be more precisely defined using inclination angles in video observation or derived from accelerometer-based methods. Additionally, for accelerometer-based methods to be generalizable to young children, ideally in a free-living setting, validation studies should include a variety of physical activity types representative for the target population. Moreover, we recommend that future studies transparently share methods by making these open-source available. Making methods accessible supports the sustained impact of research investments. Given the lack of reliable and/or valid of accelerometer-based methods and the lack of 24-h studies on physical behavior, especially in the youngest age groups (i.e., infants and toddlers), future studies should develop and evaluate methods targeted at these young age groups, including all 24-h physical behaviors, and exploring different sensor placements and axes using raw acceleration data of modern accelerometers.

## Conclusions

Validated cut-points are lacking in infants and toddlers, while multi-parameter methods proved valid to distinguish SB and LPA from more vigorous activities. For preschoolers, both valid cut-points based and valid multi-parameter methods were identified, where multi-parameter methods appeared to have better measurement properties. Large heterogeneity and methodological limitations, impedes our ability to draw definitive conclusions about the best available accelerometer-based methods assessing all 24-h physical behaviors combined in young children.

## Supplementary Information

**Additional file 1.** Search strategy using MEDLINE for studies evaluating accelerometer-based methods.

**Additional file 2.** CAMQAM: Checklist for Assessing the Methodological Quality of studies using Accelerometer-based Methods.

**Additional file 3.** Description of the elements and the corresponding codes to describe accelerometer-based methods.

## Data Availability

The data that support the findings of this review are available from the corresponding author upon reasonable request.
