# Supplementary material for: Systematic review of accelerometer-based methods for 24-h physical behavior assessment in young children (0–5 years old)
Source: Int J Behav Nutr Phys Act. 2022 Sep 8;19:116. doi: 10.1186/s12966-022-01296-y (PMC9461103; doi:10.1186/s12966-022-01296-y)
Supplement: Supplementary file 1 — Additional file 1. Search strategy using MEDLINE for studies evaluating accelerometer-based methods. [file 12966_2022_1296_MOESM1_ESM.pdf]

Additional File 1 – Search strategy using MEDLINE for studies evaluating accelerometer-based methods

Search strategy using MEDLINE for studies evaluating accelerometer-based methods

Systematic review of accelerometer-based methods for 24-hour physical behavior assessment in young children (0–5 years old).

Journal: International Journal of Behavioral Nutrition and Physical Activity

Annelinde Lettink, Teatske M. Altenburg, Jelle Arts, Vincent T. van Hees, & Mai. J. M. Chinapaw

Corresponding author:
Annelinde Lettink
1. Amsterdam UMC location Vrije Universiteit Amsterdam, Public and Occupational Health, De Boelelaan 1117, Amsterdam, The Netherlands
2. Amsterdam Public Health, Methodology and Health Behavior & Chronic Diseases, Amsterdam, The Netherlands
E-mail: a.lettink@amsterdamumc.nl

Search strategy using MEDLINE for studies evaluating accelerometer-based methods

#1	((("Motor Activity"[Mesh:NoExp] OR "Exercise"[Mesh] OR "Sports"[Mesh] OR motor activit*[tiab] OR physical activit*[tiab] OR locomotor activit*[tiab] OR exercis*[tiab] OR running[tiab] OR run[tiab] or sport*[tiab] OR active[tiab] OR walk*[tiab] OR outdoor[tiab] OR play*[tiab] OR bicycle[tiab] OR cycle[tiab] OR bicycling[tiab] OR cycling[tiab] OR biking[tiab] OR "tummy time"[tiab] OR "floor time"[tiab] OR "prone position"[tiab] OR crawl*[tiab] OR swim*[tiab] OR "rough and tumble"[tiab] OR "Movement"[MeSH:NoExp] OR movement[tiab])) OR ("Sedentary Behavior"[Mesh:NoExp] OR sedentary[tiab] OR stationary behavio*[tiab] OR physically inactive[tiab] OR physical inactiv*[tiab] OR sitting[tiab] OR computer time[tiab] OR computer use[tiab] OR screen time[tiab] OR television[tiab] OR "TV time"[tiab] OR "TV viewing"[tiab] OR "watching TV"[tiab] OR videogam*[tiab] OR gaming[tiab] OR tablet use[tiab] OR "tablet time"[tiab] OR screen entertainment[tiab] OR screen based[tiab])) OR ("Sleep"[Mesh:NoExp] OR sleep*[tiab] OR time in bed[tiab] OR bed time[tiab] OR bedtime[tiab] OR night rest[tiab] OR night awakening*[tiab] OR night waking*[tiab] OR nap*[tiab])
#2	"Infant"[Mesh:noexp] OR "Infant, Newborn"[Mesh:noexp] OR "Child, Preschool"[Mesh] OR infan*[tiab] OR newborn*[tiab] OR new-born*[tiab] OR neonate*[tiab] OR baby[tiab] OR babies[tiab] OR toddler*[tiab] OR preschool*[tiab] OR pre-school*[tiab] OR kindergarten*[tiab] OR childcare*[tiab] OR daycare*[tiab] OR nurser*[tiab] OR ECEC[tiab] OR early childhood[tiab] OR early years[tiab] OR early life[tiab] OR children[tiab]
#3	((instrumentation[sh] OR methods[sh] OR “Validation Studies”[pt] OR Comparative Study[pt] OR Evaluation Study[pt] OR "psychometrics"[MeSH] OR psychometr*[tiab] OR clinimetr*[tw] OR clinometr*[tw] OR "reproducibility of results"[MeSH] OR reproducib*[tiab] OR "discriminant analysis"[MeSH] OR reliab*[tiab] OR unreliab*[tiab] OR valid*[tiab] OR coefficient[tiab] OR agreement[tw] OR precision[tw] OR imprecision[tw] OR "precise values"[tw] OR test-retest[tiab] OR (test[tiab] AND retest[tiab]) OR (reliab*[tiab] AND (test[tiab] OR retest[tiab])) OR stability[tiab] OR kappa[tiab] OR kappa's[tiab] OR kappas[tiab] OR repeatab*[tw] OR replicab*[tw] OR repeated[tw] OR concordance[tiab] OR (intraclass[tiab] AND correlation*[tiab]) OR discriminative[tiab] OR "known group"[tiab] OR (variability[tiab] AND (analysis[tiab] OR values[tiab])) OR (uncertainty[tiab] AND (measurement[tiab] OR measuring[tiab])) OR "standard error of measurement"[tiab] OR "Sensitivity and Specificity"[MeSH] OR sensitiv*[tiab] OR specific*[tiab] OR responsive *[tiab] OR accura*[tiab] OR "golden standard" OR "gold standard" OR reference[tiab] OR roc[tiab] OR reference values[mesh] OR cutoff[tiab] OR cut-off[tiab] OR repeatability[tiab] OR suitability[tiab] OR utility[tiab])) AND ("Accelerometry/methods"[Mesh] OR acceleromet*[tiab] OR actigraph*[tiab])
#4	#1 AND #2 AND #3
#5	#4 NOT ("Diseases Category"[Majr] OR "Attention Deficit and Disruptive Behavior Disorders"[Majr] OR "Child Development Disorders, Pervasive"[Majr])
#6	#5 NOT ("Animals"[Mesh] NOT "Humans"[Mesh])
