# Supplementary material for: Systematic review of accelerometer-based methods for 24-h physical behavior assessment in young children (0–5 years old)
Source: Int J Behav Nutr Phys Act. 2022 Sep 8;19:116. doi: 10.1186/s12966-022-01296-y (PMC9461103; doi:10.1186/s12966-022-01296-y)
Supplement: Supplementary file 2 — Additional file 2. CAMQAM: Checklist for Assessing the Methodological Quality of studies using Accelerometer-based Methods. [file 12966_2022_1296_MOESM2_ESM.pdf]

Additional File 2 – CAMQAM: Checklist for Assessing the Methodological Quality of studies using Accelerometer-based Methods

CAMQAM: Checklist for Assessing the Methodological Quality of studies using Accelerometer-based Methods

Systematic review of accelerometer-based methods for 24-hour physical behavior assessment in young children (0–5 years old).

Journal: International Journal of Behavioral Nutrition and Physical Activity

Annelinde Lettink, Teatske M. Altenburg, Jelle Arts, Vincent T. van Hees, & Mai. J. M. Chinapaw

Corresponding author:
Annelinde Lettink
1. Amsterdam UMC location Vrije Universiteit Amsterdam, Public and Occupational Health, De Boelelaan 1117, Amsterdam, The Netherlands
2. Amsterdam Public Health, Methodology and Health Behavior & Chronic Diseases, Amsterdam, The Netherlands
E-mail: a.lettink@amsterdamumc.nl

CAMQAM: Checklist for Assessing the Methodological Quality of studies using Accelerometer-based Methods

This supplement presents the newly developed Checklist for assessing the Methodological Quality of studies using Accelerometer-based Methods (CAMQAM) and provides the scoring manual. We distinguish two measurement properties: reliability and validity. Each measurement property includes different types, with specific quality aspects.
Table 1 summarizes the definitions of the measurement properties of studies evaluating accelerometer-based methods and preferred methods for evaluation.

Table 1.

Summary of the definitions and preferred methods for evaluation of the measurement properties[a]

Measurement property	Definition	Preferred method
1. Reliability	The degree to which a measurement instrument is free from measurement error [1]	Study design: at least two measurements; independent measurements; similar measurement conditions; appropriate time interval
a) Test-retest reliability	The degree to which scores collected from the same participant are the same for repeated measurements	Statistical method: Intraclass correlation coefficient (*ICC*) in accordance with model, type and definition [2], or $\kappa_{(w)}$
b) Inter-device reliability	The degree to which scores vary that are simultaneously collected using multiple devices at the same placement site	Statistical method: *ICC* in accordance with model, type and definition [2], or correlation (r_p, r_{sp}, or r)
2. Validity	The degree to which an instrument truly measures the construct it purports to measure [3]	
a) Criterion validity	The degree to which the scores of an instrument are an adequate reflection of a gold standard	Study design: criterion measure polysomnography or indirect calorimetry<br>Statistical method: correlation or area under the receiver operating curve (*AUC-ROC*) (continuous scores); sensitivity and specificity (dichotomous scores)
b) Convergent validity	The degree to which the scores of a measurement instrument are consistent with other instruments assessing the same construct	Study design: sufficient measurement properties of comparator instrument (e.g., κ > .70)<br>Statistical method: Bland-Altman or $\kappa_{(w)}$ with ICC or limits of agreement (*LoA*)
Validity of a specific data analysis approach		
3.1 Validity of cut-points based methods (using a single value to categorize physical behavior)	The extent to which the newly developed (calibrated) or pre-defined cut-point is able to classify accelerometer-derived data as physical intensities (e.g., sedentary behavior, light physical activity, moderate physical activity, vigorous physical activity) consistently with the comparator instrument(s) (epoch-by-epoch or time spent in physical intensity class)	Study design: calibration interval matches accelerometer epoch length; cross-validation of cut-point in a different sample with similar characteristics<br>Statistical method: Bland-Altman or $\kappa_{(w)}$ with *ICC* or *LoA*
3.2 Validity of multi-parameter methods (using more than one parameter to categorize physical	The extent to which scores of an algorithm or a classifier predict scores on a comparator instrument(s) [4]	Statistical method: accuracy reported for all classes and confidence interval

behavior, e.g., sleep algorithms, machine learning methods)	(or related measure such as precision or recall)

Abbreviations: *ICC* intraclass correlation coefficient, κ Kappa, κ_w weighted Kappa, *r* correlation coefficient (unknown), r_p correlation coefficient (Pearson), r_{sp} correlation coefficient (Spearman's rank), r unknown correlation coefficient, *AUC-ROC* area under the receiver operating curve, *LoA* limits of agreement
a Table adapted from Terwee et al. (2010) [3]

The following sections present the checklist boxes to assess whether a study meets the standards for good methodological study quality and a guide for the appraisal of these measurement properties.

RELIABILITY

Test-retest reliability

This type of reliability of accelerometer-based devices refers to the consistency in the outcome (e.g., physical activity, sedentary behavior, sleep) of accelerometer recordings. An important assumption in test-retest reliability is that the participants wearing the accelerometer-based device are stable in the interim period (i.e., maximum of two weeks) on the construct to be measured between the repeated measurements (rated using items 1-3). Depending on the outcome scores, the preferred statistics to be used are the Intraclass Correlation Coefficient (*ICC*) (in case of continuous scores) in accordance with model, type, and definition [2] or (weighted) Kappa ($\kappa_{(w)}$) (rated using items 4-8). The checklist for assessing test-retest reliability is presented in Box 1a and explained below.

Item 1. Were participants stable in the interim period on the construct to be measured?
Evidence that participants were stable could be, for example, an assessment of a global rating of change (e.g., number of hours sleep), completed by the participants or their caregivers. When an intervention was given, in the interim period, one can assume that (many of) the participants have changed their physical behavior. In that case, it is recommended to rate this item as "Inadequate".

Item 2. Was the time interval appropriate?
The time interval between the accelerometer recordings must be appropriate. It should be short enough to ensure that participants have not substantially changed their physical behavior patterns (e.g., starting to walk, walking with a walker). A time interval of maximal 2 weeks was considered appropriate and rated as "Very good".

Item 3. Were the test conditions similar for the measurements?
The test conditions should be similar. This refers to the type of administration (e.g., the same axis and of the accelerometer was used, the device was placed on the same location), the setting in which the accelerometer-based device was administered (e.g., home, preschool), and the instructions given. The reliability may be underestimated if these test conditions were not similar.

Item 4. For continuous scores: Was an intraclass correlation coefficient (ICC) calculated?
For continuous scores the ICC is preferred because this statistic incorporates systematic error. The use of correlation coefficients (Pearson (r_p) or Spearman's rank (r_{sp})) is rated as "Doubtful" when it is unclear whether there were systematic differences, as these do not incorporate systematic errors. This item was rated as "Very good" if the ICC was calculated, the

model/formula of the ICC was described, and this analysis decision was appropriate according to Koo and Li (2016), e.g., two-way mixed effects model, single measurement or mean of *k* measurements, absolute agreement, or consistency [2].

Items 5, 6, 7. Was $\kappa_{(w)}$ calculated? Was the weighting scheme described? (e.g., linear, quadratic)
For dichotomous or ordinal scores, the Cohen's $\kappa_{(w)}$ is the preferred statistical method, while for ordinal scores partial chance agreement should be considered, and therefore κ should be weighted (i.e., κ_w). In addition, a description of the weights should be included.
Item 8. Were there any other important flaws?
Examples of important flaws are when participants were only included if their data were complete or more than 50% of the data were missing.

Inter-device reliability
This type of reliability refers to the consistency in accelerometer-derived (epoch level) data between different devices. The devices should be attached at the same side of the body, as dominance of side influences the accelerometer recordings. An important assumption in inter-device reliability is that device settings such as epoch length and sampling frequency between the devices were similar (rated using item 1). Depending on the type of score, the preferred statistics to be used are the *ICC* (in case of continuous scores) in accordance with model, type, and definition [2] or $\kappa_{(w)}$ (rated using items 4-8). The checklist for assessing test-retest reliability is presented in Box 1b and explained below.

Item 1. Were the device settings similar?
The device settings should be similar for a fair comparison. For example, the epoch length, sampling frequency, dynamic range, etc. The reliability may be underestimated if these test conditions were not similar.

Item 2. For continuous scores: Was an *ICC* calculated?
For continuous scores the *ICC* is preferred because this statistic incorporates systematic error. The use of correlation coefficients (r_p or r_{sp}) is rated as "Doubtful" when it is unclear whether there were systematic differences, as these do not incorporate systematic errors. This item was rated as "Very good" if the ICC was calculated, the model/formula of the ICC was described, and this analysis decision was appropriate according to Koo and Li (2016), e.g., one-way/two-way random effects model or two-way mixed effects, single rater or mean of *k* raters, absolute agreement [2].

Item 3, 4, 5. Was $\kappa_{(w)}$ calculated? Was the weighting scheme described? (e.g., linear, quadratic)
For dichotomous or ordinal scores, Cohen's κ is the preferred statistical method, while for ordinal scores partial chance agreement should be considered, and therefore κ should be weighted (i.e., κ_w). In addition, a description of the weights should be included.

Item 6. Were there any other important flaws?
Examples of important flaws are when participants were only included if their data were complete or more than 50% of the data were missing.

Checklist boxes reliability

Box 1a. Test-retest reliability[a]

Item	Very good	Adequate	Doubtful	Inadequate	NA
1) Were participants stable in the interim period on the construct to be measured?	Evidence provided that participants were stable	Assumable that participants were stable	Unclear if participants were stable	Participants were not stable	
2) Was the time interval appropriate? (i.e., maximal 2 weeks)	Time interval appropriate		Doubtful whether time interval was appropriate, or time interval was not stated	Time interval was not appropriate	
3) Were the test conditions similar for the measurements? (e.g., type of administration, environment, instructions)	Test conditions were similar (evidence provided)	Assumable that test conditions were similar	Unclear if test conditions were similar	Test conditions were not similar	
4) For continuous scores: Was an ICC calculated?	*ICC* calculated, and model/formula of the *ICC* is described, and analysis decision was appropriate according to Koo and Li (2016), e.g., one-way/two-way random effects model or two-way mixed effects, single rater or mean of k raters, absolute agreement, or consistency [2]	ICC calculated but model/formula of the ICC not described or not optimal. r_p or r_{sp} calculated with evidence provided that no systematic change has occurred	r_p or r_{sp} calculated without evidence provided that no systematic change has occurred or with evidence that systematic change has occurred	No *ICC*, r_p or r_{sp} calculated	NA
5) For dichotomous/ nominal/ ordinal scores: Was κ calculated?	κ calculated			No κ calculated	NA
6) For ordinal scores: Was a κ_w calculated?	κ_w calculated		κ calculated or not described		NA
7) For ordinal scores: Was the weighting scheme described? (e.g., linear, quadratic)	Weighting scheme described	Weighting scheme not described			NA
8) Were there any other important flaws?	No other important methodological flaws		Other minor methodological flaws	Other important methodological flaws	

Abbreviations: ICC intraclass correlation coefficient, κ Kappa, κ_w weighted Kappa, NA not applicable r_p Pearson correlation coefficient, r_{sp} Spearman's rank correlation coefficient
a Adapted from 'Box 6 Reliability' of the COnsensus-based Standards for the selection of health Measurement INstruments (COSMIN) risk of bias checklist [1, 5, 6]

Box 1b. Inter-device reliability[a]					
**Item**	**Very good**	**Adequate**	**Doubtful**	**Inadequate**	**NA**
1) Were the device settings similar? (e.g., epoch length, sampling frequency)	Device settings were similar (evidence provided)	Assumable that device settings were similar	Unclear if device settings were similar	Device settings were not similar	
2) For continuous scores: Was an *ICC* calculated?	*ICC* calculated, and model/formula of the *ICC* is described, and analysis decision was appropriate according to Koo and Li (2016), e.g., two-way mixed effects model, single measurement or mean of k measurements, absolute agreement [2]	*ICC* calculated but model/formula of the *ICC* not described or not optimal. r_p or r_{sp} calculated with evidence provided that no systematic change has occurred	r_p or r_{sp} calculated without evidence provided that no systematic change has occurred or with evidence that systematic change has occurred	No *ICC*, r_p or r_{sp} calculated	NA
3) For dichotomous/ nominal/ ordinal scores: Was κ calculated?	κ calculated			No κ calculated	NA
4) For ordinal scores: Was a κ_w calculated?	κ_w calculated		κ_w calculated or not described		NA
5) For ordinal scores: Was the weighting scheme described? (e.g. linear, quadratic)	Weighting scheme described	Weighting scheme not described			NA
6) Were there any other important flaws?	No other important methodological flaws		Other minor methodological flaws	Other important methodological flaws	

Abbreviations: ICC intraclass correlation coefficient, κ Kappa, κ_w weighted Kappa, NA not applicable, r_p Pearson correlation coefficient, r_{sp} Spearman's rank correlation coefficient

a Adapted from 'Box 6 Reliability' of the COnsensus-based Standards for the selection of health Measurement INstruments (COSMIN) risk of bias checklist [1, 5, 6]

VALIDITY

Note that: if a study assessed the criterion or convergent validity of a specific data analysis approach (e.g., cut-points based or multi-parameter methods), it is required to rate the applicable additional checkbox items besides rating criterion- or convergent validity depending on the comparator instrument.

Criterion validity

An important assumption in criterion validity is that the best reference methods available (i.e., polysomnography for sleep, indirect calorimetry such as doubly labelled water for total energy expenditure) were considered the gold standard to assess validity of the accelerometer-based method. Although these gold standards are not perfect (e.g., doubly labelled water cannot distinguish between type, frequency, and intensity of activities), these are viewed as the best reference measures available. Depending on the type of score, the preferred statistics to be used area under the receiver operating curve (*AUC-ROC*) (in case of continuous scores) or sensitivity and specificity (rated using items 1-2). The checklist for assessing criterion validity is presented in Box 2a and explained below.

Items 1, 2. Were correlations, or the *AUC-ROC* calculated? Were sensitivity and specificity determined?

When both the accelerometer-derived data and gold standard were analyzed as continuous scores, correlations are the preferred statistical method. When the accelerometer-derived data was analyzed as continuous and the gold standard as dichotomous the *AUC-ROC* is the preferred statistical method. When both the accelerometer-derived data and gold standard were analyzed as dichotomous scores, sensitivity and specificity are the preferred statistical methods. Note, that if the accelerometer-derived data was analyzed using a multi-parameter method and model scores were reported, item 1 was scored as “NA”, as the statistical analyses of the specific data analysis approach were further rated in the applicable additional checkbox.

Item 3. Were there any other important flaws?

The most important flaw for measuring physical behavior is related to the epoch length, as longer epochs are more insensitive to detect changes in type and intensity of physical activity, as well as intermittent behaviors. If the epoch length was not < 60 s for studies that examined validity of an accelerometer-based method for assessment of physical activity without a plausible reason, such as alignment with gold standard, this item was rated as “Doubtful”. Another example of a flaw is not calculating a 95% confidence interval for *AUC-ROC* values.

Convergent validity

If another comparator instrument was used than a “gold standard” to evaluate validity, convergent validity was evaluated. For measuring convergent validity of accelerometer-based methods, typical comparator instruments are direct observation or other accelerometer-based methods (including different device types or analysis approach). An important assumption of convergent validity is that the comparator instrument has sufficient measurement properties (rated using items 1-2). Additionally, it is of importance that studies not only addressed the agreement between the two methods (e.g., correlation) but also evaluated disagreement between the two methods (rated using item 3). The checklist for assessing convergent validity is presented in Box 2b and explained below.

Items 1, 2. Is it clear what the comparator instrument(s) measure(s)? Were the measurement properties of the comparator instrument(s) adequate?
These comparator instruments are required to be explained in detail. For example, if the observation scheme was provided and explained, it is recommended to rate item 1 as “Very good”. If the observation scheme was not presented or referred to, this item is scored as “Inadequate”. In addition, the measurement properties of this comparator instrument need to be sufficient (e.g., $\kappa > .70$), preferably tested in a population similar to the study population. When the comparator instrument was an accelerometer-based device that used orientation classification (thigh data), e.g., activPAL, measuring moderate physical activity, vigorous physical activity, or moderate-to-vigorous physical activity, it is recommended to rate item 2 as “Doubtful”, because posture is registered. If this accelerometer-based device that used orientation classification to assess sedentary behavior, item 2 can be rated as “Very good”. If an observation scheme was used and interrater agreement was sufficient (i.e., $\kappa > .70$), this item can be rated as “Very good”.

Item 3. Was the statistical method appropriate for the hypotheses to be tested?
As the comparator instrument used is not considered a “gold standard”, the study is required to not only address the agreement between the accelerometer-based method and the comparator instrument (e.g., correlation) as well as disagreement between the two methods (i.e., preferably Bland-Altman plots with *LoA*) [7]. Note, that if additional scoring for the analysis approach is required (e.g., convergent validity of the cut-points based method or multi-parameter method is evaluated) this item can be scored as “NA”, because the statistical approach is rated in the applicable additional checkbox.

Item 4. Were there any other important flaws?
This item is similar as item 3 for criterion validity.

Validity Checkboxes

Box 2a. Criterion validity[a]					
**Item**	**Very good**	**Adequate**	**Doubtful**	**Inadequate**	**NA**
1) For continuous scores: Were correlations, or the AUC-ROC calculated?	Correlations or *AUC-ROC* calculated			Correlations or *AUC-ROC* not calculated	Model scores were calculated (e.g. RMSE) AND the validity of a cut-points based or mult-parameter method was rated
2) For dichotomous scores: Were sensitivity and specificity determined?	Sensitivity and specificity calculated			Sensitivity and specificity not calculated	
3) Were there any other important flaws?	No other important methodological flaws		Other minor methodological flaws	Other important methodological flaws	

Abbreviations: AUC-ROC area under the receiver operating curve, NA not applicable
a Adapted from 'Box 8 Criterion Validity' of the COnsensus-based Standards for the selection of health Measurement INstruments (COSMIN) risk of bias checklist [1, 5, 6]

Box 2b. Convergent validity[a]					
**Item**	**Very good**	**Adequate**	**Doubtful**	**Inadequate**	**NA**
1) Is it clear what the comparator instrument(s) measure(s)?	Construct measured by the comparator instrument(s) is clear			Constructs measured by the comparator instrument(s) is not clear	
2) Were the measurement properties of the comparator instrument(s) adequate?	Sufficient measurement properties of the comparator instrument(s) (e.g., $\kappa > .70$) and in a population similar to the study population (e.g., accelerometer-based orientation classification for assessing SB)	Sufficient measurement properties of the comparator instrument(s) (e.g., $\kappa > .70$) and not sure if these apply to a similar study population, or moderate measurement properties of the	Some information on measurement properties of the comparator instrument(s) in any study population (e.g., accelerometer-based orientation classification	No information on the measurement properties of the comparator instrument(s), or evidence for insufficient measurement properties of the comparator instrument(s)	

		comparator instrument(s) (e.g., $\kappa \geq .50$) and in a population similar to the study population	for assessing MPA, VPA or MVPA)		
3) Was the statistical method appropriate for the hypotheses to be tested?	Statistical method was appropriate (e.g., Bland Altman plots or κ and estimation of the *ICC* and/or *LoA*	Assumable that statistical method was appropriate (e.g., using only Bland-Altman plots or κ)	Statistical method applied not optimal (e.g., only % agreement or correlation)	Statistical method applied not appropriate	The validity of a cut-points based or multi-parameter method was also rated
4) Were there any other important flaws?	No other important methodological flaws		Other minor methodological flaws (e.g., only data presented on a comparison with an instrument that measures another construct)	Other important methodological flaws	

Abbreviations: ICC intraclass correlation coefficient, κ Kappa, *LoA* limits of agreement, MPA moderate physical activity, MVPA moderate-to-vigorous physical activity, NA not applicable, SB sedentary behavior, VPA vigorous physical activity

a Adapted from ‘Box 9 Hypothesis testing for construct validity’ part ‘9a Comparison with other outcome measurement instruments’ of the COnsensus-based Standards for the selection of health Measurement INstruments (COSMIN) risk of bias checklist [1, 5, 6]

Validity of a specific data analysis approach

Depending on the specific data analysis approach, the following box was rated: Box 3.1 for conventional cut-points based methods using one parameter for physical behavior categorization, or Box 3.2 for multi-parameter methods using more than one parameter to categorize physical behavior.

Validity of cut-points based methods

Studies that used a comparator instrument to evaluate the validity of cut-points based methods were additionally assessed on the statistical method used for analyses (rated using item 1), the calibration (rated using item 2-3) and validation (rated using item 4) procedures. The additional items applicable for assessing criterion- or convergent validity of cut-points based methods are presented in Box 3.1 and explained below.

Item 1. Was the statistical method appropriate for examining agreement between activity magnitude/acceleration (e.g., accelerometer counts) and the comparator instrument?

Ideally, *AUC-ROC* analysis is used to calibrate the cut-point, maximizing sensitivity (e.g., the % of epochs classified as sedentary behavior that were classified by the comparator instrument as sedentary behavior) and specificity (e.g., the % of epochs classified as not sedentary behavior that were classified by the comparator instrument as not sedentary behavior). Then, for the assessment of validity in the independent sample statistical models (e.g., non-parametric or regression models) or Bland–Altman methods[7] may be used in addition to, or alternatively to, *AUC-ROC* methods, to examine relationships or agreement between the accelerometer-based method and the comparator measure. If the study only considers percentage agreement it is recommended to rate this item as “Doubtful”. If only correlation is considered it is recommended to rate this item as “Inadequate”, because here only the association is incorporated, while the disagreement is completely disregarded.

Item 2. Was cut-point calibration performed on data derived under different circumstances than the validation?

When for instance, the calibration procedure is performed on data derived from structured observation and the validation procedure on free-play or unstructured observation this item can be scored as “Very good”. If the calibration is performed on data derived under the same circumstances it is recommended to score this item as “Doubtful”, as this may result in overfitting.

Item 3. Were the calibration intervals matched with the accelerometer epoch lengths?

If the cut-point from previous studies were applied this item was scored as “NA”. However, when observation is used as comparator instrument, it is of importance that the calibration intervals are sufficiently matched with the accelerometer epoch length. It is recommended to score this item as “Very good” when the study matches the observation intervals and the epoch length before the data collection. In addition, when there is a valid reason for matching the epoch length after data collection this item can also be scored as “Very good”. If not, it is recommended to score this item as “Inadequate”.

Item 4. Was the validation method of the study adequate?

Ideally, validation was performed in a different sample with comparable characteristics (BMI, age, gender, race, height, width), or the data were split into a test set and validation set. If a

subsample was used to validate the data it is recommended to score this item as “Doubtful”, because the validation set overlaps with the test set. This item was rated as “Inadequate” when validation was performed on data of same sample without cross-validation or repeated measures.

Item 5. Were there any other important flaws?
This item is similar as item 3 for criterion validity.

Validity of multi-parameter methods
When using multi-parameter methods for analysis of accelerometer data, typically classifiers, machine learning methods, or algorithms are developed to predict activity type, activity intensity, or sleep-wake states. For validity of these methods, it is of importance that insight is provided in the classification process (rated using item 1). If the performance of multiple algorithms or predictors were compared, reporting of comparable values is of importance for distinguishing the best predictor or classification algorithm (rated using item 2). The additional items for assessing criterion- or convergent validity of multi-parameter methods are presented in Box 3.2 and explained below.

Item 1. Were prediction results reported in an adequate way?
Ideally, complete confusion matrices (or related measures) for all activity classes were reported including a confidence interval. In this way insight is provided for both correct and incorrect predictions. It is recommended to rate this item as “Inadequate” when only total accuracy of the method was provided. If only one activity type was predicted and total accuracy was reported, this item can be rated as “Very good”. In case of energy expenditure prediction, it is recommended to rate this item as ‘’Very good’’ when closeness of the predicted values and the observed values were reported as the model fit, e.g., RMSE.

Item 2. Were predictions of the methods compared in an adequate way?
Ideally, the study compares multiple methods statistically using goodness of fit measures that control for the number of parameters in the models, e.g., AIC, BIC. If the study reported statistics suitable for comparison (e.g., κ) but do not control for the number of parameters, it is recommended to rate this item as “Adequate”.
However, if these models were nested models (i.e., model A is nested in model B if the parameters in model A are a subset of the parameters in model B) and the results were not compared, this item is recommended to be rated as “Doubtful”. It is recommended to rate this item as “Inadequate” when the results of multiple methods were not statistically compared.

Item 3. Were there any other important flaws?
An example of other flaws is low interrater agreement to derive annotated labels for prediction.

Additional Checkboxes

Box 3.1. Validity of cut-points based methods[a]

Item	Very good	Adequate	Doubtful	Inadequate	NA
1) Was the statistical method appropriate for examining agreement between activity magnitude/acceleration (e.g., accelerometer counts) and the comparator instrument?	Using Bland-Altman plots or κ and estimation of the *ICC* and/or *LoA*, or *AUC-ROC* analysis	Using Bland-Altman plots, κ, sensitivity or specificity	Only % agreement	Correlation	
2) For studies using observation as comparator instrument: Was cut-point calibration performed on data derived under different circumstances than the validation?	Yes			No	Comparator instrument other than observation, or pre-defined cut-points were validated
3) For studies using observation as comparator instrument: Were the calibration intervals matched with the accelerometer epoch lengths?	Yes			No	Comparator instrument other than observation, or pre-defined cut-points were validated
4) Was the validation method of the study adequate?	Ideally, in a different sample with comparable characteristics (BMI, age, gender, race, height, width), or test/validation set	k-fold cross-validation was performed	Leave-one out cross-validation was performed / repeated measures on data derived in the same sample	Validation was performed on data derived in the same sample without cross-validation or repeated measures	
5) Were there any other important flaws?	No other important methodological flaws		Other minor methodological flaws (e.g., only data presented on a comparison with an instrument that measures another construct)	Other important methodological flaws	

Abbreviations: AUC-ROC area under the receiver operating curve, BMI body mass index, *ICC* intraclass correlation coefficient, κ Kappa, *LoA* level of agreement, NA not applicable

a Inspired by the COnsensus-based Standards for the selection of health Measurement INstruments (COSMIN) risk of bias checklist [1, 5, 6]

Box 3.2. Validity of multi-parameter methods					
**Item**	**Very good**	**Adequate**	**Doubtful**	**Inadequate**	**NA**
1) Were prediction results reported in an adequate way?	Results were reported for all activity classes including a *CI* and the complete confusion matrix was reported, or other measures related to the confusion matrix (i.e., *precision*, *recall*, *F1-score*)<br>In case of prediction of one activity class: overall *accuracy* was reported<br>In case of EE: model fit was reported (e.g., *RMSE*)	Results were reported for all activity classes, but only a part of the confusion matrix was reported (*FPR*, *FNR*, *TPR*, *TNR*)		Results were reported for all activity classes combined (e.g., overall *accuracy* was reported only)	
2) For comparison of multiple methods: Were predictions of the methods (e.g., machine learning methods, algorithms) compared in an adequate way?	Yes, there was controlled for the number of parameters in the model (e.g., goodness of fit was based on *AIC* or *BIC*)	The results of multiple methods were reported using statistics suitable for comparison (e.g., using κ), but do not control for the number of parameters in the models	The results of multiple methods were not compared but the models were nested (i.e., model A is nested in model B if parameters in model A are a subset of the parameters in model B)	The results of multiple methods were not compared and the models were not nested	Only one method was applied
3) Were there any other important flaws?	No other important methodological flaws		Other minor methodological flaws	Other important methodological flaws	

Abbreviations: AIC Akaike information criterion, *BIC* Bayesian information criterion, *CI* confidence interval, EE energy expenditure, *FNR* false negative rate, *FPR* false positive rate κ Kappa, NA not applicable, *RMSE* root mean squared error, *TNR* true negative rate, *TPR* true positive rate

a Inspired by the COnsensus-based Standards for the selection of health Measurement INstruments (COSMIN) risk of bias checklist [1, 5, 6]

REFERENCES

1. Mokkink LB, De Vet HC, Prinsen CA, Patrick DL, Alonso J, Bouter LM, et al. COSMIN risk of bias checklist for systematic reviews of patient-reported outcome measures. Qual Life Res. 2018;27(5):1171-9.https://doi.org/10.1007/s11136-017-1765-4
2. Koo TK, Li MY. A guideline of selecting and reporting intraclass correlation coefficients for reliability research. J Chiroprac Med. 2016;15(2):155-63.https://doi.org/10.1016/j.jcm.2016.02.012
3. Terwee CB, Mokkink LB, van Poppel MN, Chinapaw MJ, van Mechelen W, de Vet HC. Qualitative attributes and measurement properties of physical activity questionnaires. Sports Med. 2010;40(7):525-37.https://doi.org/10.2165/11531370-000000000-00000
4. Smits N, van der Ark LA, Conijn JM. Measurement versus prediction in the construction of patient-reported outcome questionnaires: can we have our cake and eat it? Qual Life Res. 2018;27(7):1673-82.https://doi.org/10.1007/s11136-017-1720-4
5. Prinsen CA, Mokkink LB, Bouter LM, Alonso J, Patrick DL, De Vet HC, et al. COSMIN guideline for systematic reviews of patient-reported outcome measures. Qual Life Res. 2018;27(5):1147-57.https://doi.org/10.1007/s11136-018-1798-3
6. Terwee CB, Prinsen CA, Chiarotto A, Westerman MJ, Patrick DL, Alonso J, et al. COSMIN methodology for evaluating the content validity of patient-reported outcome measures: a Delphi study. Qual Life Res. 2018;27(5):1159-70.https://doi.org/10.1007/s11136-018-1829-0
7. Hamilton C, Stamey J. Using Bland-Altman to assess agreement between two medical devices--don't forget the confidence intervals! J Clin Monit Comput. 2007;21(6):331-3.https://doi.org/10.1007/s10877-007-9092-x
