# Supplementary material for: Systematic review of accelerometer-based methods for 24-h physical behavior assessment in young children (0–5 years old)
Source: Int J Behav Nutr Phys Act. 2022 Sep 8;19:116. doi: 10.1186/s12966-022-01296-y (PMC9461103; doi:10.1186/s12966-022-01296-y)
Supplement: Supplementary file 3 — Additional file 3. Description of the elements and the corresponding codes to describe accelerometer-based methods. [file 12966_2022_1296_MOESM3_ESM.pdf]

Additional File 3 – Description of the elements and the corresponding codes to describe accelerometer-based methods

Description of the elements and the corresponding codes to describe accelerometer-based methods

Systematic review of accelerometer-based methods for 24-hour physical behavior assessment in young children (0–5 years old).

Journal: International Journal of Behavioral Nutrition and Physical Activity

Annelinde Lettink, Teatske M. Altenburg, Jelle Arts, Vincent T. van Hees, & Mai. J. M. Chinapaw

Corresponding author:
Annelinde Lettink
1. Amsterdam UMC location Vrije Universiteit Amsterdam, Public and Occupational Health, De Boelelaan 1117, Amsterdam, The Netherlands
2. Amsterdam Public Health, Methodology and Health Behavior & Chronic Diseases, Amsterdam, The Netherlands
E-mail: a.lettink@amsterdamumc.nl

Table 1.

Description of the four elements and their corresponding codes to describe accelerometer-based methods using code combinations of the following format: brand-approach-axis-epoch length

Element		Code
1) Brand	ActiGraph (wGT3X-BT, GT3X+, GT3X, GT1M, 7164)	AG
	Actiwatch (AW-64, Mini, AW-L, AW-2)	Aw
	Actical	Ac
	Actiheart	Ah
	activPAL	aP
	AMA-32	AMA
	Axivity (AX3)	Ax
	Caltrac Personal Activity Computer	CPAC
	Computer Science and Applications (WAM 7164) Activity Monitor	CSA
	ICM20600 chip	ICM
	Research Tracker 3	RT
	Gaehwiler Z80-32kV$_1$	Gw
	GENEActiv	GA
	Get Around Garment with ADXL335	GG
	LSI Moving Activity monitor	LSI
	MonBaby	MB
	MicroMini Sleep Watch	MM
	Miniature Semiconductor Chip Accelerometer (brand not specified)	mSCA
	Opal APDM	Op
	Tracmor$_D$	T$_D$
	Suunto Movesense sensor	SM
2) Approach	**TIME SERIES DATA**	
	Activity magnitude	cts
	**CUT-POINTS BASED METHOD**	

				Body position/Activity intensity/Activity type/Sleep stage						
INFANTS	***Name***	***Placement***	***Axis***	*Prone*	*Prone supported*		*Non-prone*		***Unit***	
	Hewitt et al. (2019) [1]	Hip	HA & VA	HA > 0.7 & VA > - 0.1	HA > 0.7 & VA < - 0.1				*g*	He
		Ankle	HA & DA	HA > 0.35 & DA > - 0.45					*g*	
		Chest	VA, HA & DA				< 134		°	
			DA	< -0.1	< -0.1				*G*	
				Prone	*Supine*	*Reclined*	*Upright*	*Inclined*	***Unit***	

	Greenspan, Cunha, & Lobo (2021) [2]	Trunk	VA, HA & DA	< 30	150 - 180	115 – 150	55 – 115	30 - 55	°	Gr
TODDLERS	***Name***	***Placement***	***Axis***	*SB*	*LPA*	*MPA*	*VPA*	*MVPA*	***Unit***	
	Costa et al. (2014) [3]	Right hip	VM	≤ 96.12	96.13 – 361.93			≥ 361.94	cts/5 s	C
			VA	≤ 5	6 – 164			≥ 165		
	Hager et al. (2014) [4]	Left ankle	Omni	≤ 20	21 – 1,099			≥ 1,100	cts/30 s	Ha
	Kelly et al. (2016) [5]	Right hip	VA	≤ 181	182 – 434				cts/15 s	K
	Oftedal et al. (2014) [6]	Waist	VM	< 40					cts/5 s	O
			VA	< 2						
	Pulakka et al. (2013) [7]	Right hip	VM					≥ 208	cts/15 s	Pul
	Trost et al. (2012) [8]	Right hip	VA	≤ 48	49 – 417			≥ 418	cts/15 s	T12
PRESCHOOLERS	***Name***	***Placement***	***Axis***	*SB*	*LPA*	*MPA*	*VPA*	*MVPA*	***Unit***	
	Adolph et al. (2012) [9]	Right hip	VM	≤ 175	176 – 1,399			≥ 1,100	cpm	A
			Omni	≤ 25	26 – 1,149			≥ 1,150		
		Chest	HA	≤ 15	16 – 199			≥ 100		
	Butte et al. (2014) [10]	Right hip	VM	≤ 820	821 – 2,829	2,830 – 6,281	≥ 6,282		cpm	B
			HA	≤ 240	241 – 1,169	1,170 – 3,799	≥ 3,800			
		Chest	HA	≤ 41	42 – 260	261 – 1,179	≥ 1,180			
	de Bock et al. (2010) [11]	Thorax region	VA	< 46 (boys)<br>< 26 (girls)				> 118 (boys)<br>> 105 (girls)	cts/15 s	dB
	Johansson et al. (2015) [12]	Non-dominant wrist	VM	≤ 221	222 – 279			≥ 730	cts/5 s	J13
			VA	≤ 89	90 – 439			≥ 440		
	Johansson et al. (2016) [13]	Non-dominant wrist	VM	≤ 328	329 – 1,392			≥ 1,393	cts/5 s	J16
			VA	≤ 178	179 – 870			≥ 871		
		Left hip	VM	≤ 105	106 – 511			≥ 512		
			VA	≤ 43	44 – 289			≥ 290		
	Li et al. (2020) [14]	Right hip	VM	≤ 3,406	3,407 – 5,690	5,691 – 6,219	≥ 6,220		cpm	Li_{ROC}
				≤ 5,837	5,838 – 14,020	14,021 – 17,432	≥ 17,433			Li_{ORL}
		Non-dominant wrist		≤ 2,556	2,557 – 7,067	7,068 – 14,535	≥ 14,536			Li_{4k}
	Name	***Placement***	***Axis***	*SB*	*LPA*	*MPA*	*VPA*	*MVPA*	***Unit***	
	Pate et al. (2006) [15]	Right hip	VA	< 38	39 – 419	420 – 841	≥ 842		cts/15 s	Pa
				< 201						Pa2
	Pfeiffer et al. (2006) [16]	Right hip	Omni			≤ 715	≥ 1,411		cts/15 s	Pf

	Reilly et al. (2003) [17]	Right hip	Uni	< 1,100					cpm	Re
	Roscoe et al. (2017) [18]	Dominant wrist	VM	< 5.3	5.3 – 8.6	> 8.6			*g*/s	Ro
		Non-dominant wrist		< 8.1	8.1 – 9.3	> 9.3				
	Sirard et al. (2005) [19]	Right hip	VA						cts/15 s	Si
				≤ 301	302 – 614	615 – 1,230	≥ 1,231			Si3 (3 years)
				≤ 363	364 – 811	812 – 1,234	≥ 1,235			Si4 (4 years)
				≤ 398	399 – 890	891 – 1,254	≥ 1,255			Si5 (5 years)
	Trost et al. (2018) [20]	Non-dominant wrist	VA	≤ 349				≥ 1,284	cts/15 s	T18
			VM	≤ 625				≥ 2,103		
		Right hip	VA	≤ 27				≥ 350		
			VM	≤ 263				≥ 674		
	van Cauwenberghe et al. (2011) [21]	Right hip	VA	≤ 372	373 – 584			≥ 585	cts/15 s	vC

	Name	***Placement***	***Axis***	*SB*	*LPA*	*MPA*	*VPA*	*MVPA*	***Unit***	
OTHER	Ekblom et al. (2012) [22]	Non-dominant wrist	VA	≤ 79	80 – 261	262 – 405	≥ 406		cts/15 s	Ek
	Evenson et al. (2008) [23]	Right hip	VA	≤ 25	26 – 573			≥ 574	cts/15 s	Ev
	NHANES [24]	Right hip	VA	≤ 100					cpm	N
	Puyau et al. (2002) [25]	Hip	VA	≤ 800	800 – 3,199			≥ 3,200	cpm	Puy
	Schaefer et al. (2014) [26]	Wrist	Uni	≤ 40	41 – 2,295	2,296 – 6,815	≥ 6,816		cpm	Sc
	Sun et al. (2008) [27]	Hip						> 413	cts/15 s	Swr
								> 780		Slj

	Name	***Placement***	***Axis***	*Sleep*	*Wake*	***Unit***	
OTHER	Philips Actiware (v.5.52.0003, v.6.0)	Wrist	Uni	≤ .888 * mean	> .888 * mean	cpm	ACTdef
				≤ 40	> 40		ACT40
				≤ 80	> 80		ACT80
	Actiwatch Activity and Sleep Analysis 5 v.5.54 (CamNTech)	Wrist	Uni	≤ 40	> 40	cpm	ASA40
	AlgoSmooth [28]			0 for at least 1.5 min	> 100 for ≥ 2 consecutive min	cpm	AS

Zero-threshold computation [29]			0	> 0	cpm	S0
**MULTI-PARAMETER METHOD**						
Name	***Placement***	***Axis***	***Body position/Activity intensity/Activity type/Sleep stage***		***Unit***	
Acceleration and angular velocity algorithm [30]	Legs	HA, DA, VA	Acceleration and angular velocity pattern to differentiate leg movements from non-infant produced movement or noise, where leg movement was defined as "a movement in which the limb changed position in space by the infant's effort" [30]		m/s^2<br>rad/s	AAV
Activinsights Ltd.		HA & VA	Most likely position from scatterplot with rotation (360°) on HA and elevation (up/down angle) on VA		*g*	Ai
Artificial Neural Network			Typically consisting of 3 layers: input, hidden and output			ANN
Automatic Sleep-Wake scoring algorithm		HA, DA, VA	Scores sleep-wake based on 6 most predictive measures (derived using discriminant function analysis on features) using discriminant function analysis		*g*	ASW
Bayes Net			Probabilistic graphical model representing a set of variables (i.e., body position) and their conditional dependencies via a directed acyclic graph			BN
Convolutional Neural Network		HA, DA, VA	Typically consisting of 3 layers: convolutional layer (i.e., sensor module; low-level feature extraction from individual accelerometers and gyroscopes), pooling layer (i.e., sensor fusion module; fusion of individual sensor-level features to common high-level features), and a fully connected layer (i.e. time series modelling module; temporal modelling of learned high-level features to utilize temporal contextual information for classification of body position/movement)		m/s^2<br>(and °/s for gyroscope)	CNN
Cross-sectional time series [31]		HA, VA, DA	Mixed-regression model with random intercepts and random slopes to predict EE, using gender, weight, height, HA, VA, DA, steps, and position		cts	CSTS
Decision table			Majority classifier			DT
Deep Learning Ensemble Network			Using SOM as first layer followed by MLP as second layer			DLEN
Deep Neural Network			Unsupervised end-to-end deep neural network			DNN
Decision tree (pruned)			The branches of a decision tree contain the logic for the classification of activity types (i.e., data is continually split given input features)			J48
k-Nearest Neighbors			Supervised learning algorithm used for classification. Uses the *k* closest training examples as input and classifies the current data instance as majority class of these inputs			kNN
Linear model			Linear model for the prediction of EE using 30 summary statistics			LM
Logistic regression			Statistical analysis method to predict a data value (i.e., body position) based on prior observations			LR
Mixed linear model			Like LM but accounts for repeated measurements			MLM
Multi-layer perceptron network			Supervised model that consists of input, hidden and output layers in which neurons are fully connected by a set of adjustable parameters: weights			MLP
Multinomial logistic regression			Extension of binary logistic regression to categorize more than two categories using maximum likelihood estimation to evaluate category membership probability			MLR
Multivariate adaptive regression splines [31]		HA, VA, DA	Non-parametric regression model to predict EE, using gender, weight, height, HA, VA, DA, steps, position and 1- and 2-min lag and lead values			MARS

	Naïve Bayes			Probabilistic classifier that classifies the data by maximizing Bayes' theorem under the assumption of predictor independence			NB
	activPAL Professional Research Edition software (v.5.8.2.3)			Software to categorize aP data into the following body positions: sit/lie, stand, and walk			PRE
	Random Forests			Ensembles of decision trees for classification using bootstrapping			RF
	Support vector machine			Maps training instances to points in a multidimensional feature space to construct decision boundaries (hyperplanes) maximizing the distance between instances of different classes			SVM
	Self-Organizing Map			Unsupervised model that takes input in the form of vectors and projects these to a two-dimensional grid (i.e., activation map)			SOM
	Name	***Placement***	***Axis***	*Sleep*	*Wake*	***Unit***	
	Automatic sleep-wake scoring algorithm [32]			$2.414 + 0.363V_{0} – 0.113V_{11} + 0.031V_{-4} + 0.012V_{-5} + 0.022V_{+4} + 0.022V_{+5} > 0.1333$	$2.414 + 0.363V_{0} – 0.113V_{11} + 0.031V_{-4} + 0.012V_{-5} + 0.022V_{+4} + 0.022V_{+5} < 0.1333$	cts	ASW
	Weighted sum activity [33]			0.0033(1.06an4 + 0.54an3 + 0.58an2 + 0.76an1 + 2.3a0 + 0.74a1 + 0.67a2) < 1	0.0033(1.06an4 + 0.54an3 + 0.58an2 + 0.76an1 + 2.3a0 + 0.74a1 + 0.67a2) > 1	cpm	WSC
	Count-scaled algorithm [34]			Similar to WSC, except using count-scaled data: mean of non-zero epochs = 30		cts	CS
	Neural network learning vector quantization						LVQ
	Probability scaled algorithm [35]			7.601 – 0.065MW5 – 1.08NAT- 0.056SD6 – 0.073 ln(ACT) ≥ 0	7.601 – 0.065MW5 – 1.08NAT- 0.056SD6 – 0.073 ln(ACT) < 0	cpm	PS
	Probability scaled algorithm for infants [36]			Exact algorithm is unclear, similar to PS but with higher tolerance for movement			PSinf
**3) Axis**	Diagonal axis, perpendicular axis, axis3, z-axis						DA
	Horizontal axis, axis2, x-axis						HA
	Omnidirectional						omni
	Mean of the three axes						tri
	Uniaxial (and the axis is not further specified)						uni
	Vertical axis, axis1, y-axis						VA
	Vector magnitude = $\sqrt{HA^2 + VA^2 + DA^2}$						VM
**4) Epoch length**	1 s						1
	2 s						2
	4 s						4
	5 s						5
	10 s						10
	15 s						15
	30 s						30
	60 s						60

	5 min	300
	10 min	600
	Not reported	n.r.

Note: The code combination AG-Si-VA-15 denotes the accelerometer-based method that was used was an ActiGraph initialized to collect 15 s epochs using the Sirard cut-points for the vertical axis.
Abbreviations: a0 current minute, a1 following one min, a2 following two min, an1 activity counts from the prior one min, an2 activity counts from the prior two min, an3 activity counts from the prior three min, an4 activity counts from the prior four min, ACT activity counts, cpm counts per minute, cts counts, DA diagonal axis (z-axis), EE energy expenditure, *g* acceleration relative to standard gravity ($1g = 9.80665\ m/s^2$), HA horizontal axis (x-axis), LPA light physical activity, MPA moderate physical activity, MVPA moderate-to-vigorous physical activity, MW5 average number of activity counts during the scored epoch and a window of five epochs preceding and following the score epoch, NAT number of epochs with an activity level of ≥ 50 but < 100 activity counts in an 11-min window, PA physical activity, SD6 standard deviation of the activity counts during the scored epoch and the five preceding epochs, SB sedentary behavior, Uni uniaxial (axis not specified), VA vertical axis (y-axis), VM vector magnitude, VPA vigorous physical activity, V_0 number of epochs with zero activity in the global window (i.e., scored epoch plus the five epochs that precede and follow it), V-4 standard deviation of the average activity count in the scored epoch plus the four preceding epochs, V_{-5} standard deviation of the average activity count in the scored epoch plus the five preceding epochs, V_{+4} standard deviation of the average activity count in the scored epoch plus the four following epochs, V_{+5} standard deviation of the average activity count in the scored epoch plus the five following epochs, V_{11} standard deviation of the average activity count in the global window

REFERENCES

1. Hewitt L, Stanley RM, Cliff D, Okely AD. Objective measurement of tummy time in infants (0-6 months): a validation study. PLoS One. 2019;14(2):e0210977.https://doi.org/10.1371/journal.pone.0210977
2. Greenspan B, Cunha AB, Lobo MA. Design and validation of a smart garment to measure positioning practices of parents with young infants. Infant Behav Dev. 2021;62:101530.https://doi.org/10.1016/j.infbeh.2021.101530
3. Costa S, Barber SE, Cameron N, Clemes SA. Calibration and validation of the ActiGraph GT3X+ in 2-3 year olds. J Sci Med Sport. 2014;17(6):617-22.https://doi.org/10.1016/j.jsams.2013.11.005
4. Hager ER, Gormley CE, Latta LW, Treuth MS, Caulfield LE, Black MM. Toddler physical activity study: laboratory and community studies to evaluate accelerometer validity and correlates. BMC Public Health. 2016;16(6):936.https://doi.org/10.1186/s12889-016-3569-9
5. Kelly LA, Villalpando J, Carney B, Wendt S, Haas R, Ranieri BJ, et al. Development of Actigraph GT1M accelerometer cut-points for young children aged 12-36 months. J Athl Enhanc. 2016;5(4):1-4.https://doi.org/10.4172/2324-9080.1000235
6. Oftedal S, Bell KL, Davies PS, Ware RS, Boyd RN. Validation of accelerometer cut points in toddlers with and without cerebral palsy. Med Sci Sports Exerc. 2014;46(9):1808-15.https://doi.org/10.1249/MSS.0000000000000299
7. Pulakka A, Cheung YB, Ashorn U, Penpraze V, Maleta K, Phuka JC, et al. Feasibility and validity of the ActiGraph GT3X accelerometer in measuring physical activity of Malawian toddlers. Acta Paediatr. 2013;102(12):1192-8.https://doi.org/10.1111/apa.12412
8. Trost SG, Fees BS, Haar SJ, Murray AD, Crowe LK. Identification and validity of accelerometer cut-points for toddlers. Obesity (Silver Spring). 2012;20(11):2317-9.https://doi.org/10.1038/oby.2011.364
9. Adolph AL, Puyau MR, Vohra FA, Nicklas TA, Zakeri IF, Butte NF. Validation of uniaxial and triaxial accelerometers for the assessment of physical activity in preschool children. J Phys Act Health. 2012;9(7):944-53.https://doi.org/10.1123/jpah.9.7.944
10. Butte NF, Wong WW, Lee JS, Adolph AL, Puyau MR, Zakeri IF. Prediction of energy expenditure and physical activity in preschoolers. Med Sci Sports Exerc. 2014;46(6):1216-26.https://doi.org/10.1249/mss.0000000000000209
11. de Bock F, Menze J, Becker S, Litaker D, Fischer J, Seidel I. Combining accelerometry and HR for assessing preschoolers' physical activity. Med Sci Sports Exerc. 2010;42(12):2237-43.https://doi.org/10.1249/MSS.0b013e3181e27b5d
12. Johansson E, Ekelund U, Nero H, Marcus C, Hagströmer M. Calibration and cross-validation of a wrist-worn Actigraph in young preschoolers. Pediatr Obes. 2015;10(1):1-6.https://doi.org/10.1111/j.2047-6310.2013.00213.x
13. Johansson E, Larisch LM, Marcus C, Hagströmer M. Calibration and validation of a wrist- and hip-worn Actigraph accelerometer in 4-year-old children. PLoS One. 2016;11(9):e0162436.https://doi.org/10.1371/journal.pone.0162436
14. Li S, Howard JT, Sosa ET, Cordova A, Parra-Medina D, Yin Z. Calibrating wrist-worn accelerometers for physical activity assessment in preschoolers: machine learning approaches. JMIR Form Res. 2020;4(8):e16727.https://doi.org/10.2196/16727

15. Pate RR, Almeida MJ, McIver KL, Pfeiffer KA, Dowda M. Validation and calibration of an accelerometer in preschool children. Obesity (Silver Spring). 2006;14(11):2000-6.https://doi.org/10.1038/oby.2006.234
16. Pfeiffer KA, McIver KL, Dowda M, Almeida MJ, Pate RR. Validation and calibration of the Actical accelerometer in preschool children. Med Sci Sports Exerc. 2006;38(1):152-7.https://doi.org/10.1249/01.mss.0000183219.44127.e7
17. Reilly JJ, Coyle J, Kelly L, Burke G, Grant S, Paton JY. An objective method for measurement of sedentary behavior in 3- to 4-year olds. Obes Res. 2003;11(10):1155-8.https://doi.org/10.1038/oby.2003.158
18. Roscoe CMP, James RS, Duncan MJ. Calibration of GENEActiv accelerometer wrist cut-points for the assessment of physical activity intensity of preschool aged children. Eur J Pediatr. 2017;176(8):1093-8.https://doi.org/10.1007/s00431-017-2948-2
19. Sirard J, Trost S, Pfeiffer K, Dowda M, Pate R. Calibration and evaluation of an objective measure of physical activity in preschool children. J Phys Act Health. 2005;2(3):345--57.https://doi.org/10.1123/jpah.2.3.345
20. Trost SG, Cliff DP, Ahmadi MN, Tuc NV, Hagenbuchner M. Sensor-enabled activity class recognition in preschoolers: hip versus wrist Data. Med Sci Sports Exerc. 2018;50(3):634-41.https://doi.org/10.1249/MSS.0000000000001460
21. van Cauwenberghe E, Gubbels J, De Bourdeaudhuij I, Cardon G. Feasibility and validity of accelerometer measurements to assess physical activity in toddlers. Int J Behav Nutr Phys Act. 2011;8:67.https://doi.org/10.1186/1479-5868-8-67
22. Ekblom O, Nyberg G, Bak EE, Ekelund U, Marcus C. Validity and comparability of a wrist-worn accelerometer in children. J Phys Act Health. 2012;9(3):389-93.https://doi.org/10.1123/jpah.9.3.389
23. Evenson KR, Catellier DJ, Gill K, Ondrak KS, McMurray RG. Calibration of two objective measures of physical activity for children. J Sports Sci. 2008;26(14):1557-65.https://doi.org/10.1080/02640410802334196
24. Matthews CE, Chen KY, Freedson PS, Buchowski MS, Beech BM, Pate RR, et al. Amount of time spent in sedentary behaviors in the United States, 2003-2004. Am J Epidemiol. 2008;167(7):875-81.https://doi.org/10.1093/aje/kwm390
25. Puyau MR, Adolph AL, Vohra FA, Butte NF. Validation and calibration of physical activity monitors in children. Obes Res. 2002;10(3):150-7.https://doi.org/10.1038/oby.2002.24
26. Schaefer CA, Nigg CR, Hill JO, Brink LA, Browning RC. Establishing and evaluating wrist cutpoints for the GENEActiv accelerometer in youth. Med Sci Sports Exerc. 2014;46(4):826-33.https://doi.org/10.1249/MSS.0000000000000150
27. Sun DX, Schmidt G, Teo-Koh SM. Validation of the RT3 accelerometer for measuring physical activity of children in simulated free-living conditions. Pediatr Exerc Sci. 2008;20(2):181-97.https://doi.org/10.1123/pes.20.2.181
28. Sitnick SL, Goodlin-Jones BL, Anders TF. The use of actigraphy to study sleep disorders in preschoolers: some concerns about detection of nighttime awakenings. Sleep. 2008;31(3):395-401.https://doi.org/10.1093/sleep/31.3.395
29. Redmond DP, Hegge FW. Observations on the design and specification of a wrist-worn human activity monitoring system. Behav Res Methods Instrum Comput. 1985;17(6):659-69.https://doi.org/10.3758/BF03200979

30. Smith BA, Trujillo-Priego IA, Lane CJ, Finley JM, Horak FB. Daily quantity of infant leg movement: wearable sensor algorithm and relationship to walking onset. Sensors (Basel). 2015;15(8):19006-20.https://doi.org/10.3390/s150819006
31. Zakeri IF, Adolph AL, Puyau MR, Vohra FA, Butte NF. Cross-sectional time series and multivariate adaptive regression splines models using accelerometry and heart rate predict energy expenditure of preschoolers. J Nutr. 2013;143(1):114-22.https://doi.org/10.3945/jn.112.168542
32. Gnidovec B, Neubauer D, Zidar J. Actigraphic assessment of sleep-wake rhythm during the first 6 months of life. Clin Neurophysiol. 2002;113(11):1815-21.https://doi.org/10.1016/s1388-2457(02)00287-0
33. Cole RJ, Kripke DF, Gruen W, Mullaney DJ, Gillin JC. Automatic sleep/wake identification from wrist activity. Sleep. 1992;15(5):461-9.https://doi.org/10.1093/sleep/15.5.461
34. Galland BC, Kennedy GJ, Mitchell EA, Taylor BJ. Algorithms for using an activity-based accelerometer for identification of infant sleep-wake states during nap studies. Sleep Med. 2012;13(6):743-51.https://doi.org/10.1016/j.sleep.2012.01.018
35. Sadeh A, Acebo C, Seifer R, Aytur S, Carskadon MA. Activity-based assessment of sleep-wake patterns during 1st year of life. Infant Behav Dev. 1995;18(3):329--37.https://doi.org/10.1016/0163-6383(95)90021-7
36. Sadeh A, Lavie P, Scher A, Tirosh E, Epstein R. Actigraphic home-monitoring sleep-disturbed and control infants and young children: a new method for pediatric assessment of sleep-wake patterns. Pediatrics. 1991;87(4):494-9
